# Niraparib and abiraterone acetate plus prednisone for HRR-deficient metastatic castration-sensitive prostate cancer: a randomized phase 3 trial

**DOI:** 10.1038/s41591-025-03961-8

**Published:** 2025-10-07

**Authors:** Gerhardt Attard, Neeraj Agarwal, Julie N. Graff, Shahneen Sandhu, Eleni Efstathiou, Mustafa Özgüroğlu, Andrea J. Pereira de Santana Gomes, Karina Vianna, Hong Luo, Geoffrey T. Gotto, Heather H. Cheng, Won Kim, Carly R. Varela, Daneen Schaeffer, Kassie Kramer, Susan Li, Benoit Baron, Fei Shen, Suneel D. Mundle, Sharon A. McCarthy, David Olmos, Kim N. Chi, Dana E. Rathkopf

**Affiliations:** 1 Cancer Institute, University College London, London, UK; 2 Huntsman Cancer Institute, University of Utah, Salt Lake City, UT USA; 3 Oregon Health & Science University and Knight Cancer Institute, Portland, OR USA; 4 Veterans Affairs Portland Health Care System, Portland, OR USA; 5 Peter MacCallum Cancer Centre, Melbourne, Victoria Australia; 6 Houston Methodist Cancer Center, Houston, TX USA; 7 Istanbul University-Cerrahpaşa, Cerrahpaşa Faculty of Medicine, Istanbul, Türkiye; 8 Liga Norte Riograndense Contra o Cancer, Natal, Brazil; 9 Centro Integrado de Oncologia de Curitiba, Curitiba, Brazil; 10 Chongqing University Cancer Hospital, Chongqing, China; 11 Southern Alberta Institute of Urology, University of Calgary, Calgary, Alberta Canada; 12 University of Washington, Seattle, WA USA; 13 Fred Hutchinson Cancer Center, Seattle, WA USA; 14 Johnson & Johnson, Los Angeles, CA USA; 15 Johnson & Johnson, Spring House, PA USA; 16 Johnson & Johnson, Leiden, The Netherlands; 17 Johnson & Johnson, Raritan, NJ USA; 18 Instituto de Investigación Hospital 12 de Octubre, Madrid, Spain; 19 BC Cancer – Vancouver Center, University of British Columbia, Vancouver, British Columbia Canada; 20 Memorial Sloan Kettering Cancer Center and Weill Cornell Medicine, New York, NY USA

**Keywords:** Drug development, Prostate cancer

## Abstract

Inhibition of poly(ADP-ribose) polymerase (PARP) after relapse on hormone therapy is well established for patients with prostate cancer with homologous recombination repair (HRR) gene alterations, but resistance often develops. We hypothesized that PARP inhibition within 6 months of starting androgen deprivation therapy for metastatic castration-sensitive prostate cancer (mCSPC) could be effective and improve radiographic progression-free survival when added to standard-of-care treatments. The double-blind AMPLITUDE trial evaluated combining niraparib, a potent and specific PARP inhibitor, with abiraterone acetate and prednisone (AAP) versus placebo and AAP in mCSPC with HRR gene alterations. Patients (*n* = 696) were randomized in a 1:1 ratio (348 per group). Median age was 68 years; 56% had *BRCA1* or *BRCA2* alterations; 78% had high-volume metastases; and 16% had received docetaxel. The primary endpoint was met, with a significant improvement in radiographic progression-free survival observed first in the BRCA subgroup (median not reached at the time of analysis for the niraparib and AAP group versus 26 months for the AAP group; hazard ratio = 0.52; 95% confidence interval: 0.37–0.72; *P* < 0.0001) and then in the intention-to-treat population (hazard ratio = 0.63; 95% confidence interval: 0.49–0.80; *P* = 0.0001). The data for overall survival, a key secondary endpoint, are immature (193/389 events) but favor niraparib (hazard ratio = 0.79 (95% confidence interval: 0.59–1.04); BRCA subgroup: hazard ratio = 0.75 (95% confidence interval: 0.51–1.11)). Incidence of grade 3 or 4 adverse events was 75% in the niraparib and AAP group and 59% in the AAP group; most frequent in the niraparib and AAP group were anemia (29%), with 25% of patients requiring a blood transfusion, and hypertension (27%). There were 14 treatment-emergent adverse events leading to deaths in the niraparib group and seven in the placebo group. Combining niraparib with AAP significantly improved radiographic progression-free survival in patients with mCSPC harboring *BRCA1/BRCA2* or other HRR gene alterations, suggesting clinical benefit with this combination for these patients. ClinicalTrials.gov identifier: NCT04497844.

## Abstract (web summary)

In the randomized phase 3 AMPLITUDE trial, patients with HRR-deficient mCSPC experienced longer progression-free survival when they were treated with niraparib and AAP compared to placebo and AAP.

## Main

Prostate cancer is a heterogeneous disease, and benefit from therapy varies^1^. Adding the androgen biosynthesis inhibitor abiraterone acetate (hereafter referred to as abiraterone) plus prednisone or an androgen receptor antagonist to androgen deprivation therapy for mCSPC improves radiographic progression-free and overall survival^2–5^. However, patients can still have poor outcomes, and less than half are alive after 7 years^6^. Deleterious germline or somatic alterations in HRR genes are associated with poor prognosis and occur in up to 25% of patients, with *BRCA2* being the most commonly altered^7–11^. Loss of BRCA was first shown to sensitize cancers to inhibition of PARP via a process known as synthetic lethality^12,13^. Although most clinical studies confirm *BRCA1/BRCA2* loss cancers as sensitive, other genes biologically involved in HRR have been included in clinical trials for metastatic prostate cancer relapsing after castration (metastatic castration-resistant prostate cancer, mCRPC), with variable levels of efficacy^14–23^. The relatively low prevalence of individual gene alterations and the heterogeneity of biological sensitization can make interpretation of single-gene groups within individual trials challenging, but their inclusion has then allowed meta-analysis approaches to identify sensitive subgroups that can inform treatment pathways^24^.

Despite the efficacy (improvements in both radiographic progression-free survival and overall survival) reported for PARP inhibitor monotherapy in mCRPC^14,15,25^, resistance commonly occurs, often after emergence of secondary alterations in *BRCA2* that restore HRR function^26,27^. Concurrent inhibition of PARP and androgen receptor signaling may be synergistic^28–30^. This led to the development of combinations of PARP inhibitors and androgen receptor pathway inhibitors as a first-line treatment option for HRR-mutant mCRPC^16,17,21^. However, androgen receptor pathway inhibitors are now commonly used to treat mCSPC rather than mCRPC. Similarly, PARP inhibition has become established at diagnosis of advanced disease rather than at relapse in other cancer types; for example, in newly diagnosed advanced ovarian cancer as maintenance therapy after a complete or partial response to first-line platinum chemotherapy^31^. We therefore aimed to evaluate whether the combination of PARP inhibition and an androgen receptor pathway inhibitor at response to first-line androgen deprivation (castration-sensitive prostate cancer) would be effective.

Niraparib is a highly selective, potent PARP inhibitor^25^. Niraparib and abiraterone plus prednisone are approved for *BRCA1/2*-altered mCRPC^17–19,32,33^. We conducted the AMPLITUDE multinational phase 3 trial to determine whether the addition of niraparib to abiraterone plus prednisone results in longer radiographic progression-free survival in mCSPC with deleterious germline or somatic alterations in HRR genes. Key efficacy endpoint testing was conducted using a hierarchical graphical approach, first in the BRCA subgroup (*BRCA2* or *BRCA1*) and then in the HRR effector subgroup (that is, immediate effectors of HRR at DNA double-strand breaks (BRCA subgroup plus *BRIP1*, *PALB2*, *RAD51B* and *RAD54L*)) and then all intention-to-treat patients (HRR effector subgroup plus *CDK12*, *CHEK2* and *FANCA*) (Supplementary Fig. 1). To reduce timelines to drug approval, radiographic progression-free survival has been used as a regulatory endpoint for several phase 3 trials in mCSPC (for example, ARCHES (NCT02677896)^34^, TALAPRO-3 (NCT04821622)^35^ and CAPItello-281 (NCT04493853)^36^). Here we report the primary and final analysis of the AMPLITUDE trial primary endpoint, radiographic progression-free survival, and the first interim analysis of secondary endpoints, including overall survival.

## Results

### Patients

**Fig. 1:**
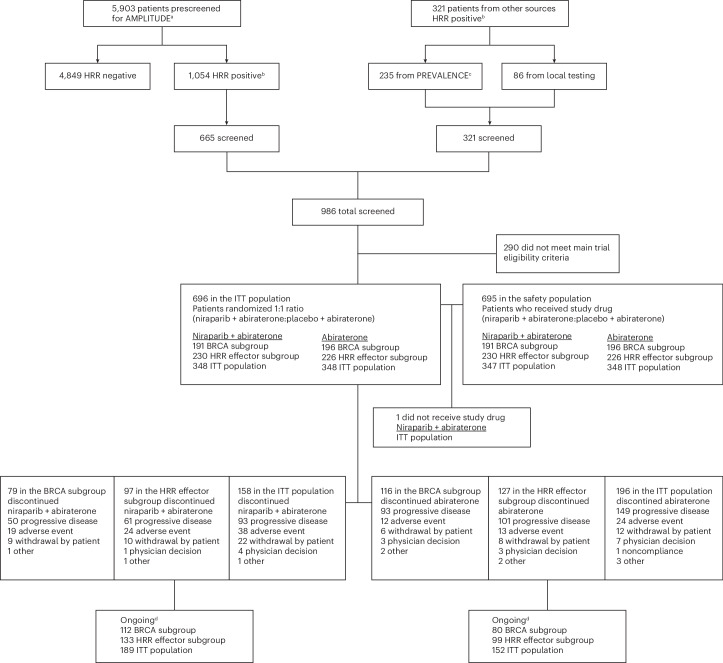
CONSORT diagram. Flow diagram showing participant recruitment. ^a^Includes patients with a valid result (that is, HRR negative or HRR positive per protocol). ^b^HRR positivity based on assay results and presence of protocol-defined HRR alterations in *BRCA1/2*, *BRIP1*, *CHEK2*, *CDK12*, *FANCA*, *PALB2*, *RAD51B* and *RAD54L*. ^c^PREVALENCE is a prospective trial to determine the prevalence of DNA repair defects in patients with advanced prostate cancer. ^d^‘Ongoing’ refers to patients continuing to receive trial treatment at the clinical cutoff data. ITT, intention-to-treat.

A total of 5,903 patients were prescreened by clinical next-generation sequencing of tumor or plasma or germline DNA by one of the central assays in the AMPLITUDE study and had valid results. Of these, 1,054 were positive for at least one protocol-defined HRR gene alteration, and 665 entered screening. In addition, 235 biomarker-positive participants were approved for screening by the sponsor based on a positive result in the PREVALENCE study (NCT03871816), a prospective evaluation of the prevalence of HRR gene alterations that had an overlapping period of recruitment and the same sponsor as the AMPLITUDE trial^37^. An additional 86 patients were included based on positive preapproved local tests. A total of 696 patients were randomized from 21 December 2020 to 20 July 2023, with 348 assigned to niraparib and abiraterone plus prednisone (niraparib and abiraterone group) and 348 to placebo and abiraterone (abiraterone group) (Fig. 1). In all, 387 participants (56%) had a *BRCA1* or *BRCA2* gene alteration and 456 (66%) were in the HRR effector subgroup.

At the clinical data cutoff date (7 January 2025) for the first and final analysis for radiographic progression-free survival and after 264 events, the median follow-up time was 30.8 months. The median duration of treatment was 25.3 months in the niraparib and abiraterone group and 22.5 months in the abiraterone group. The median treatment compliance was 97.0% in the niraparib and abiraterone group and 99.3% in the abiraterone group, with 89.9% of participants in the niraparib and abiraterone group and 94.8% of patients in the abiraterone group taking more than 80% of prescribed tablets. A total of 54% in the niraparib and abiraterone group and 44% in the abiraterone group continued to receive the trial intervention.

The baseline demographic and disease characteristics were well balanced between the two groups (Table 1), including the BRCA and HRR effector subgroups (Extended Data Table 1). Previous therapies for prostate cancer are in Extended Data Table 2.

**Table 1:** Demographics and disease characteristics at baseline in the intention-to-treat population^a^

	Niraparib plus abiraterone (*n* = 348)	Abiraterone (*n* = 348)
Median age (range), years	68 (40–88)	67 (40–92)
Race, *n* (%)		
White	246 (70.7)	257 (73.9)
Asian	77 (22.1)	67 (19.3)
Black or African American	18 (5.2)	10 (2.9)
Other	3 (0.9)	6 (1.7)
Not reported/unknown	4 (1.1)	8 (2.3)
Region, *n* (%)		
Europe	168 (48.3)	177 (50.9)
Asia	72 (20.7)	63 (18.1)
North America	45 (12.9)	44 (12.6)
Rest of world	63 (18.1)	64 (18.4)
ECOG performance status score, *n* (%)^b^		
0	242 (69.5)	218 (62.6)
1	97 (27.9)	124 (35.6)
2	9 (2.6)	6 (1.7)
Gleason score at initial diagnosis, *n* (%)^c^		
≤7	60 (17.2)	68 (19.5)
>7	276 (79.3)	262 (75.3)
Unknown	12 (3.4)	18 (5.2)
Metastatic stage at initial diagnosis, *n* (%)		
Non-metastatic	32 (9.2)	36 (10.3)
Metastatic	301 (86.5)	302 (86.8)
Unknown	15 (4.3)	10 (2.9)
Metastatic disease volume at start of androgen deprivation therapy, *n* (%)		
High	269 (77.3)	271 (77.9)
Low	79 (22.7)	77 (22.1)
Median time from start of androgen deprivation therapy for metastatic disease (range), months	2.46 (0.2–6.2)	2.30 (0.1–6.2)
Median PSA level at initial diagnosis (range), μg l^−1^	112.3 (0.1–17,475)	101.6 (0.1–15,900)
Median PSA level at baseline (range), μg l^−1 (^^d)^	2.74 (0–8,046)	3.57 (0–2,703)
Mean baseline BPI-SF pain score, *n* (%)	*n* = 348	*n* = 346
0: no pain	149 (42.8)	152 (43.9)
1–3: mild pain	118 (33.9)	117 (33.8)
>3: moderate to severe pain	81 (23.3)	77 (22.3)
Single gene alterations, *n* (%)^e^		
*BRCA2*	148 (42.5)	144 (41.4)
*BRCA1*	25 (7.2)	25 (7.2)
Other	149 (42.8)	147 (42.2)

^a^Percentages may not total 100 because of rounding. ^b^ECOG performance status scores range from 0 to 5, with higher scores reflecting greater disability. ^c^Gleason scores range from 2 to 10, with higher scores indicating higher-grade cancer that may be more aggressive. ^d^Patients were allowed to be on ongoing androgen deprivation therapy; therefore, prostate-specific antigen (PSA) levels were lower than at diagnosis. ^e^All gene alterations are shown in Extended Data Table 1. BFI-SF, Brief Pain Inventory (Short Form); ECOG, Eastern Cooperative Oncology Group.

### Efficacy: primary endpoint

**Fig. 2:**
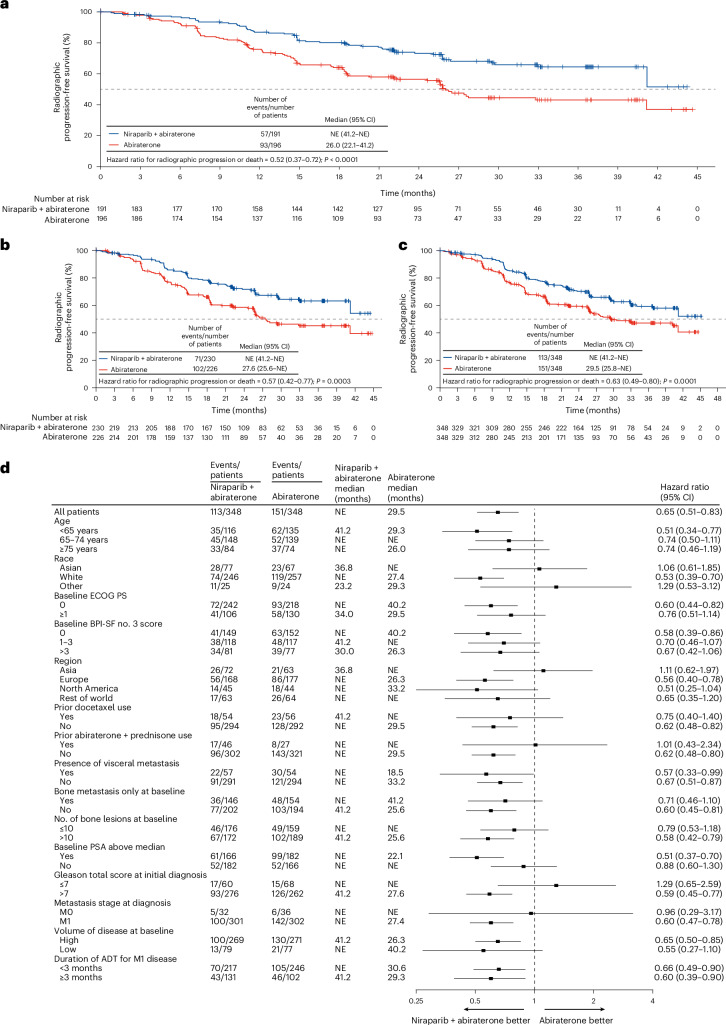
Radiographic progression-free survival. **a**−**c**, Kaplan−Meier estimates of investigator-assessed radiographic progression-free survival in the BRCA subgroup (*n* = 387) (**a**), the HRR effector subgroup (*n* = 456) (**b**) and the intention-to-treat population (*n* = 696) (**c**). Radiographic progression-free survival was compared between treatment groups using the stratified log-rank test. Hazard ratios and 95% confidence intervals (CIs) were estimated by stratified Cox proportional hazards models. Forest plot of prespecified subgroup analysis in the intention-to-treat population; dots represent hazard ratio estimates, and whiskers represent 95% CIs (**d**). Vertical bars are censor marks. ADT, androgen deprivation therapy; BFI-SF, Brief Pain Inventory (Short Form); NE, not estimable; PS, performance status.

**Extended Data Fig. 1:**
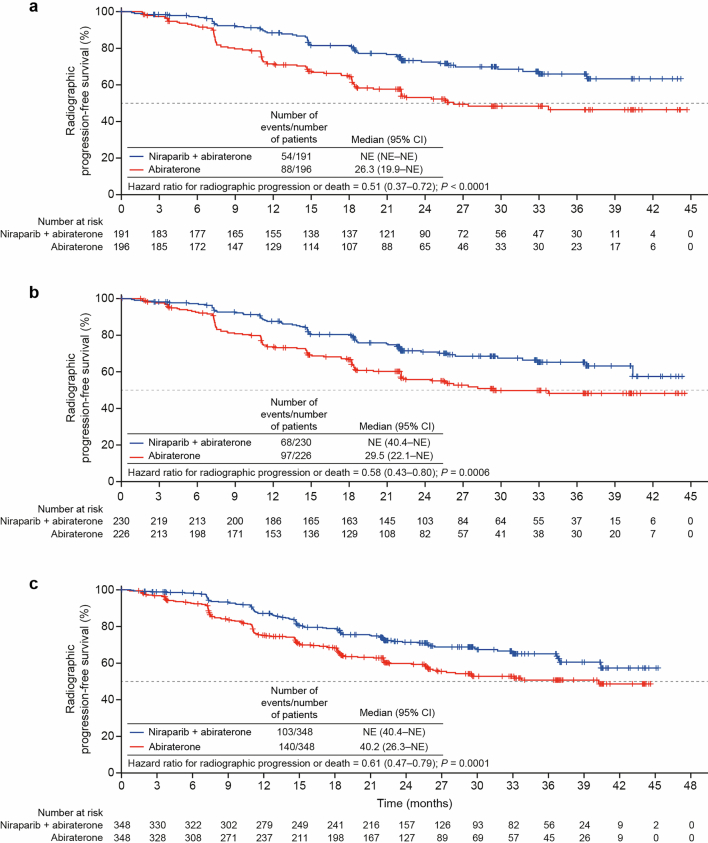
Kaplan-Meier estimates of radiographic progression-free survival in the (a) BRCA subgroup (n = 387), (b) HRR effector subgroup (n = 456) and (c) intention-to-treat population (n = 696) by blinded independent central review. Radiographic progression-free survival was compared between treatment groups using the stratified log-rank test. HRs and 95% CIs were estimated by stratified Cox proportional-hazards models. Vertical bars are censor marks. NE, not estimable.

In the first hierarchical test for efficacy, in the BRCA subgroup, treatment with niraparib and abiraterone resulted in significant improvement in the primary endpoint of investigator-assessed radiographic progression-free survival compared to abiraterone (hazard ratio = 0.52 (95% confidence interval: 0.37–0.72); *P* < 0.0001) (Fig. 2a). Median radiographic progression-free survival in the BRCA subgroup was not reached in the niraparib and abiraterone group and was 26.0 months in the abiraterone group. Patients in the niraparib and abiraterone group had a significantly lower risk of radiographic progression or death in both the HRR effector subgroup (hazard ratio = 0.57 (95% confidence interval: 0.42–0.77); *P* = 0.0003) (Fig. 2b) and the intention-to-treat population (hazard ratio = 0.63 (95% confidence interval: 0.49–0.80); *P* = 0.0001) (Fig. 2c). Median radiographic progression-free survival in both the HRR effector subgroup and the intention-to-treat population was not reached in the niraparib and abiraterone group and was 27.6 months and 29.5 months, respectively, in the abiraterone group. The treatment effect of niraparib and abiraterone plus prednisone was consistent across the majority of prespecified subgroups, although the small number of events within certain subgroups precludes definitive interpretation (Fig. 2d). The magnitude of the benefit in the niraparib and abiraterone group for radiographic progression-free survival as assessed by blinded independent central review was as large as with investigator assessment (Extended Data Fig. 1).

Although this was not a prespecified analysis, we were interested in determining the effect in patients with HRR gene alterations and no detectable *BRCA2* or *BRCA1* alteration (that is, a gene alteration in any of *BRIP1*, *PALB2*, *RAD51B*, *RAD54L*, *CDK12*, *CHEK2* and *FANCA*). The radiographic progression-free survival in patients without *BRCA1/2* alterations showed a hazard ratio of 0.81 (95% confidence interval: 0.56–1.18).

### Secondary endpoints

**Fig. 3:**
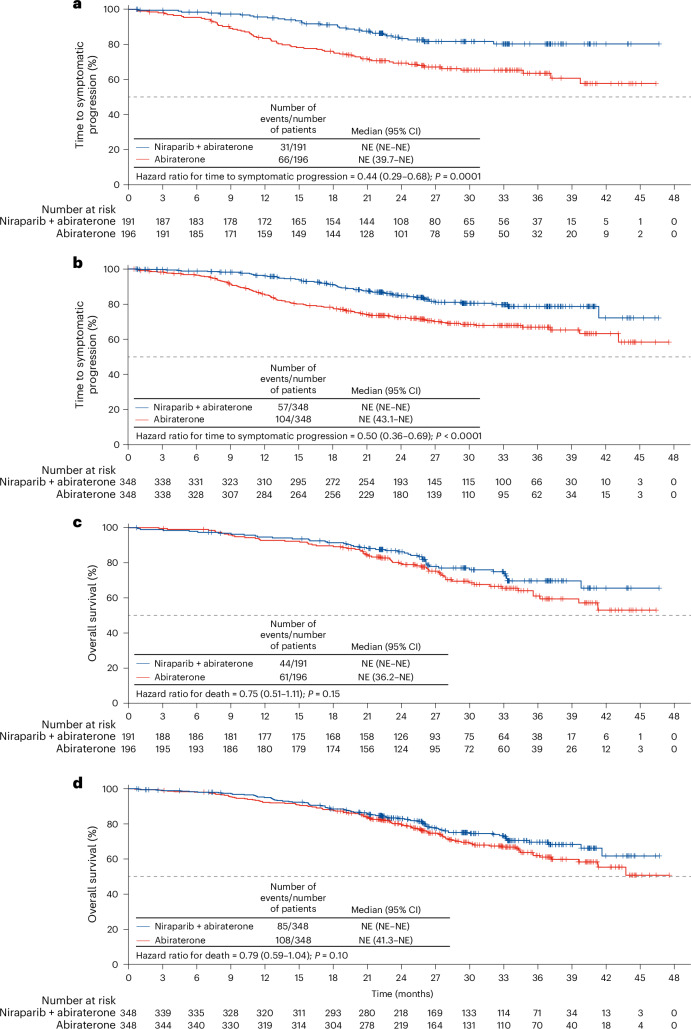
Secondary endpoints. **a**−**d**, Kaplan−Meier estimates of time to symptomatic progression (**a**,**b**) and overall survival (**c**,**d**) in the BRCA subgroup (*n* = 387) (**a**,**c**) and the intention-to-treat population (*n* = 696) (**b**,**d**) ordered as per the sequence for efficacy testing conducted in the hierarchical graphical approach. Comparisons between treatment groups used the stratified log-rank test. Hazard ratios and 95% confidence intervals were estimated by stratified Cox proportional hazards models. To be concise, Kaplan−Meier estimates in the HRR effector subgroup are in Extended Data Fig. 2. Vertical bars are censor marks.

**Extended Data Fig. 2:**
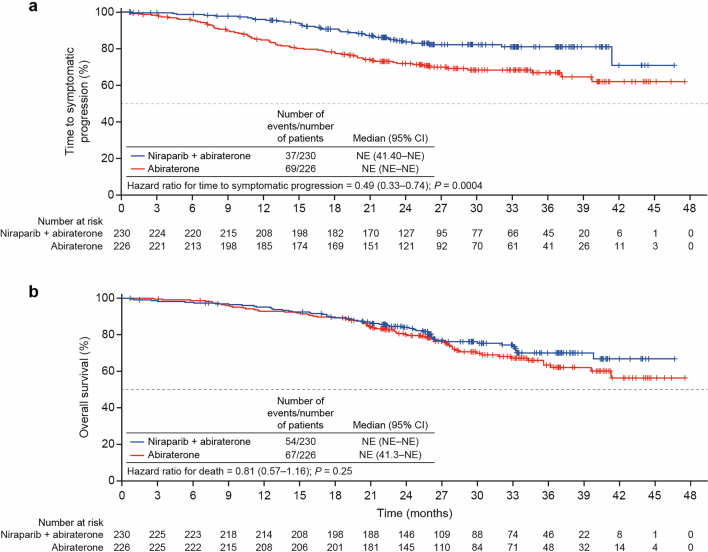
Kaplan-Meier estimates of time to symptomatic progression (a) and overall survival (b) in the HRR effector subgroup (n = 456). Comparison between treatment groups was performed using the stratified log-rank test. HRs and 95% CIs were estimated by stratified Cox proportional-hazards models. Vertical bars are censor marks.

Time to symptomatic progression and overall survival were key endpoints included in the graphical approach for testing efficacy. In the niraparib and abiraterone group, significant improvements were observed in time to symptomatic progression in the BRCA subgroup (hazard ratio = 0.44 (95% confidence interval: 0.29–0.68); *P* = 0.0001; Fig. 3a and Extended Data Table 3) and the intention-to-treat population (hazard ratio = 0.50 (95% confidence interval: 0.36–0.69); *P* < 0.0001 (Fig. 3b and Extended Data Table 3)). The improvement in time to symptomatic progression in the HRR effector subgroup was consistent and is shown in Extended Data Fig. 2a and Extended Data Table 3.

At this time, the first interim analysis for overall survival was also conducted. Most patients remained alive; of a target of 389 events required for the final overall survival analysis, 193 patients had died (an information fraction of 50%)—85 of 348 (24%) in the niraparib and abiraterone group and 108 of 348 (31%) in the abiraterone group. Overall survival analysis estimates a 25% reduction in risk of death in the BRCA subgroup (hazard ratio = 0.75 (95% confidence interval: 0.51–1.11); *P* = 0.15 (Fig. 3c and Extended Data Table 3)). This was the first test in the graphical approach for testing efficacy that was not significant. The estimates were similar in the HRR effector subgroup and are presented in Extended Data Fig. 2b and Extended Data Table 3. In the intention-to-treat population, the hazard ratio estimate for overall survival was 0.79 (95% confidence interval: 0.59–1.04); *P* = 0.10 (Fig. 3d and Extended Data Table 3).

Of the 196 patients in the abiraterone group who discontinued treatment, at the time of the clinical data cutoff 141 (72%) were reported to have received a subsequent treatment known to be life prolonging for prostate cancer that was chosen based on the treating physician’s judgment and local approvals, including chemotherapy for 102 of 141 patients (72%) and a PARP inhibitor for 47 of 141 patients (33%) (Extended Data Table 4). Among the 102 patients who received chemotherapy, 18 (17.6%) received carboplatin or cisplatin, which have a similar mechanism of action as PARP inhibitors. Of the 159 patients who were not receiving trial treatment any longer in the niraparib and abiraterone group (158 discontinued and one did not start), 89 (56%) were reported to have received a subsequent treatment known to be life prolonging, including chemotherapy for 71 of 89 (80%) and a PARP inhibitor for 10 (11%). Double-blinding prior to radiographic progression has been maintained to allow ongoing follow-up of overall survival.

Other secondary endpoint testing is considered exploratory. Time to subsequent therapy was improved in the niraparib and abiraterone group compared to the abiraterone group in the intention-to-treat population (hazard ratio = 0.54 (95% confidence interval: 0.41–0.70); nominal *P* < 0.0001; Extended Data Table 3) as well as the BRCA and HRR effector subgroups (Extended Data Table 3).

### Other prespecified endpoints

Second progression-free survival was also longer in the niraparib and abiraterone group (median not reached) than in the abiraterone group (median 44.0 months; hazard ratio = 0.66 (95% confidence interval: 0.51–0.86); nominal *P* = 0.002; Extended Data Table 3). The objective response rate was similar in the niraparib and abiraterone group (72%, 76 of 106 with measurable disease at baseline) and the abiraterone group (74%, 81 of 110), but the duration of response in patients with complete or partial response was longer in the niraparib and abiraterone group (hazard ratio = 0.55 (95% confidence interval: 0.35–0.86); nominal *P* = 0.008). The time to PSA progression was improved in the niraparib and abiraterone group compared to the abiraterone group (hazard ratio = 0.50 (95% confidence interval: 0.39–0.65); nominal *P* < 0.0001 (Extended Data Table 3)).

**Fig. 4:**
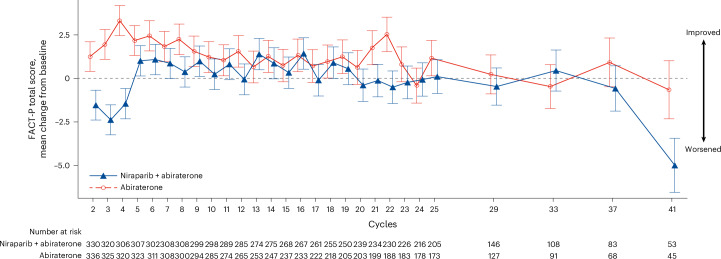
Patient-reported outcomes. Median and s.e. estimates (vertical bars) are presented for the least-squares mean change from baseline in FACT-P total score in the intention-to-treat population. All timepoints when measurements were collected (as defined in the protocol) are presented. Only patients with patient-reported outcome assessments are included in niraparib plus abiraterone (*n* = 330) and abiraterone (*n* = 336) treatment groups.

Health-related quality of life Functional Assessment of Cancer Therapy−Prostate (FACT-P) scores from cycle 2 showed an improvement when compared to baseline in the abiraterone group but an initial decline at cycles 2, 3 and 4 compared to baseline in the niraparib and abiraterone group (Fig. 4). Health-related quality of life FACT-P scores improved in the niraparib and abiraterone group by cycle 5, with no difference observed compared to the abiraterone group at this time and up to cycle 37 (Fig. 4).

### Safety

Grade 3 or 4 adverse events were observed in 261 of 347 patients (75.2%) in the niraparib and abiraterone group and in 205 of 348 patients (58.9%) in the abiraterone group (Extended Data Table 5). Serious adverse events occurred in 136 patients (39.2%) in the niraparib and abiraterone group and in 96 patients (27.6%) in the abiraterone group. Treatment discontinuations due to adverse events occurred in 51 patients (14.7%) in the niraparib and abiraterone group, including one case of myelodysplastic syndrome (in a patient with a *CHEK2* germline mutation), and in 36 patients (10.3%) in the abiraterone group (Extended Data Table 6). Dose reductions occurred in 76 patients (21.9%) in the niraparib and abiraterone group and in 24 patients (6.9%) in the abiraterone group (Supplementary Table 1a). Dose interruptions occurred in 232 patients (66.9%) in the niraparib and abiraterone group and in 147 patients (42.4%) in the abiraterone group (Supplementary Table 1b). Treatment-emergent adverse events leading to death occurred in 14 patients in the niraparib and abiraterone group and in seven patients in the abiraterone group (Extended Data Table 7). In the niraparib and abiraterone group, causes of death included four cases of respiratory infection, including two attributed as related to COVID-19, four attributed to cardiac causes and three classified as sudden death. The other three deaths were one each of sepsis, subdural hematoma and multiorgan dysfunction syndrome. The most common adverse events are shown in Table 2. The most common grade 3 or 4 adverse events were anemia (29.1% versus 4.6%) and hypertension (26.5% versus 18.4%) in the niraparib and abiraterone group and the abiraterone group, respectively. In the niraparib and abiraterone group, 87 patients (25.1%) required at least one transfusion for anemia (median among patients requiring a transfusion: 2, range: 1–5) compared to 13 (3.7%) in the abiraterone group (median among patients requiring a transfusion: 2, range: 1–3).

**Table 2:** Individual adverse events^a^

	Niraparib plus abiraterone (*n* = 347)		Abiraterone (*n* = 348)	
	All grades	Grade ≥3	All grades	Grade ≥3
**Events reported in** ≥**15% of patients in either group,** ***n*** **(%)**				
Anemia^b^	179 (51.6)	101 (29.1)	83 (23.9)	16 (4.6)
Hypertension^b^	152 (43.8)	92 (26.5)	113 (32.5)	64 (18.4)
Constipation	122 (35.2)	0	57 (16.4)	1 (0.3)
Nausea	107 (30.8)	0	50 (14.4)	0
Fatigue	91 (26.2)	7 (2.0)	64 (18.4)	4 (1.1)
Hypokalemia^b^	90 (25.9)	40 (11.5)	70 (20.1)	38 (10.9)
Neutropenia^b^	76 (21.9)	33 (9.5)	28 (8.0)	7 (2.0)
Arthralgia	73 (21.0)	2 (0.6)	74 (21.3)	6 (1.7)
Back pain	68 (19.6)	12 (3.5)	77 (22.1)	5 (1.4)
Thrombocytopenia^b^	66 (19.0)	24 (6.9)	20 (5.7)	1 (0.3)
COVID-19	65 (18.7)	3 (0.9)	71 (20.4)	4 (1.1)
Hot flush	63 (18.2)	0	48 (13.8)	0
Leukopenia	58 (16.7)	16 (4.6)	18 (5.2)	1 (0.3)
Vomiting	56 (16.1)	3 (0.9)	30 (8.6)	0
Peripheral edema^b^	55 (15.9)	1 (0.3)	42 (12.1)	0
Weight decreased	53 (15.3)	4 (1.2)	18 (5.2)	0
Alanine aminotransferase increased^b^	22 (6.3)	6 (1.7)	54 (15.5)	17 (4.9)

^a^Shown are adverse events of any cause that occurred from the time of the first dose of the trial intervention through 30 days after the last dose, according to preferred term and highest grade according to National Cancer Institute Common Terminology Criteria for Adverse Events version 5. Patients are counted only once for any given event, regardless of the number of times that they experienced the event. The worst toxicity event experienced by the patient was used. If a patient had a missing toxicity grade for a specific adverse event, the patient is counted in the total column for that adverse event. Adverse events are coded using MedDRA version 27.1. ^b^Identified as predefined adverse events of special interest.

## Discussion

This phase 3 trial used next-generation sequencing to identify patients with mCSPC with a somatic or germline alteration in *BRCA2* or *BRCA1* (56% of patients) or at least one of seven other genes proposed to be involved in HRR (*BRIP1*, *PALB2*, *RAD51B*, *RAD54L*, *CDK12*, *CHEK2* and *FANCA*). Most patients (87%) had synchronous metastases at diagnosis that may relate to the more aggressive characteristics of this molecularly selected population. Two subgroupings of interest (BRCA and HRR effectors) were prespecified for efficacy analyses, additional to the intention-to-treat population. Niraparib and abiraterone resulted in significantly longer radiographic progression-free survival than abiraterone across these three prespecified populations, meeting the primary endpoint. Clinical benefit was further supported by statistically significant and clinically meaningful improvements in time to symptomatic progression.

The median radiographic progression-free survival time (29.5 months) in the abiraterone group was shorter than was anticipated at trial inception (33 months), emphasizing the significant unmet need of patients with HRR gene alterations. The magnitude of benefit for both radiographic progression-free survival and overall survival was numerically greater in the BRCA subgroup than in the intention-to-treat population. This is consistent with prior reports of probable greatest benefit for patients with *BRCA1/2* alterations treated with PARP inhibitors for mCRPC^14,16,17^. Although there could be treatment efficacy in other HRR gene types, this may be heterogeneous.

A drug−drug interaction was observed between niraparib and apalutamide but not with abiraterone, which was, therefore, chosen for combination with niraparib to minimize variability in systemic exposure^38^. With a longer duration of dosing in the AMPLITUDE trial compared to previous trials^17,25^, the safety results of niraparib in combination with abiraterone remained consistent with previous observations in patients with mCRPC^17,18^. In the AMPLITUDE trial, treatment is administered to progression. Grade 3 or higher toxicities were observed in the majority of patients in the niraparib and abiraterone group, largely driven by anemia and hypertension, and there were more treatment-emergent adverse events leading to death (*n* = 14) than in the abiraterone group (*n* = 7). Additionally, there was one case of myelodysplastic syndrome in the niraparib and abiraterone group, and close monitoring will continue due to the known risk of myelodysplastic syndrome associated with PARP inhibitors^39,40^. Patient-reported outcomes in the niraparib and abiraterone group identify an initial decline from baseline and then minimal or no noticeable difference from cycle 5. Differences at late timepoints require further evaluation and may relate to imbalances in the number of patients remaining in the two groups. Although equipoise may remain on initiating niraparib for mCSPC, the limited detriment on long-term quality of life could be reassuring.

At this first interim analysis of overall survival (secondary endpoint), the data were not sufficiently mature to detect a significant improvement with niraparib and abiraterone at the treatment effect that we are observing. Improved second progression-free survival was also observed despite extensive use of subsequent life-prolonging therapies. This was a double-blind trial, and there was no formal crossover to PARP inhibition at progression in the patients in the abiraterone group. At this first analysis, a PARP inhibitor was used for 33% of patients who discontinued treatment in the abiraterone group and then received a life-prolonging agent. A subset of patients (12.8% in the abiraterone group who received subsequent therapy) was treated with platinum chemotherapy.

Patients were allowed to have received docetaxel prior to trial entry, when deemed appropriate by the treating physician, to reflect the most current treatment recommendations and other global guidelines^41^. Therefore, patients treated in the experimental group could have received up to four effective treatments in the metastatic castration-sensitive setting (niraparib, abiraterone plus prednisone, docetaxel and androgen deprivation therapy). In total, 16% received docetaxel, and, given the trial design and lack of combination data with niraparib, this had to have been completed prior to the start of niraparib and abiraterone. In the prespecified subgroup analysis, noting the limitation of the small size of the group of patients who had received previous docetaxel, the direction of treatment effect is consistent regardless of previous docetaxel use.

Our study has some limitations. Only the BRCA and HRR effector gene subgroups were powered for formal statistical testing, as the number of patients in each of the other seven individual gene subgroups was too small. Further assessment of individual gene alteration effects could be achieved by combined analyses with other trials and clinical datasets^24^. Our study allowed only limited previous androgen receptor pathway inhibition, namely up to 45 days of abiraterone plus prednisone prior to randomization. Due to the timelines for obtaining genetic test results in the setting of a trial, a proportion of HRR-positive patients who were prescreened for AMPLITUDE were not eligible to proceed. The study results now support HRR gene testing as routine practice for mCSPC and, consequently, better integration of testing into clinical practice, which could allow for earlier availability of gene results. Given that *BRCA2* is the largest single-gene subgroup and, at the time of trial design, had the best evidence for sensitivity to PARP inhibition in prostate cancer, it was considered important to balance the presence of *BRCA2* across both treatment groups and so *BRCA2* was included as a stratification factor. However, data that emerged after initiation of accrual justified grouping of *BRCA2* and *BRCA1* for efficacy analyses. Given that alterations involving solely *BRCA1* are relatively uncommon, this difference between the stratification factor and subgroup analysis in *BRCA1/2* does not affect the overall result. Our trial enrolled patients with metastatic disease on conventional imaging. A substantial population of patients is emerging who have metastatic disease on newer imaging, such as prostate-specific membrane antigen positron emission tomography scans, but none on the imaging required for our trial. Future studies could be considered that evaluate the efficacy of PARP inhibition in the population of patients with mCSPC defined by new imaging techniques^42^. Subsequent therapies were not mandated by the protocol and were administered at the discretion of the investigator, including PARP inhibitors based on local approvals for mCRPC. Although the rate of subsequent use of PARP inhibitors in AMPLITUDE is higher than previously reported in placebo-controlled PARP inhibitor trials in mCRPC^16,18^, possibly explained by the allowance for unblinding upon investigator request once radiographic progression was documented, the lack of systematic crossover by trial design may not allow definite confirmation that the relatively longer duration of niraparib exposure in mCSPC improves survival when compared to shorter exposure to PARP inhibition for mCRPC. This could be explored further at final trial analysis for overall survival.

In conclusion, this is, to our knowledge, the first demonstration of efficacy of a PARP inhibitor in mCSPC. The combination of niraparib and abiraterone plus prednisone was associated with significantly longer radiographic progression-free survival in patients with mCSPC with HRR gene alterations. Adverse events were medically manageable with dose modifications and supportive care; there were few treatment discontinuations; and associated serious sequalae were rare. The reduction in radiographic progression is clinically significant, most notably in cancers with a *BRCA1/2* mutation, and this combination could represent a new treatment option for such patients. The potential benefits of the prolonged radiographic progression-free survival with this treatment regimen should be considered in the context of the potential risks of adverse events in this population of patients with mCSPC.

## Methods

### Trial design and participants

AMPLITUDE is an ongoing randomized, phase 3, double-blind, placebo-controlled trial in patients with HRR gene-altered mCSPC conducted at 204 medical centers from 32 countries globally, including countries in Europe, Asia and North America (list of investigators included in the Supplementary Information). The protocol was approved by the review board at each participating institution and health authorities in every participating country; the protocol can be found in the Supplementary Information. The trial was conducted in accordance with the International Counsel for Harmonisation guidelines for Good Clinical Practice and the principles of the Declaration of Helsinki. All patients provided written informed consent. Participants were not paid for taking part in this study but may have received stipends for expenses directly related to study visits, such as local travel, meals and parking.

This study was sponsored by Johnson & Johnson. The trial was designed by the sponsor with input from the trial steering committee and is registered in ClinicalTrials.gov (NCT04497844). The sponsor commissioned an independent data monitoring committee to monitor safety on an ongoing basis. The sponsor also designated an independent biostatistician to provide data to the independent data monitoring committee, which ensured that the study was conducted safely.

Study site personnel transcribed the data from source documents into electronic case report forms. All participating institutions have agreements with the sponsor regarding data confidentiality.

### Participants

Eligible male patients were aged 18 years or older, had an ECOG performance status score of 0–2 and had at least one deleterious HRR gene alteration on central testing of tumor tissue (FoundationOne CDx; Foundation Medicine), plasma (FoundationOne Liquid CDx; Foundation Medicine) or germline (Invitae Multi-Cancer Panel; Invitae). Positive test results were also permitted from sponsor-approved local tests or from the PREVALENCE study (NCT03871816)^37^. Eligible HRR genes were *BRCA1*, *BRCA2*, *BRIP1*, *PALB2*, *RAD51B*, *RAD54L*, *CDK12*, *CHEK2* and *FANCA*.

### Trial treatment

Patients were randomly assigned in a 1:1 ratio to receive a dual-action tablet of niraparib (200 mg) and abiraterone acetate (1,000 mg) plus prednisone (5 mg) orally once daily (niraparib and abiraterone group) or matched placebo and abiraterone acetate tablets plus prednisone (abiraterone group) continuously in 28-day cycles. Randomization was done using permuted block randomization managed via an interactive web randomization system. Patients were stratified according to HRR gene (*BRCA2* alteration versus *CDK12* alteration versus others), previous docetaxel (yes versus no) and metastases volume (high versus low) defined as described previously^43^. Crossover between trial groups was not specified in the protocol. After discontinuation of the trial intervention, patients could receive treatments at the investigator’s discretion. Unblinding was allowed upon request after confirmed investigator-assessed radiographic progression to support subsequent treatment decisions.

### Procedures

Documentation of metastatic disease (soft tissue lesions by computed tomography or magnetic resonance imaging (MRI) or bone lesions by technetium-99m bone scan) was required. Androgen deprivation therapy must have started at least 14 days and no longer than 6 months prior to randomization and was continued during study treatment. Up to six cycles of docetaxel were allowed for mCSPC, completed within 3 months of randomization. Palliative radiotherapy was required to be completed before randomization. Also, up to 45 days of abiraterone plus prednisone prior to randomization was permitted. Additional eligibility requirements are listed in the Supplementary Methods.

Radiographic assessments (computed tomography or MRI of chest, abdomen and pelvis and technetium-99m bone scan) were done at baseline and within 7 days of day 1 of cycle 3, cycle 5 and then every four cycles until radiographic progression.

Adverse events were assessed according to National Cancer Institute Common Terminology Criteria for Adverse Events version 5. FACT-P assessments were collected on day 1 of cycles 1− 24 and then every 4 months from month 25 to 12 months after discontinuation of trial medication.

### Trial endpoints

The primary outcome measure was investigator-assessed radiographic progression-free survival, defined as time from randomization to radiographic progression or death, whichever occurred first. Soft tissue progression was defined in Response Evaluation Criteria in Solid Tumors version 1.1, and bone disease progression was based on Prostate Cancer Working Group 3 criteria, requiring confirmation by a second scan ≥6 weeks later^44^. Secondary outcomes were time to symptomatic progression, overall survival, time to subsequent therapy and safety. Time to symptomatic progression was defined as the time from randomization to a clinically relevant cancer-related symptom event that required an intervention, including radiation for bone pain, nephrostomy for urinary obstruction, cord compression or the start of a new subsequent treatment. Other endpoints were second progression-free survival, defined as the time from randomization to radiographic, clinical or PSA progression after the first subsequent therapy, objective response rate, duration of response, time to PSA progression, PSA response rate and patient-reported outcomes for health-related quality of life (FACT-P questionnaire)^45^ (all defined in the Supplementary Information)^45^.

### Statistical analysis

It was estimated that approximately 692 patients were to be randomized to observe the 261 radiographic progression events or deaths required to provide 91% power to detect a hazard ratio of 0.64 at a two-sided significance level of 0.02475 for the final analysis (no interim) of radiographic progression-free survival in all patients. We assumed that radiographic progression-free survival follows an exponential distribution, with a median of 33 months in the abiraterone group, equivalent to a constant hazard ratio of approximately 0.021 per month. We assumed a 33-month accrual period followed by approximately 15 months of follow-up. Additionally, approximately 146 radiographic progression-free survival events were projected to be observed in the target BRCA subgroup of patients at the time of the radiographic progression-free survival primary analysis, providing 95% power to detect a hazard ratio of 0.55 for radiographic progression-free survival at a two-sided significance level of 0.05.

Finally, based on this sample size, 389 overall survival events will be required at the time of final overall survival analysis after a study duration of approximately 79 months to provide 80% power to detect an underlying true hazard ratio of 0.75 for overall survival at a two-sided significance level of 0.05. We assumed that overall survival follows an exponential distribution, with a median of 53 months in the abiraterone group. We will analyze overall survival using a group sequential design according to Kim−DeMets alpha spending function with parameters of 2.5 over two interim analyses and one final analysis.

Efficacy data were analyzed on an intention-to-treat basis. The first interim analyses of overall survival and time to symptomatic progression were planned at the time of final analysis for radiographic progression-free survival and following the planned graphical testing framework with group sequential design (Supplementary Fig. 1). Based on this hierarchical multiple comparison testing procedure, the overall family-wise type I error rate was preserved at the prespecified two-sided 0.05 level, starting with comparing radiographic progression-free survival in the BRCA subgroup, then in the HRR effector gene subgroup and, finally, in the intention-to-treat population followed by comparing time to symptomatic progression and then overall survival, in the same population order (Supplementary Fig. 1). For radiographic progression-free survival, time to symptomatic progression and overall survival, only hypotheses where some type I error was allocated per the graphical approach were planned to be reported. Testing for treatment by subgroup interaction in subgroup analyses was not performed as the power for such a test was too low. Medidata version 2024.2.0 was used for data collection; East version 6.5 was used for sample size calculations; and SAS version 9.4 was used for data analyses.

Demographic and clinical characteristics at baseline were summarized with descriptive statistics. For time-to-event variables, the Kaplan−Meier method, stratified Cox proportional hazards model and stratified log-rank test were used to estimate the medians, hazard ratios and their associated 95% confidence intervals and *P* values. Safety analysis included all patients who received at least one dose. Least-squares mean change from baseline of FACT-P scores was estimated from a mixed-effects repeated-measures model, with the FACT-P total score measured at each post-baseline assessment as the dependent variable. The mixed-effects model assumed FACT-P baseline score, treatment, visit time and treatment-by-visit time interaction as fixed effects and patients as random effect. In the absence of meaningful imbalance in baseline covariates between treatment arms, baseline covariates were not added to the model. To account for the correlation between repeated measurements observed over time, a compound symmetry covariance structure was used in the model. A data-as-observed approach was used to handle missing data.

### Reporting summary

Further information on research design is available in the Nature Portfolio Reporting Summary linked to this article.

## Online content

Any methods, additional references, Nature Portfolio reporting summaries, source data, extended data, supplementary information, acknowledgements, peer review information; details of author contributions and competing interests; and statements of data and code availability are available at 10.1038/s41591-025-03961-8.

## Supplementary information

Supplementary Information AMPLITUDE study investigators, supplementary methods, Supplementary Fig. 1, Supplementary Table 1 and protocol

Reporting Summary

## Figures and Tables

**Extended Data Table 1:**
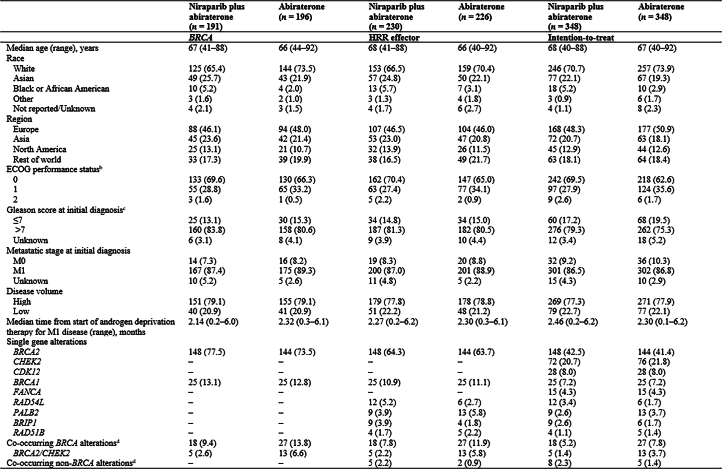
Demographics and baseline disease characteristics, expanded list, in the BRCA subgroup, the HRR effector subgroup and the intention-to-treat population^a^

Data are *n* (%) unless otherwise stated. ECOG, Eastern Cooperative Oncology Group. ^a^Percentages may not total 100 because of rounding. ^b^ECOG performance status ranges from 0 to 5, with higher scores reflecting greater disability. ^c^Scores on the Gleason scale range from 2 to 10, with higher scores indicating higher-grade cancer that may be more aggressive. ^d^All other co-occurring BRCA and non-BRCA alterations occurred at a frequency of less than 1%.

**Extended Data Table 2:**
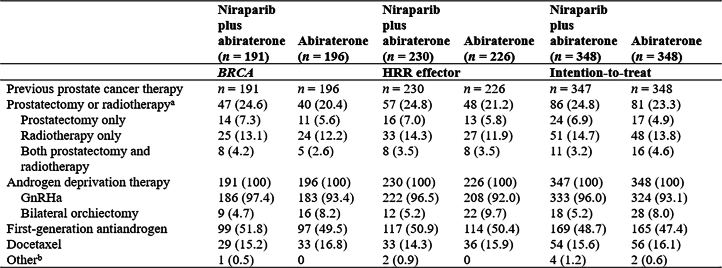
Prior prostate cancer therapy in the BRCA subgroup, the HRR effector subgroup and the intention-to-treat population

Data are *n* (%). GnRHa, gonadotropin-releasing hormone agonist/antagonist. ^a^Includes definitive radiotherapy as primary therapy and radiotherapy administered in the metastatic setting, as allowed in the study. ^b^Includes cyproterone and cyproterone acetate.

**Extended Data Table 3:**
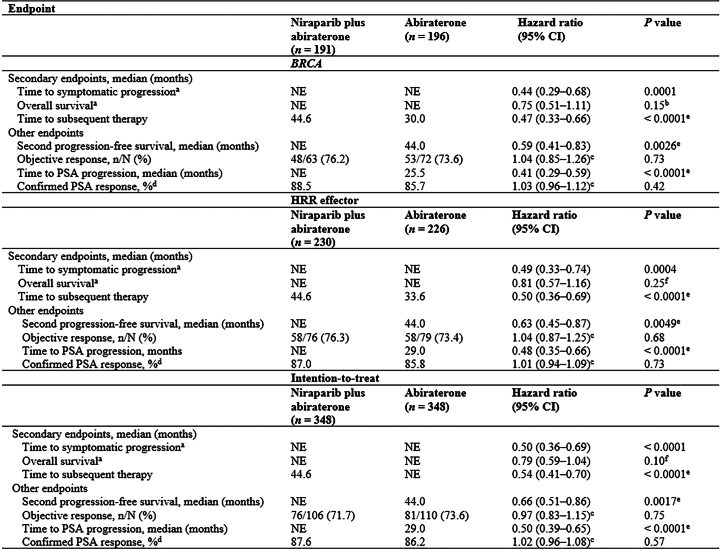
Prespecified secondary and other efficacy endpoints in the BRCA subgroup, the HRR effector subgroup and the intention-to-treat population. Comparison between treatment groups was performed using the stratified log-rank test. HRs and 95% CIs were estimated by stratified Cox proportional-hazards models

^a^Secondary endpoints included in graphical approach for testing key efficacy endpoints. ^b^First non-significant test in hierarchical graphical approach for testing key efficacy endpoints. *P* value provided for completeness. ^c^Value is relative risk. ^d^A PSA response was defined as a decrease of at least 50% from the baseline value. ^e^Nominal *P* value. ^f^Tests in graphical approach for testing key efficacy endpoints that followed a non-significant result. *P* value provided for completeness.

**Extended Data Table 4:**
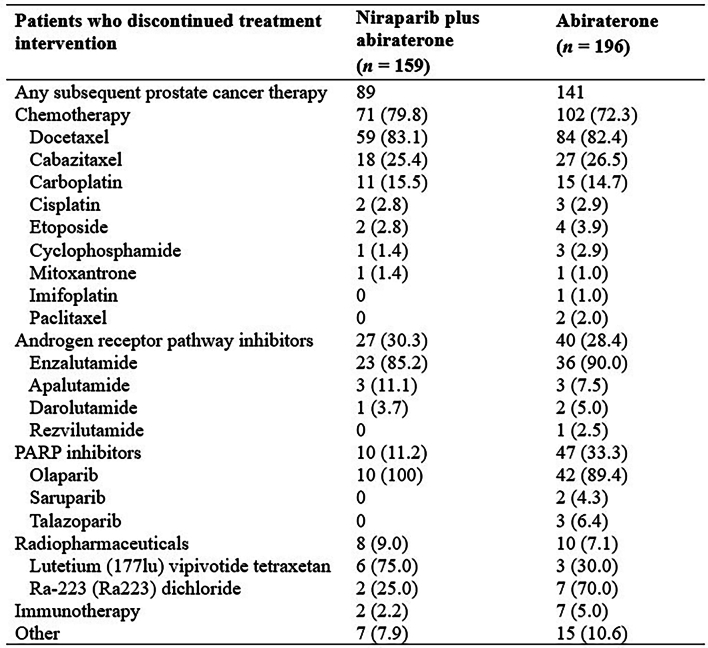
Subsequent prostate cancer therapy in the intention-to-treat population^a^

Data are *n* (%). Denominator for groups = Patients who discontinued treatment with any subsequent prostate cancer therapy. For individual treatments, denominator is the class (ie, docetaxel/chemotherapy). ^a^Data reflect all subsequent lines of therapy. Recurrent medications are counted only once per patient. Selected subsequent therapies are those with potential benefit in radiographic progression-free survival. Medications are coded using WHO Drug Dictionary Version 202409.

**Extended Data Table 5:**
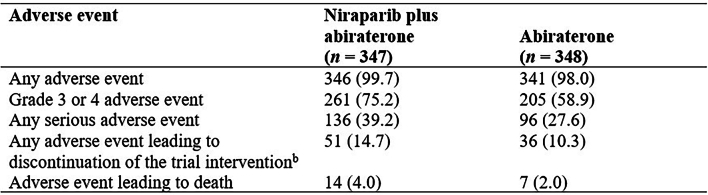
Summary of adverse events in the safety population^a^

Data are *n* (%). ^a^Shown are adverse events of any cause that occurred from the time of first dose of the trial intervention through 30 days after the last dose, according to preferred term and highest grade according to National Cancer Institute of Common Terminology Criteria for Adverse Events, version 5. ^b^An adverse event is counted as leading to a discontinuation of trial intervention if it leads to withdrawal of niraparib/placebo or abiraterone acetate/placebo or prednisone.

**Extended Data Table 6:**
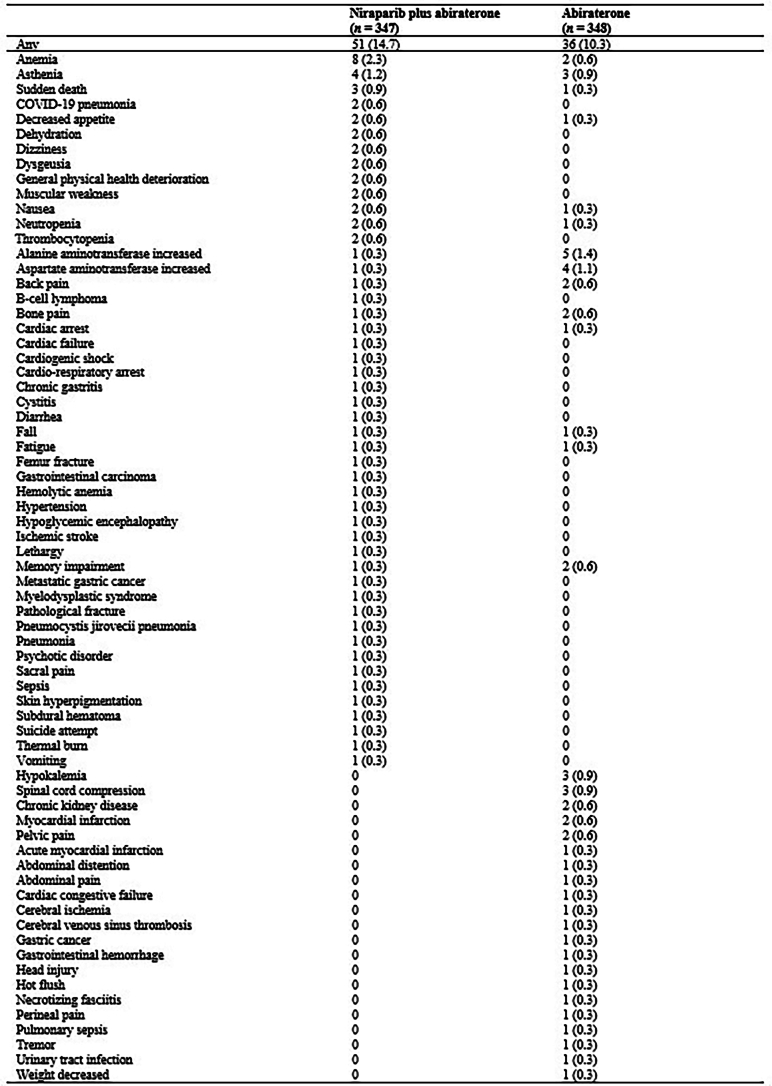
Adverse events leading to treatment discontinuation in the safety population^a^

Data are *n* (%). ^a^An adverse event is counted as leading to a discontinuation if it leads to withdrawal of niraparib/placebo or abiraterone acetate/placebo or prednisone.

**Extended Data Table 7:**
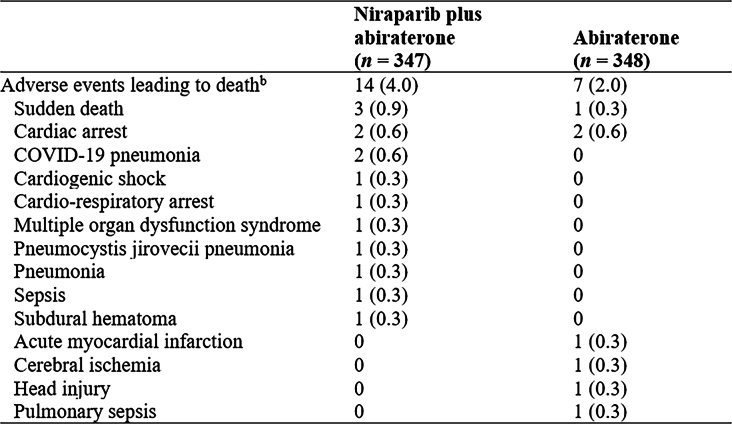
Causes of death in the safety population^a^

Data are *n* (%). ^a^Adverse events are coded using MedDRA version 27.1. ^b^Within 30 days of last dose of trial intervention.

## Data Availability

Janssen Pharmaceutical Companies of Johnson & Johnson’s data sharing policy is available at https://www.janssen.com/clinical-trials/transparency. As noted on this site, requests for study data access can be submitted through the Yale Open Data Access (YODA) project site at http://yoda.yale.edu. After completion of the study and finalization of any applicable regulatory review (for example, US Food and Drug Administration or European Medicines Agency decisions), YODA may provide access to deidentified participant-level data and clinical study reports as well as related documents (such as protocol and statistical analysis plan), upon approval of the request. Access is granted under strict data use agreements to qualified researchers for non-commercial scientific research purposes.
