# Supplementary material for: Niraparib and abiraterone acetate plus prednisone for HRR-deficient metastatic castration-sensitive prostate cancer: a randomized phase 3 trial
Source: Nat Med. 2025 Oct 7;31(12):4109–18. doi: 10.1038/s41591-025-03961-8 (PMC12705445; doi:10.1038/s41591-025-03961-8)
Supplement: Supplementary file 1 — AMPLITUDE study investigators, supplementary methods, Supplementary Fig. 1, Supplementary Table 1 and protocol [file 41591_2025_3961_MOESM1_ESM.pdf]

nature medicine

Supplementary information https://doi.org/10.1038/s41591-025-03961-8

Niraparib and abiraterone acetate plus prednisone for HRR-deficient metastatic castration-sensitive prostate cancer: a randomized phase 3 trial

In the format provided by the authors and unedited

AMPLITUDE study investigators

Principal Investigator	Country
Emilio Batagelj	Argentina
Sergio Bayo	Argentina
Ruben Bengio	Argentina
Omar Carranza	Argentina
Victor Chernobilsky	Argentina
Hernan Cutuli	Argentina
Susana Kahl	Argentina
Ernesto Korbenfeld	Argentina
Esteban Metrebian	Argentina
Monica Rondinon	Argentina
Jorge Salinas	Argentina
Ezequiel Slutsky	Argentina
Wei Chua	Australia
Timothy Clay	Australia
Thomas Ferguson	Australia
Howard Gurney	Australia
Anthony Joshua	Australia
Michelle Morris	Australia
Louise Nott	Australia
Francis Parnis	Australia
Annabel Smith	
Shahneen Sandhu	Australia
Aneta Suder	Australia
Yauheni Baranau	Belarus
Vasili Beliakouski	Belarus
Natalia Ivanova	Belarus
Yauheniya Karchmit	Belarus
Dmitry Noryk	Belarus
Sergey Polyakov	Belarus
Igor Sharf	Belarus
Marlies De Bock	Belgium
Jean-Charles Goeminne	Belgium
Geoffrey Matus	Belgium
Michiel Strijbos	Belgium
Vincent Verschaeve	Belgium
Nicolas Whenham	Belgium
Livia Andrade	Brazil
Márcio Costa	Brazil
Karina Costa Maia Vianna	Brazil
Eduardo Croenberger Costa e Silva	Brazil
Herbert Tavares Palmeira	
Andriano de Paula	Brazil

Mariane Dias	Brazil
Marcelo Freitas	Brazil
Andrea Juliana Gomes	Brazil
Gabriel Marques dos Anjos	Brazil
Ariel Kann	Brazil
Célio Kussumoto	Brazil
André Lima	Brazil
Suelen Martins	Brazil
José Maurício Mota	Brazil
Andre Murad	Brazil
Joao Antonio Junior Neif	Brazil
Renato Panhoca	Brazil
Helio Pinczowski	Brazil
Giovani Pioner	Brazil
Humberto Rodrigues Cottas	Brazil
Victor Santos	Brazil
Fabio Augusto Schutz	Brazil
Felipe José Silva Melo Cruz	Brazil
Brigitte Van Eyll	Brazil
Juliana Yorimi Yamaguchi	
Luciano Viana	Brazil
Velko Minchev	Bulgaria
Hristo Spasov	Bulgaria
Antoaneta Tomova	Bulgaria
Kim Chi	Canada
Urban Emmenegger	Canada
Daygen Finch	Canada
Neil Fleshner	Canada
Geoffrey Gotto	Canada
Michael Kolinsky	Canada
Sunil Parimi	Canada
Fred Saad	Canada
Bobby Shayegan	Canada
Francisco Vera-Badillo	Canada
Zhiwen Chen	China
Hongquian Guo	China
Yuhua Huang	China
Junhui Jiang	China
Lei Li	China
Hong Luo	China
Lulin Ma	China
Mingxing Qiu	China
Ting Sun	China
Zhongquan Sun	China
Ye Tian	China

Ben Wan	China
Shaogang Wang	China
Keji Xie	China
Boxin Xue	China
Dingwei Ye	China
Zhixian Yu	China
Hao Zeng	China
Dahong Zhang	China
Fangjian Zhou	China
Milos Brodak	Czech Republic
Otakar Čapoun	Czech Republic
Vladimir Samal	Czech Republic
Vladimir Student	Czech Republic
Gedske Daugaard	Denmark
Steinbjørn Hansen	Denmark
Mette Moe Kempel	Denmark
Redas Trepiakas	Denmark
Anne-Juel Christensen	
Christine Vestergaard Madsen	Denmark
Mathilde Cabart	France
Aurelien Carnot	France
Pierre Cornillon	France
Louis-Marie Dourthe	France
Sheik Emambux	France
Aude Flechon	France
Carole Helissey	France
Werner Hilgers	France
Sylvain Ladoire	France
Hakim Mahammedi	France
Raffaele Ratta	France
Friederike Schlurmann	France
Annic Schlurmann	
Diego Tosi	France
Antione Thiery Vuillemin	France
Martin Boegemann	Germany
Peter Hammerer	Germany
Axel Heidenreich	Germany
Eva Hellmis	Germany
Margitta Retz	Germany
Christian Thomas	Germany
Tilman Todenhöfer	Germany
Peter Arkosy	Hungary
Gergely Dombovari	Hungary
Judit Olah	Hungary
Istvan Sipocz	Hungary

Peter Tenke	Hungary
Andrea Uhlyarik	Hungary
Stephen Frank	Israel
Daniel Goldstein	Israel
Meital Levartovsky	Israel
Avivit Peer	Israel
Keren Roubinov	Israel
David Sarid	Israel
Nicole Brighi	Italy
Ugo De Giorgi	
Consuelo Buttigliero	Italy
Orazio Caffo	Italy
Gaetano Facchini	Italy
Luca Galli	Italy
Alketa Hamzaj	Italy
Francesca La Russa	Italy
Evaristo Maiello	Italy
Marco Maruzzo	Italy
Alessandra Mosca	Italy
Andrea Necchi	Italy
Franco Nolè	Italy
Bruno Perrucci	Italy
Sandro Pignata	Italy
Giuseppe Procopio	Italy
Elena Verzoni	
Hadi Bin Ab. Jalil	Malaysia
Chin Heng Fong	Malaysia
Chun Sen Lim	Malaysia
Vickee Rajeswaran	
Marniza Saad	Malaysia
Prathepamalar Yehgambaram	Malaysia
David Calvo	Mexico
Yamil Lopez Chuken	Mexico
Gabriela Regalado	Mexico
Roberto Romero	Mexico
Julia Saenz	Mexico
Laurens Beerepoot	Netherlands
Rik Somford	Netherlands
Addy van de Luijtgaarden	Netherlands
Pieter van den Berg	Netherlands
Shaun Costello	New Zealand
Simon Fu	New Zealand
Dorota Filarska	Poland
Izabela Glanowska	Poland
Boguslawa Karaszewska	Poland

Iwona Skoneczna	Poland
Krzysztof Tupikowski	Poland
Marcin Zaremba	Poland
Bogdan Zurawski	Poland
Nuno Vau	Portugal
Seok-Soo Byun	Republic of Korea
YoungDeuk Choi	Republic of Korea
SeolHo Choo	Republic of Korea
Hongkoo Ha	Republic of Korea
JaeYoung Joung	Republic of Korea
Take Won Kang	Republic of Korea
Choung-Soo Kim	Republic of Korea
Bumjin Lim	
Chun Il Kim	Republic of Korea
Wonho Jung	
Cheol Kwak	Republic of Korea
Tae Gyun Kwon	Republic of Korea
Ji Youl Lee	Republic of Korea
Jae Sung Lim	Republic of Korea
Seong Il Seo	Republic of Korea
Boris Alekseev	Russia
Vagif Atduev	Russia
Denis Khvorostenko	Russia
Gennady Kolesnikov	Russia
Alexander Bystrov	
Evgeny Kopyltsov	Russia
Alexander Lykov	Russia
Konstantin Penkov	Russia
Andrey Semenov	Russia
Pavel Skopin	Russia
Marat Urmantsev	Russia
Evgeny Usynin	Russia
Vladimir Vladimirov	Russia
Richard Khanyile	South Africa
Thamsanqa Madlala	South Africa
Marcos Aller	Spain
Teresa Alonso	Spain
Elena Castro	Spain
Alvaro Montesa	
Lucia Oliva	
Mario Dominguez	Spain
Claudia Fina	Spain
Enrique Gallardo	Spain
Álvaro Juárez	Spain
Jose Pablo Maroto	Spain

Jose Maria Martinez	Spain
Rafael Antonio Medina	Spain
Maria Jose Ribal	Spain
Miguel Rodrigo	Spain
Alfredo Rodríguez Antolín	Spain
Javier Romero	Spain
Nuria Romero Laorden	Spain
Enrique Castellanos	Sweden
Chunde Li	Sweden
Chao-Yuan Huang	Taiwan
Yi-Hsiu Huang	Taiwan
Yen-Chuan Ou	Taiwan
Yu-Li Su	Taiwan
His-Chin Wu	Taiwan
Wen-Jeng Wu	Taiwan
Kai-Jie Yu	Taiwan
Phichai Chansriwong	Thailand
Pongwut Danchaivijitr	Thailand
Ozturk Ates	Turkey
Umut Demirci	Turkey
Meltem Ekenel	Turkey
Ìlhan Hacibekiroglu	Turkey
Fatih Kose	Turkey
Huseyin Salih Semiz	Turkey
Birol Ocak	Turkey
Mustafa Özgüroğlu	Turkey
Hakan Ozturk	Turkey
Mehmet Ali Nahit Sendur	Turkey
Deniz Tural	Turkey
Mesut Yilmaz	
Yuksel Urun	Turkey
Ozan Yazici	Turkey
Iaroslav Gotsuliak	Ukraine
Gennadii Khareba	Ukraine
Oleksandr Kostiuk	Ukraine
Oksana Lipetska	Ukraine
Talon Nalbandian	Ukraine
Yana Kidik	
Viktor Paramonov	Ukraine
Anna Pidverbetska	Ukraine
Valerii Sakalo	Ukraine
Eduard Stakhovsky	Ukraine
Viktor Stus	Ukraine
Nataliia Zvonarova	Ukraine
Gerhardt Attard	United Kingdom

Amarnath Challapalli	United Kingdom
Peter Hoskin	United Kingdom
Pasquale Innominato	United Kingdom
Debra Josephs	United Kingdom
Neil McPhail	United Kingdom
Omi Parikh	United Kingdom
Alison Reid	United Kingdom
Angus Robinson	United Kingdom
Mark Tuthill	United Kingdom
Duncan Wheatley	United Kingdom
Madalina Chifu	
Rony Abou-Jawde	United States of America
Neeraj Agarwal	United States of America
Leonard Appleman	United States of America
Georges Azzi	United States of America
Kethandapatti Balaji	United States of America
Alan Bryce	United States of America
David Cahn	United States of America
Heather Cheng	United States of America
Franklin Chu	United States of America
Eugene Cone	United States of America
Curtiss Dunshee	United States of America
Karyn Dyehouse	United States of America
Mark Fleming	United States of America
Benjamin Gartrell	United States of America
Julie Graff	United States of America
Jason Hafron	United States of America
Joelle Hamilton	United States of America
Austin Lutz	
Lawrence Karsh	United States of America
Eric Knoche	United States of America
Charles Kurkul	United States of America
Michael Lattanzi	United States of America
Matthew Logsdon	United States of America
Janelle Meyer	United States of America
David Morris	United States of America
Vivek Narayan	United States of America
Asit Paul	United States of America
Christopher Pieczonka	United States of America
Dana Rathkopf	United States of America
Rahul Ravilla	United States of America
Donald Richards	United States of America
Stephen Savage	United States of America
Paul Sieber	United States of America
Dennis Slater	United States of America

Selena Stuart	United States of America
Deepnarayan Tiwarri	United States of America
Che-Kai Tsao	United States of America
Ronald Tutrone	United States of America
Hema Vankayala	United States of America

Note: Some sites had different primary investigators over the course of the trial.

Supplementary methods

Additional eligibility requirements

- Patients who started a gonadotropin-releasing hormone agonist ≤28 days prior to randomization were required to receive a first-generation antiandrogen for ≥14 days prior to randomization with discontinuation prior to randomization
- Patients who received prior docetaxel must have met the following criteria:
  - Received a maximum of six cycles of docetaxel therapy for metastatic castration-sensitive prostate cancer
  - Received the last dose of docetaxel ≤3 months prior to randomization
  - Maintained a response to docetaxel of stable disease or better, by investigator assessment of imaging and/or prostate-specific antigen (PSA), prior to randomization
- Other allowed therapy for metastatic castration-sensitive prostate cancer:
  - Maximum of one course of radiation and one surgical intervention for symptomatic control of prostate cancer (eg, uncontrolled pain, impending spinal cord compression, or obstructive symptoms). Patients with radiation or surgical interventions to all known sites of metastatic disease were excluded. Radiation had to be completed prior to randomization
  - Up to a maximum of 6 months of androgen deprivation therapy prior to randomization
  - Up to a maximum of 45 days of abiraterone plus prednisone prior to randomization

    - Up to a maximum of 2 weeks of ketoconazole for prostate cancer prior to randomization
    - Prior androgen receptor–targeted therapy (eg, apalutamide, darolutamide, enzalutamide), immunotherapy or radiopharmaceutical agents for prostate cancer were excluded, with the exceptions noted above
  - Allowed prior treatments for localized prostate cancer included radical prostatectomy (with or without lymph node dissection), radiation therapy and other locally directed treatments to the prostate per institutional standards of care
    - Patients who received androgen deprivation therapy or first-generation antiandrogens must have had ≤3 years total and completed ≥1 year prior to randomization

Definitions of secondary endpoints

- Overall survival was defined as the time from randomization to death from any cause. Patients alive at the time of analysis were censored on the last date the patient was known to be alive
- Time to symptomatic progression was defined as time from randomization to any of the following (whichever occurred first):
  - Use of external beam radiation for skeletal or pelvic symptoms (only radiation planned prior to randomization will not be considered as symptomatic progression)
  - Need for tumor-related orthopedic surgical intervention
  - Other cancer-related procedures (eg, nephrostomy insertion, bladder catheter insertion or surgery for tumor symptoms)

  - Cancer-related morbid events (ie, fracture [symptomatic and/or pathologic], cord compression, urinary obstructive events)
  - Initiation of a new systemic anticancer therapy because of cancer symptoms
- Time to subsequent therapy, defined as time from randomization to the initiation of subsequent therapy for prostate cancer

Definitions of other endpoints

- Time to second progression-free survival was defined as time from randomization to first occurrence of disease progression (radiographic, clinical or PSA progression) on first subsequent therapy for prostate cancer or death, whichever occurred first
- Objective response was defined as achieving a partial response or complete response according to modified Response Evaluation Criteria In Solid Tumors v1.1
- Duration of response in patients with measurable disease at baseline (based on modified Response Evaluation Criteria In Solid Tumors v1.1) was defined as time from documented response to the first date of documented disease progression.
- Time to PSA progression was defined as time from randomization to PSA progression based on Prostate Cancer Working Group 3 criteria
- Objective response, radiographic progression-free survival and PSA response across individual and subgroups of deleterious germline or somatic HRR gene alterations
- Time to deterioration was reported for Functional Assessment of Cancer Therapy-Prostate (FACT-P) total and all subscales

Definition of bone scan progression

- Patients whose week 8 scan was observed to have two or more new bone lesions with either of the following would be defined as having bone progression:

  - A confirmatory scan, performed ≥6 weeks later, showing two or more new lesions compared with the week 8 scan (ie, a total of at least four new lesions compared with the baseline scan) was considered to indicate bone scan progression at week 8
  - A confirmatory scan that did not show two or more new lesions compared with the week 8 scan was not considered to indicate bone scan progression. The first scan that showed progression compared with the week 8 scan was considered the bone progression time point if these lesions were confirmed by a subsequent scan ≥6 weeks later
- For patients whose week 8 scan did not have two or more new bone lesions compared with the baseline scan, the first scan time point that showed two or more new lesions compared with the week 8 scan was considered the bone scan progression time point if confirmed by a subsequent scan ≥6 weeks later

Patient-reported outcome scales

FACT-P includes a general function status scale (consisting of four subscales: physical wellbeing, social and family wellbeing, emotional wellbeing and functional wellbeing) and a prostate cancer–specific subscale (ranging from 0 to 156, with higher scores indicating better functional status).

Supplementary Fig. 1 | Graphical approach for testing key efficacy endpoints[a]

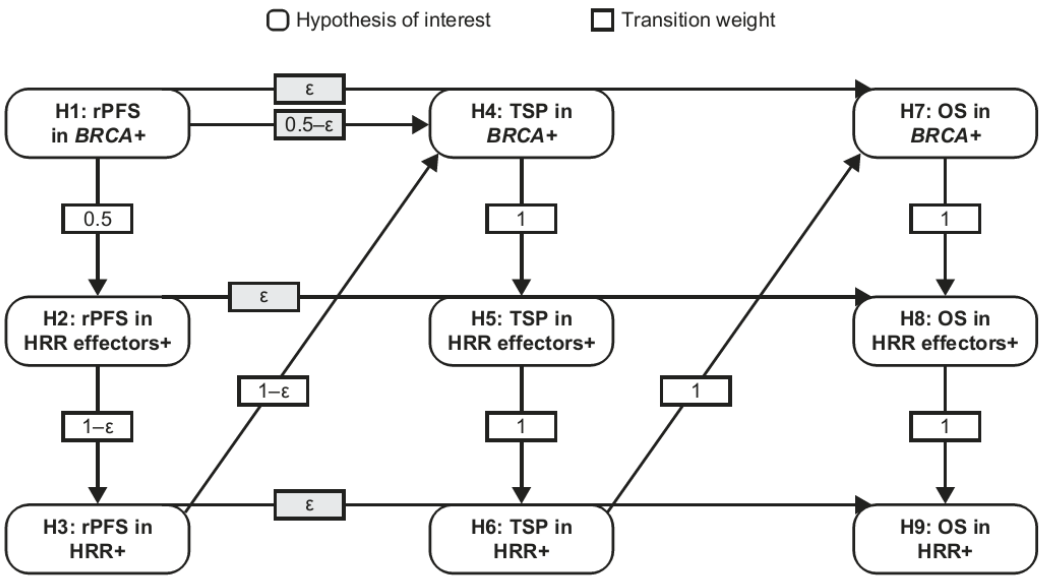

The hypothesis testing starts with radiographic progression-free survival at a two-sided alpha equal to 0.05. Per testing hierarchy, if statistical significance is met in the *BRCA* subgroup, then half of the alpha (0.025) will be passed to radiographic progression-free survival in the HRR effector subgroup and the other half split between time to symptomatic progression and overall survival in the *BRCA* subgroup. If statistical significance is met in the HRR effector subgroup, then the alpha (0.025) will be split between radiographic progression-free survival analysis in all patients and overall survival in the HRR effector subgroup (the full planned graphical procedure is found in the statistical analysis plan provided in this supplementary appendix). HRR, homologous recombination repair; OS, overall survival; rPFS, radiographic progression-free survival; TSP, time to symptomatic progression. [a]ε is set to 0.01.

Supplementary Table 1 | Adverse events leading to (a) dose reduction[a] and (b) dose interruption[b] in the safety population

a

	**Niraparib plus abiraterone (*n* = 347)**	**Abiraterone (*n* = 348)**
Any	76 (21.9)	24 (6.9)
Anemia	47 (13.5)	1 (0.3)
Neutropenia	4 (1.2)	1 (0.3)
Dizziness	3 (0.9)	0
Hypertension	3 (0.9)	3 (0.9)
Leukopenia	3 (0.9)	0
Thrombocytopenia	3 (0.9)	0
Asthenia	2 (0.6)	1 (0.3)
Cardiac failure	2 (0.6)	0
Dyspnea	2 (0.6)	0
Fatigue	2 (0.6)	1 (0.3)
Hypokalemia	2 (0.6)	2 (0.6)
Nausea	2 (0.6)	0
Peripheral edema	2 (0.6)	0
Adrenal suppression	1 (0.3)	0
Alanine aminotransferase increased	1 (0.3)	8 (2.3)
Chest discomfort	1 (0.3)	0
Constipation	1 (0.3)	0
Contusion	1 (0.3)	0
Depression	1 (0.3)	0
Dysgeusia	1 (0.3)	0
Dyspepsia	1 (0.3)	0
Dysphagia	1 (0.3)	0
Gamma-glutamyltransferase increased	1 (0.3)	0
Gastroesophageal reflux disease	1 (0.3)	0
Hyperglycemia	1 (0.3)	0
Insomnia	1 (0.3)	1 (0.3)
Lethargy	1 (0.3)	0
Lymphopenia	1 (0.3)	0
Mania	1 (0.3)	0
Renal failure	1 (0.3)	0
Weight decreased	1 (0.3)	0
Aspartate aminotransferase increased	0	3 (0.9)
Abdominal pain	0	1 (0.3)
Chronic kidney disease	0	1 (0.3)
Diarrhea	0	1 (0.3)
Malaise	0	1 (0.3)
Palpitations	0	1 (0.3)

	Niraparib plus abiraterone (*n* = 347)	Abiraterone (*n* = 348)
Urinary retention	0	1 (0.3)
Weight increased	0	1 (0.3)

b

	Niraparib plus abiraterone (*n* = 347)	Abiraterone (*n* = 348)
Any	232 (66.9)	147 (42.4)
Anemia	95 (27.4)	8 (2.3)
Neutropenia	36 (10.4)	7 (2.0)
Hypertension	30 (8.6)	18 (5.2)
COVID-19	29 (8.4)	34 (9.8)
Thrombocytopenia	29 (8.4)	1 (0.3)
Hypokalemia	28 (8.1)	26 (7.5)
Leukopenia	14 (4.0)	0
Vomiting	13 (3.7)	6 (1.7)
Nausea	11 (3.2)	3 (0.9)
Urinary tract infection	11 (3.2)	5 (1.4)
Asthenia	9 (2.6)	2 (0.6)
Fatigue	9 (2.6)	2 (0.6)
Pneumonia	9 (2.6)	7 (2.0)
SARS-CoV-2 test positive	9 (2.6)	5 (1.4)
Alanine aminotransferase increased	6 (1.7)	19 (5.5)
Diarrhea	6 (1.7)	5 (1.4)
Blood creatinine increased	5 (1.4)	0
Cardiac failure	5 (1.4)	2 (0.6)
Dyspnea	5 (1.4)	1 (0.3)
Lymphopenia	5 (1.4)	2 (0.6)
Aspartate aminotransferase increased	4 (1.2)	16 (4.6)
Dizziness	4 (1.2)	4 (1.1)
Sepsis	4 (1.2)	4 (1.1)
Sinus tachycardia	4 (1.2)	0
Abdominal pain	3 (0.9)	1 (0.3)
Acute kidney injury	3 (0.9)	2 (0.6)
Congestive cardiac failure	3 (0.9)	1 (0.3)
Constipation	3 (0.9)	0
Peripheral oedema	3 (0.9)	2 (0.6)
Upper respiratory tract infection	3 (0.9)	1 (0.3)
Weight decreased	3 (0.9)	0
Abdominal pain upper	2 (0.6)	0
Acute cardiac failure	2 (0.6)	0
Appendicitis	2 (0.6)	0
Atrial fibrillation	2 (0.6)	2 (0.6)

	Niraparib plus abiraterone (*n* = 347)	Abiraterone (*n* = 348)
Bronchitis	2 (0.6)	1 (0.3)
Decreased appetite	2 (0.6)	0
Dehydration	2 (0.6)	0
Dysgeusia	2 (0.6)	0
Fall	2 (0.6)	0
Headache	2 (0.6)	1 (0.3)
Influenza	2 (0.6)	1 (0.3)
Musculoskeletal pain	2 (0.6)	0
Pleural effusion	2 (0.6)	0
Pyrexia	2 (0.6)	5 (1.4)
Stomatitis	2 (0.6)	0
Urinary retention	2 (0.6)	0
Urosepsis	2 (0.6)	3 (0.9)
Abdominal discomfort	1 (0.3)	0
Acute coronary syndrome	1 (0.3)	0
Alpha hemolytic streptococcal infection	1 (0.3)	0
Anal inflammation	1 (0.3)	0
Angina pectoris	1 (0.3)	1 (0.3)
Angina unstable	1 (0.3)	1 (0.3)
Aphasia	1 (0.3)	0
Arthralgia	1 (0.3)	2 (0.6)
Back pain	1 (0.3)	3 (0.9)
Biliary obstruction	1 (0.3)	0
Bladder injury	1 (0.3)	0
Blood thyroid hormone–stimulating hormone increased	1 (0.3)	0
Carotid arteriosclerosis	1 (0.3)	0
Cerebral artery occlusion	1 (0.3)	0
Cellulitis	1 (0.3)	0
Chills	1 (0.3)	0
Cholelithiasis	1 (0.3)	0
Chronic kidney disease	1 (0.3)	2 (0.6)
Chronic obstructive pulmonary disease	1 (0.3)	1 (0.3)
Circulatory collapse	1 (0.3)	0
Clostridium difficile infection	1 (0.3)	0
Coronary artery disease	1 (0.3)	0
Cough	1 (0.3)	1 (0.3)
COVID-19 pneumonia	1 (0.3)	2 (0.6)
Deep vein thrombosis	1 (0.3)	0
Delirium	1 (0.3)	0
Depression	1 (0.3)	2 (0.6)
Disseminated intravascular coagulation	1 (0.3)	0
Dysarthria	1 (0.3)	0

	**Niraparib plus abiraterone (*n* = 347)**	**Abiraterone (*n* = 348)**
Dyspepsia	1 (0.3)	0
Dysphagia	1 (0.3)	0
Edema	1 (0.3)	0
Esophageal varices	1 (0.3)	0
Electrocardiogram T wave inversion	1 (0.3)	0
Emphysema	1 (0.3)	0
Enterocolitis	1 (0.3)	0
Femoral neck fracture	1 (0.3)	0
Femur fracture	1 (0.3)	0
Gait disturbance	1 (0.3)	0
Gastric adenocarcinoma	1 (0.3)	0
Gastric stenosis	1 (0.3)	0
Gastric ulcer	1 (0.3)	1 (0.3)
Gastroenteritis	1 (0.3)	0
Gastroenteritis norovirus	1 (0.3)	0
Gastroenteritis salmonella	1 (0.3)	0
Gastrointestinal hemorrhage	1 (0.3)	0
Gastrointestinal pain	1 (0.3)	0
Gingival pain	1 (0.3)	0
Gout	1 (0.3)	0
Hand fracture	1 (0.3)	0
Hip fracture	1 (0.3)	0
Helicobacter infection	1 (0.3)	0
Hematuria	1 (0.3)	1 (0.3)
Hemolytic anemia	1 (0.3)	0
Hemorrhagic stroke	1 (0.3)	0
HIV infection	1 (0.3)	0
Hypercalcemia	1 (0.3)	0
Hyperkalemia	1 (0.3)	0
Hyperlipidemia	1 (0.3)	0
Hypernatremia	1 (0.3)	0
Hypoglycemia	1 (0.3)	1 (0.3)
Hypophosphatemia	1 (0.3)	0
Hypotension	1 (0.3)	0
Hypoxia	1 (0.3)	0
Impaired gastric emptying	1 (0.3)	0
Influenza like illness	1 (0.3)	1 (0.3)
Inguinal hernia	1 (0.3)	0
International normalized ratio increased	1 (0.3)	0
Ischemic stroke	1 (0.3)	3 (0.9)
Ketoacidosis	1 (0.3)	0
Lethargy	1 (0.3)	0
Ligament strain	1 (0.3)	0

	Niraparib plus abiraterone (*n* = 347)	Abiraterone (*n* = 348)
Lower urinary tract symptoms	1 (0.3)	0
Malnutrition	1 (0.3)	0
Mania	1 (0.3)	0
Memory impairment	1 (0.3)	0
Meningioma	1 (0.3)	0
Mouth ulceration	1 (0.3)	0
Muscular weakness	1 (0.3)	0
Myocardial infarction	1 (0.3)	0
Nephrolithiasis	1 (0.3)	0
Nervous system disorder	1 (0.3)	0
Noncardiac chest pain	1 (0.3)	0
Oropharyngeal pain	1 (0.3)	0
Orthostatic hypotension	1 (0.3)	0
Palpitations	1 (0.3)	1 (0.3)
Papillary cystadenoma lymphomatosum	1 (0.3)	0
Pathological fracture	1 (0.3)	2 (0.6)
Pericardial effusion	1 (0.3)	0
Pneumonia, influenzal	1 (0.3)	0
Pneumocystis jirovecii pneumonia	1 (0.3)	0
Polymyalgia rheumatica	1 (0.3)	0
Presyncope	1 (0.3)	0
Pulmonary oedema	1 (0.3)	0
Pyelonephritis	1 (0.3)	0
Rash	1 (0.3)	0
Renal cell carcinoma	1 (0.3)	0
Renal failure	1 (0.3)	0
Respiratory failure	1 (0.3)	1 (0.3)
Skin laceration	1 (0.3)	0
Subdural hematoma	1 (0.3)	0
Subdural hemorrhage	1 (0.3)	0
Transitional cell carcinoma	1 (0.3)	0
Traumatic hematoma	1 (0.3)	0
Urethral stenosis	1 (0.3)	0
Ventricular tachycardia	1 (0.3)	0
Vertigo	1 (0.3)	0
Viral infection	1 (0.3)	0
Vision blurred	1 (0.3)	0
Blood alkaline phosphatase increased	0	3 (0.9)
Hyperglycemia	0	3 (0.9)
Intestinal obstruction	0	3 (0.9)
Acute myocardial infarction	0	2 (0.6)
Gamma-glutamyltransferase increased	0	2 (0.6)
Hyperbilirubinemia	0	2 (0.6)

	Niraparib plus abiraterone (*n* = 347)	Abiraterone (*n* = 348)
Insomnia	0	2 (0.6)
Nasopharyngitis	0	2 (0.6)
Pain in extremity	0	2 (0.6)
Tremor	0	2 (0.6)
Agitation	0	1 (0.3)
Anxiety	0	1 (0.3)
Blindness	0	1 (0.3)
Conjunctivitis allergic	0	1 (0.3)
Coronary artery stenosis	0	1 (0.3)
Decubitus ulcer	0	1 (0.3)
Dermatitis	0	1 (0.3)
Disorientation	0	1 (0.3)
Dysphemia	0	1 (0.3)
Encephalopathy	0	1 (0.3)
Face edema	0	1 (0.3)
Food poisoning	0	1 (0.3)
Gastric hemorrhage	0	1 (0.3)
Gastrointestinal infection	0	1 (0.3)
General physical health deterioration	0	1 (0.3)
Hallucination, visual	0	1 (0.3)
Head injury	0	1 (0.3)
Hernia pain	0	1 (0.3)
Hot flush	0	1 (0.3)
Hypertriglyceridemia	0	1 (0.3)
Ileus	0	1 (0.3)
Joint swelling	0	1 (0.3)
Larynx irritation	0	1 (0.3)
Mood altered	0	1 (0.3)
Musculoskeletal chest pain	0	1 (0.3)
Myocarditis	0	1 (0.3)
Parkinsonism	0	1 (0.3)
Periodontitis	0	1 (0.3)
Peripheral swelling	0	1 (0.3)
Periprosthetic fracture	0	1 (0.3)
Pneumonia, mycoplasmal	0	1 (0.3)
Proctalgia	0	1 (0.3)
Pulmonary sepsis	0	1 (0.3)
Respiratory tract infection	0	1 (0.3)
Sciatica	0	1 (0.3)
Small intestinal obstruction	0	1 (0.3)
Suicidal ideation	0	1 (0.3)
Swelling face	0	1 (0.3)
Tachypnea	0	1 (0.3)

	**Niraparib plus abiraterone (*n* = 347)**	**Abiraterone (*n* = 348)**
Tibia fracture	0	1 (0.3)

Data are n (%). [a]An adverse event is counted as a dose reduction, if it leads to reduction of niraparib/placebo or abiraterone acetate/placebo or prednisone. [b]An adverse event is counted as leading to a dose interruption if it leads to interruption of niraparib/placebo or abiraterone acetate/placebo or prednisone.

Janssen Research & Development *

Clinical Protocol

A Phase 3 Randomized, Placebo-controlled, Double-blind Study of Niraparib in Combination with Abiraterone Acetate and Prednisone Versus Abiraterone Acetate and Prednisone for the Treatment of Participants with Deleterious Germline or Somatic Homologous Recombination Repair (HRR) Gene-Mutated Metastatic Castration-Sensitive Prostate Cancer (mCSPC)

AMPLITUDE

Protocol 67652000PCR3002; Phase 3
AMENDMENT 4

CJNJ-67652000 (niraparib/abiraterone acetate fixed dose combination)

*Janssen Research & Development is a global organization that operates through different legal entities in various countries/territories. Therefore, the legal entity acting as the sponsor for Janssen Research & Development studies may vary, such as, but not limited to Janssen Biotech, Inc.; Janssen Products, LP; Janssen Biologics, BV; Janssen-Cilag International NV; Janssen, Inc; Janssen Pharmaceutica NV; Janssen Sciences Ireland UC; Janssen Biopharma Inc.; or Janssen Research & Development, LLC. The term "sponsor" is used throughout the protocol to represent these various legal entities; the sponsor is identified on the Contact Information page that accompanies the protocol.

United States (US) sites of this study will be conducted under US Food & Drug Administration Investigational New Drug (IND) regulations (21 CFR Part 312).

IND: 131190
EudraCT NUMBER: 2020-002209-25
EU Trial Number: 2023-506365-64

Status: Approved
Date: 28 August 2023
Prepared by: Janssen Research & Development, LLC
EDMS number: EDMS-ERI-207490517, 8.0

GCP Compliance: This study will be conducted in compliance with Good Clinical Practice, and applicable regulatory requirements.

Confidentiality Statement
The information provided herein contains Company trade secrets, commercial or financial information that the Company customarily holds close and treats as confidential. The information is being provided under the assurance that the recipient will maintain the confidentiality of the information under applicable statutes, regulations, rules, protective orders or otherwise.

PROTOCOL AMENDMENT SUMMARY OF CHANGES TABLE

DOCUMENT HISTORY	
Amendment 4	28 August 2023
Amendment 3	09 February 2023
Amendment 2	25 October 2021
Amendment 1	21 December 2020
Original Protocol	10 June 2020

Amendment 4 (28 August 2023)

Overall Rationale for the Amendment: The overall rationale for this amendment is to modify the secondary efficacy endpoint from "Symptomatic progression free survival" to "Time to symptomatic progression". In addition, this amendment consolidates the protocol for European Clinical Trial Regulation (EU CTR) transition.

The changes made to the clinical protocol 67652000PCR3002 as part of Protocol Amendment 4 are listed below, including the rationale of each change and a list of all applicable sections. Changes made in previous protocol amendments are listed in Section 10.12 Appendix 12: Protocol Amendment History.

Section number and Name	Description of Change	Brief Rationale
Title page	Added IND and EU Trial numbers.	To comply with EU CTR requirements.
1.1. Synopsis	Added reference to protocol version and date, including IND, Eudra CT, and EU Trial numbers.	To comply with EU CTR requirements.
1.1. Synopsis	Added a Benefit-risk Assessment subsection.	To comply with EU CTR requirements.
1.1. Synopsis	Deleted text: "All participants will receive ADT (ie, gonadotropin-releasing hormone analogue or surgical castration)."	Removed as this is not included as study treatment.
1.3. Schedule of Activities (SoA), Table 2	New Footnote "a" added to title of Table 2: "The Open-label Extension Phase and/or Long-term Extension Phase will begin upon notification from the Sponsor. At that time, the applicable procedures should be followed, as detailed in Appendix 10: Open-label Extension Phase and Appendix 11: Long-term Extension Phase."<br>Subsequent footnote order modified.	To clarify details of the OLE and LTE Phases.
1.3. Schedule of Activities (SoA), Table 2	Modified language for CT or MRI Note: "Until radiographic progression. See Section 8.1.1 for details."	To encourage the submission of imaging in trial follow-up phase.
1.3. Schedule of Activities (SoA), Table 2	Survival status Footnote "c" added: "May be obtained at a shorter interval than every 4 months during follow-up if required for database lock."	To clarify that more frequent follow-up is permitted.
3. Objectives and Endpoints; 9.4.3. Secondary Endpoints	Modify the secondary efficacy endpoint from "Symptomatic progression free survival" to "Time to symptomatic progression".	To capture a patient-centered endpoint. As death for any cause is captured in radiographic progression-free survival (rPFS) and overall survival, it was removed from the endpoint related to symptomatic progression.

Section number and Name	Description of Change	Brief Rationale
		No changes were made to the corresponding objective.
3. Objectives and Endpoints; 9.4.4. Other Endpoints	"Overall response" to "Objective response".	To align protocol with Response Evaluation Criteria in Solid Tumors (RECIST) terminology.
3. Objectives and Endpoints; 9.4.3 Secondary Endpoints	Footnote "b": "Death from any cause" removed from definition of endpoint.	Updated to correspond to the secondary endpoint modification.
4.4. End of Study Definition	Language added to include the Open-label Extension (OLE) and Long-term Extension (LTE) Phases as part of End of Study Definition.	Definition updated to align with EU regulations.
5.4. Screen Failures	Text added to specify whether the investigator will use interactive web response system (IWRS) to directly generate screening and enrollment logs.	IWRS language added to align with sponsor template requirement.
6.1. Study Medication(s) Administered	Table added providing designations of the study medicinal products (investigational medicinal product [IMP], non-IMP/Auxiliary Medicinal Product [AxMP]) to clarify medicinal product designations and conform with the definitions under EU CTR.	To comply with EU CTR regulations.
6.1.2. Study Medication, Table 6	Niraparib dosage formulation updated to include "or tablet".<br>Added footnote: "*Niraparib tablet may only be used during the Open-Label Extension/Long-Term Extension Phase after regulatory approval in the jurisdiction as applicable."	To align with available supply.
6.5. Dose Modification, Table 9	Sub-bullet in Hematological Toxicities notes section changed from "or" to "and".	Minor edit to improve clarity.
6.8. Concomitant Therapy	The text "study medication" was removed.	Text removed to include only relevant items in this section.
8.1.1. Evaluations for Primary Efficacy Assessment	Language added: "Sites are encouraged to continue submitting scans until radiographic progression is confirmed by both the investigator and blinded independent central review."	To encourage the submission of imaging in trial follow-up phase.
8.3.4. Regulatory Reporting Requirements for Serious Adverse Events and Anticipated Events	First sentence modified to include mandatory safety reporting text.	To align with sponsor template requirement.
8.6. Biomarkers	Language added: "Sample collection and testing will comply with local regulations."	To align with sponsor template requirement.
9.4.6 Other Analyses	Text deleted: "For each treatment group,"	Text deleted to improve clarity.
10.1. Appendix 1: Abbreviations	The list of abbreviations was updated.	The list is updated to include new terms and abbreviations used in revised text.
10.2. Appendix 2: Regulatory, Ethical, and Study Oversight Considerations	Under Subheading "Informed Consent Process", modification was made to the preference to allow virtual reconsent.	To clarify where virtual reconsent is allowed.
10.2. Appendix 2: Regulatory, Ethical, and	New subsection added, "Recruitment Strategy".	To comply with EU CTR regulations and sponsor template requirements.

Section number and Name	Description of Change	Brief Rationale
Study Oversight Considerations		
10.2. Appendix 2: Regulatory, Ethical, and Study Oversight Considerations	Updated the section on data protection to comply with EU CTR requirements and sponsor template.	To clarify rules on protection of personal data to comply with EU CTR regulations and sponsor template.
10.5. Appendix 5: Guidance on Study Conduct During the COVID-19 Pandemic	Full references updated to align with reference standard in the protocol.	To align with the sponsor template requirement.
10.10. Appendix 10: Open-label Extension Phase	Text rewritten to clarify the details of the OLE Phase.	Text updated to clarify procedures during the OLE Phase.
10.11. Appendix 11: Long-term Extension Phase	Language added to clarify the end of participation in the LTE Phase.	To clarify details of the LTE Phase.
10.12. Appendix 12: Protocol Amendment History	The Protocol Amendment 3 Summary of Changes table was moved to Appendix 12.	To align with the sponsor template requirement.
Throughout the protocol	Minor grammatical, formatting, or spelling changes were made.	Minor errors were noted.

TABLE OF CONTENTS

PROTOCOL AMENDMENT SUMMARY OF CHANGES TABLE 2

TABLE OF CONTENTS 5

LIST OF IN-TEXT TABLES AND FIGURES 8

1. PROTOCOL SUMMARY 9
1.1. Synopsis 9
1.2. Schema 12
1.3. Schedule of Activities (SoA) 13

2. INTRODUCTION 20
2.1. Study Rationale 21
2.1.1. Background on mCSPC 21
2.1.2. Role of Deleterious Germline or Somatic HRR Gene Alterations in Prostate Cancer 21
2.2. Background 22
2.2.1. Summary of Available Nonclinical and Clinical Data for Niraparib 22
2.2.2. Summary of Available Nonclinical and Clinical Data for AA Plus Prednisone 23
2.2.3. Summary of Available Clinical Data for Niraparib and AA Plus Prednisone in Prostate Cancer 24
2.3. Benefit-Risk Assessment 26

3. OBJECTIVES AND ENDPOINTS 27

4. STUDY DESIGN 29
4.1. Overall Design 29
4.2. Scientific Rationale for Study Design 30
4.2.1. Participant Input into Design 31
4.2.2. Study-Specific Ethical Design Considerations 32
4.3. Justification for Dose 32
4.4. End of Study Definition 33

5. STUDY POPULATION 33
5.1. Inclusion Criteria 35
5.2. Exclusion Criteria 37
5.3. Lifestyle Considerations 39
5.4. Screen Failures 40

6. STUDY MEDICATION AND CONCOMITANT THERAPY 41
6.1. Study Medication(s) Administered 41
6.1.1. ADT Administration 41
6.1.2. Study Medication 41
6.1.3. Special Dosing Instructions for Pharmacokinetic Visits 45
6.2. Preparation/Handling/Storage/Accountability 45
6.3. Measures to Minimize Bias: Randomization and Blinding 46
6.4. Study Medication Compliance 47
6.5. Dose Modification 48
6.5.1. Posterior Reversible Encephalopathy Syndrome (PRES) 52
6.6. Continued Access to Study Medication After the End of the Study 52
6.7. Treatment of Overdose 53
6.8. Concomitant Therapy 53
6.8.1. Permitted Supportive Care and Interventions 53
6.8.2. Prohibited Concomitant Therapies 54
6.8.3. Restricted Concomitant Medications 54

7. DISCONTINUATION OF STUDY MEDICATION AND PARTICIPANT DISCONTINUATION/WITHDRAWAL 55

7.1. Discontinuation of Study Medication 55
7.2. Participant Discontinuation/Withdrawal From the Study 55
7.2.1. Withdrawal From the Use of Research Samples 57
7.3. Lost to Follow-up 57

8. STUDY ASSESSMENTS AND PROCEDURES 58
8.1. Efficacy Assessments 61
8.1.1. Evaluations for Primary Efficacy Assessment 61
8.1.1.1. Criteria for Primary Endpoint 61
8.1.2. Evaluations for Other Efficacy Assessments 62
8.1.2.1. Patient Reported Outcomes 62
8.1.2.1.1. BPI-SF 63
8.1.2.1.2. FACT-P 63
8.1.2.1.3. EQ-5D-5L 63
8.1.2.1.4. PRO CTCAE 64
8.1.2.2. ECOG Performance Status 64
8.2. Safety Assessments 64
8.2.1. Physical Examinations 64
8.2.2. Vital Signs 64
8.2.3. ECGs 65
8.2.4. Clinical Safety Laboratory Assessments 65
8.3. Adverse Events and Serious Adverse Events 66
8.3.1. Time Period and Frequency for Collecting Adverse Event and Serious Adverse Event Information 66
8.3.2. Method of Detecting Adverse Events and Serious Adverse Events 67
8.3.3. Follow-up of Adverse Events and Serious Adverse Events 67
8.3.4. Regulatory Reporting Requirements for Serious Adverse Events and Anticipated Events 67
8.3.5. Pregnancy 68
8.3.6. Disease-Related Events and Disease-Related Outcomes Not Qualifying as Adverse Events or Serious Adverse Events 68
8.3.7. Adverse Events of Special Interest 69
8.4. Pharmacokinetics 69
8.4.1. Evaluations 69
8.4.2. Analytical Procedures 69
8.4.3. Pharmacokinetic Parameters and Evaluations 70
8.5. Genetics 70
8.6. Biomarkers 70
8.6.1. Evaluations 71
8.6.1.1. Plasma for RNA 71
8.6.1.2. Plasma for DNA 71
8.6.1.3. Tumor Tissue Analysis 71
8.7. Medical Resource Utilization and Health Economics 71
8.8. Qualitative Exit Interview 72

9. STATISTICAL CONSIDERATIONS 72
9.1. Statistical Hypotheses 72
9.2. Sample Size Determination 73
9.3. Populations for Analyses 73
9.4. Statistical Analyses 73
9.4.1. General Considerations 74
9.4.1.1. Multiplicity Adjustment for Testing of Primary and Key Secondary Endpoints 74
9.4.2. Primary Endpoint 74
9.4.3. Secondary Endpoints 74
9.4.4. Other Endpoints 74
9.4.4.1. Patient-reported Outcomes Analyses 75
9.4.5. Safety Analyses 75
9.4.6. Other Analyses 76

9.5. Interim Analysis for Overall Survival 78
9.6. Data Monitoring Committee or Other Review Board 78

10. SUPPORTING DOCUMENTATION AND OPERATIONAL CONSIDERATIONS 79
10.1. Appendix 1: Abbreviations 79
10.2. Appendix 2: Regulatory, Ethical, and Study Oversight Considerations 81
REGULATORY AND ETHICAL CONSIDERATIONS 81
FINANCIAL DISCLOSURE 84
INFORMED CONSENT PROCESS 84
RECRUITMENT STRATEGY 85
DATA PROTECTION 85
LONG-TERM RETENTION OF SAMPLES FOR FUTURE RESEARCH 86
COMMITTEES STRUCTURE 86
PUBLICATION POLICY/DISSEMINATION OF CLINICAL STUDY DATA 87
DATA QUALITY ASSURANCE 88
CASE REPORT FORM COMPLETION 88
SOURCE DOCUMENTS 89
MONITORING 90
ON-SITE AUDITS 90
RECORD RETENTION 90
STUDY AND SITE START AND CLOSURE 91
10.3. Appendix 3: Additional Information on CYP450 Drug Interactions 92
10.4. Appendix 4: Adverse Events, Serious Adverse Events, Product Quality Complaints, and Other Safety Reporting: Definitions and Procedures for Recording, Evaluating, Follow-up, and Reporting 93
ADVERSE EVENT DEFINITIONS AND CLASSIFICATIONS 93
ATTRIBUTION DEFINITIONS 94
SEVERITY CRITERIA 94
SPECIAL REPORTING SITUATIONS 94
PROCEDURES 95
CONTACTING SPONSOR REGARDING SAFETY 96
PRODUCT QUALITY COMPLAINT HANDLING 96
10.5. Appendix 5: Guidance on Study Conduct During the COVID-19 Pandemic 98
GUIDANCE SPECIFIC TO THIS PROTOCOL 98
10.6. Appendix 6: ECOG Performance Status 101
10.7. Appendix 7: Clinical Laboratory Tests 102
10.8. Appendix 8: Anticipated Adverse Events 103
10.9. Appendix 9: Child-Pugh Criteria 105
10.10. Appendix 10: Open-label Extension Phase 106
10.11. Appendix 11: Long-term Extension Phase 110
10.12. Appendix 12: Protocol Amendment History 112

11. REFERENCES 124

INVESTIGATOR AGREEMENT 127

LIST OF IN-TEXT TABLES AND FIGURES

TABLES

Table 1: Schedule of Activities for Prescreening Phase[a] ... 13
Table 2: Schedule of Activities for Screening, Treatment, and Follow-up Phases[a] ... 14
Table 3: Schedule of Pharmacokinetic Assessments ... 19
Table 4: Eligible HRR Gene Alterations ... 31
Table 5: Description of Study Medications ... 43
Table 6: Single-Agent Formulations ... 44
Table 7: Low-strength niraparib FDC Formulations for Use When Dose Modification is Required and When Available ... 44
Table 8: Guidelines for Dose Modification ... 48
Table 9: Guidelines for Dose Modification on Specific Adverse events ... 49
Table 10: Minimum Clinically Important Differences ... 75

FIGURES

Figure 1: Schematic Overview of the Study ... 12
Figure 2. Determination of Molecular Eligibility ... 34

1. PROTOCOL SUMMARY

1.1. Synopsis

A Phase 3 Randomized, Placebo-controlled, Double-blind Study of Niraparib in Combination with Abiraterone Acetate and Prednisone Versus Abiraterone Acetate and Prednisone for the Treatment of Participants with Deleterious Germline or Somatic Homologous Recombination Repair (HRR) Gene-Mutated Metastatic Castration-Sensitive Prostate Cancer (mCSPC)

Synopsis based on Protocol 67652000PCR3002 Amendment 4, finalized on 23August 2023.

IND: 131190

EudraCT number: 2020-002209-25

EU Trial Number: 2023-506365-64

Niraparib is an orally available, highly selective poly (adenosine diphosphate-ribose) polymerase (PARP) inhibitor, with potent activity against PARP-1 and PARP-2 deoxyribonucleic acid-repair polymerases.

Abiraterone acetate (AA), the prodrug of abiraterone, inhibits cytochrome P450c17, a critical enzyme in androgen biosynthesis.

Note: The term mCSPC used throughout this document is synonymous with metastatic hormone-sensitive prostate cancer (mHSPC).

BENEFIT-RISK ASSESSMENT

The combination of niraparib and AA, plus prednisone is hypothesized to have a positive benefit-risk profile when used for the treatment of patients with mCSPC, as proposed for this study.

Taking into account the measures incorporated to minimize risk to participants of this study, the potential risks identified in association with niraparib/AA fixed-dose combination (FDC), niraparib and AA, plus prednisone are justified by the anticipated benefits that may be afforded to participants with mCSPC.

OBJECTIVES AND ENDPOINTS

- To determine if niraparib and AA, plus prednisone compared with AA plus prednisone in participants with deleterious germline or somatic HRR gene-mutated mCSPC provides superior efficacy in improving radiographic progression-free survival (rPFS).
- To assess the clinical benefit of niraparib and AA, plus prednisone compared with AA plus prednisone in participants with deleterious germline or somatic HRR gene-mutated mCSPC.
- To characterize the safety profile of niraparib and AA, plus prednisone compared with AA plus prednisone in participants with deleterious germline or somatic HRR gene-mutated mCSPC.

HYPOTHESIS

The primary hypothesis of this study is that niraparib and AA, plus prednisone will improve rPFS compared with AA plus prednisone in participants with deleterious germline or somatic HRR gene-mutated mCSPC.

OVERALL DESIGN

This is a randomized, placebo-controlled, double-blind, multinational Phase 3 treatment study to evaluate the safety and efficacy of niraparib and AA, plus prednisone compared with AA plus prednisone in men over the age of 18 years with deleterious germline or somatic HRR gene-mutated mCSPC.

Approximately 692 participants will be randomly assigned in a 1:1 ratio to either niraparib 200 mg, and AA 1000 mg, plus prednisone 5 mg daily or AA 1000 mg plus prednisone 5 mg daily. All participants must be receiving background androgen deprivation therapy (ADT; ie, gonadotropin-releasing hormone analogue (GnRHa) or surgical castration).

The study will consist of 4 phases: a Prescreening Phase for biomarker evaluation for eligibility only, a Screening Phase, a Treatment Phase, and a Follow-up Phase.

Efficacy, safety, pharmacokinetics (PK), and biomarkers will be assessed according to the Schedule of Activities (SoA).

Treatment is continuous; however, a treatment cycle is defined as 28 days. Study medication should continue until disease progression, unacceptable toxicity, death, withdrawal of consent, or termination of the study by the sponsor. Patients with radiographic progression can remain on therapy if still receiving clinical benefit.

Participants will be followed until death or termination of the study. In addition to survival follow-up, data will continue to be collected to evaluate all of the secondary and other endpoints. The Euro-Quality of Life Questionnaire will also be administered for up to 1 year after study medication discontinuation.

Participants will be monitored for safety during the Prescreening, Screening, and Treatment Phases and up to 30 days after the last dose of study medication during the follow-up phase. Adverse events (AEs) including clinically significant laboratory abnormalities reported as AEs, will be graded and summarized using National Cancer Institute Common Terminology Criteria for Adverse Events, Version 5.

An Independent Data Monitoring Committee will be commissioned for this study and will perform regular review of study data.

NUMBER OF PARTICIPANTS

Approximately 692 participants will be randomly assigned in this study.

TREATMENT GROUPS AND DURATION

Participants will be randomized in a 1:1 ratio to 1 of 2 treatments groups:

- niraparib 200 mg and AA 1000 mg, plus prednisone 5 mg daily (Experimental)
- AA 1000 mg plus prednisone 5 mg daily (Control).

All study medications will be administered orally once daily. Matching placebos will also be administered.

EFFICACY EVALUATIONS

The primary endpoint for this study is rPFS as assessed by the investigator which will be evaluated using computed tomography or magnetic resonance imaging scans and whole-body bone scans (Technetium-99m). Evaluation of rPFS will also be assessed by blinded independent central review. Additional efficacy assessments are described in the SoA.

PHARMACOKINETIC EVALUATIONS

Venous blood samples collected to measure plasma concentrations are described in the SoA.

BIOMARKER EVALUATIONS

Deleterious germline or somatic HRR gene alterations will be evaluated using the sponsor's approved assays to determine molecular eligibility at prescreening from (a) tumor tissue (archival or recently collected), and (b) germline (eg, blood, saliva), and also (c) plasma (if test is available from the sponsor). The type of samples, testing methodology, and gene alterations to determine eligibility may be updated by the sponsor and any changes will be communicated to investigators by letter. In lieu of prescreening, prior local HRR testing performed in a Clinical Laboratory Improvement Amendment-certified (CLIA) or equivalent laboratory may be used. Sponsor must approve participant's HRR alteration status prior to randomization based on receipt of a redacted biomarker report(s).

All participants will have additional exploratory research samples collected where local regulations allow.

PHARMACOGENOMIC (DNA) EVALUATIONS

A blood or saliva sample will be collected to allow for pharmacogenomic evaluation of deleterious germline HRR gene alterations (where local regulations permit). Additional research may be done on collected samples to identify predictors of response to niraparib and AA or to allow for bridging and/or diagnostic test development where local regulations allow.

SAFETY EVALUATIONS

Safety assessments will be based on review of AE reports and the results of heart rate and blood pressure measurements, electrocardiograms (ECGs), physical examinations, and clinical safety laboratory tests at specified timepoints described in the SoA.

STATISTICAL METHODS

Efficacy

Approximately 692 participants will be randomized in a 1:1 ratio to receive niraparib and AA, plus prednisone or AA plus prednisone. The primary endpoint of rPFS and other event-driven secondary endpoints will be analyzed when sufficient events have been observed.

Safety

Safety parameters to be analyzed are the incidence, intensity, and type of AEs and clinically significant changes in the participant's physical examination, ECGs, heart rate, blood pressure, and clinical laboratory results.

Pharmacokinetics

If feasible, population PK analysis of plasma concentration-time data and exposure-response analysis for selected efficacy and safety endpoints may be performed.

1.2. Schema

Figure 1: Schematic Overview of the Study

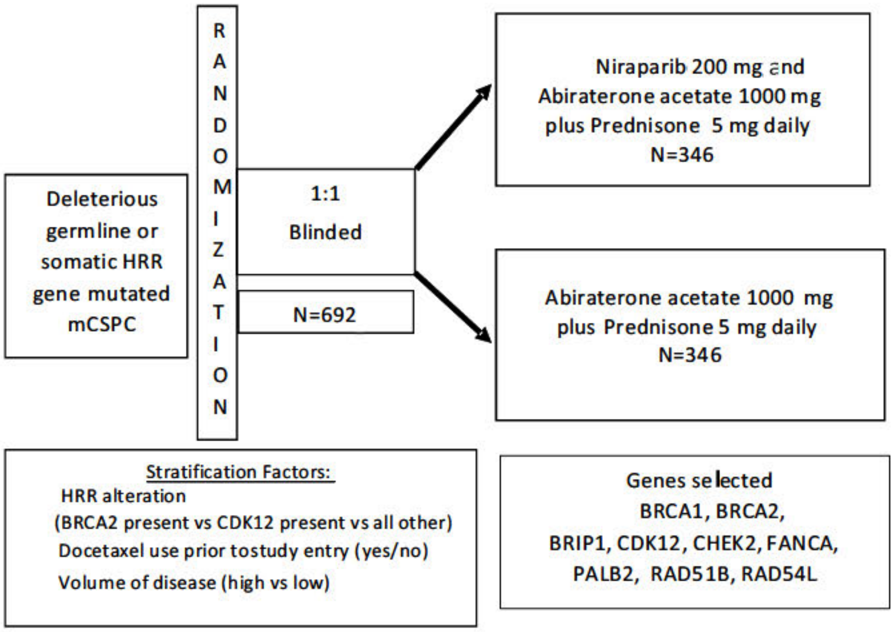

BRCA=breast cancer gene; BRIP1=BRCA1 interacting protein C terminal helicase 1; CDK12=cyclin-dependent kinase 12; CHEK2=checkpoint kinase 2; FANCA=Fanconi anemia complementation group A; HRR=homologous recombination repair; mCSPC=metastatic castration-sensitive prostate cancer; PALB2=partner and localizer of BRCA2; RAD51B=RAD51 paralog B, RAD54L=RAD54-like

1.3. Schedule of Activities (SoA)

Table 1: Schedule of Activities for Prescreening Phase[a]

Study Procedure	Notes
Prescreening Informed Consent	Must be obtained (written or remote/virtual) before first study-related procedure
Biomarker samples for eligibility – submit (if test is available from the sponsor):<br>1. Tumor tissue<br>2. Germline sample (eg. Blood, saliva)<br>3. Plasma	The type of samples and testing methodology to determine eligibility may be updated by the sponsor and any changes will be communicated to investigators by letter.
Prescreening related serious adverse events	Only serious adverse events related to study procedures should be reported from the time the prescreening informed consent is obtained.

a. For participants who do not have prior results available from local testing, performed at a Clinical Laboratory Improvement Amendment-certified (CLIA) or equivalent laboratory.

Table 2: Schedule of Activities for Screening, Treatment, and Follow-up Phases[a]

Phase	Screening	Treatment									Follow-up	Notes
**Cycle**<br>(Each treatment cycle is 28 days)	≤35 days before randomization unless otherwise specified	1		2		3		4 to 24	25 to EoT<br><br>Every 4 cycles	EoT	Every 4 months	**EoT** visit: within 30 days of last dose of study medication or prior to start of a new anti-prostate cancer therapy, whichever occurs first.<br>**Follow-up**: until death, lost to follow-up, withdrawal of consent, or study termination.
**Day**		1	15	1	15	1	15	1	1			
**Visit Window**		±3 d							±14 d		±4 wks	
**Study Procedure**												
**Screening/ Administration**												
Informed consent	X[b]											Written consent must be obtained before first study-related procedure
Demographics	X											
Inclusion/ Exclusion criteria	X											Minimum criteria for the availability of documentation supporting eligibility criteria are described in the Source Documents section of Appendix 2, Regulatory, Ethical, and Study Oversight Considerations.<br>Check clinical status again before randomization and first dose of study medication.
Randomization												All participants should commence study medication within 72 hours or 3 calendar days after randomization
Dispense study medication		*Every site visit on Day 1*										
Administer study medication		*Continuous daily dosing*										
Dosing compliance and study medication accountability		*Every site visit on Day 1*								X		

Table 2: Schedule of Activities for Screening, Treatment, and Follow-up Phases[a]

Phase	Screening	Treatment									Follow-up	Notes
**Cycle** (Each treatment cycle is 28 days)	≤35 days before randomization unless otherwise specified	1		2		3		4 to 24	25 to EoT<br>Every 4 cycles	EoT	Every 4 months	**EoT** visit: within 30 days of last dose of study medication or prior to start of a new anti-prostate cancer therapy, whichever occurs first.<br>**Follow-up**: until death, lost to follow-up, withdrawal of consent, or study termination.
**Day**		1	15	1	15	1	15	1	1			
**Visit Window**		±3 d							±14 d		±4 wks	
**Study Procedure**												
**Efficacy Assessments**												
CT or MRI (chest, abdomen, and pelvis) AND ^{99m}Tc Bone Scan	X					*Day 1 of C3, C5, then every 4 cycles and EoT (not needed at EoT if previously done ≤6 weeks)*					X*	^{99m}Tc bone scans or CT/MRI may occur within ± 7 days of disease evaluation visit. Unscheduled assessments can occur at any time as needed if signs of disease progression are observed.<br>*Until radiographic progression. See Section 8.1.1 for details.
Medical Resource utilization		*Collected continuously from randomization*										
Survival Status, Subsequent Therapy, and Disease Progression Status on the Subsequent Therapy		*Collected continuously from randomization, including during the Follow up Phase*										May be obtained by telephone or chart review.[c]
Electronic Patient Reported Outcomes Includes: BPI-SF, FACT-P, EQ-5D-5L (only EQ-5D-5L in follow-up), and PRO-CTCAE	X	X		X		X		X	X	X	X	Complete before any tests, procedures, or other consultations for that visit.<br>PRO-CTCAE for US sites only.<br>The questionnaires may be completed remotely, and the last assessment will be at 12 months after discontinuation of study medication.
ECOG PS	X	X		X		X		X	X	X		
**Safety Assessments**												
Physical Examination	X	X		X		X		X	X	X		

Table 2: Schedule of Activities for Screening, Treatment, and Follow-up Phases[a]

Phase	Screening	Treatment									Follow-up	Notes
**Cycle**<br>(Each treatment cycle is 28 days)	≤35 days before randomization unless otherwise specified	1		2		3		4 to 24	25 to EoT<br><br>Every 4 cycles	EoT	Every 4 months	**EoT** visit: within 30 days of last dose of study medication or prior to start of a new anti-prostate cancer therapy, whichever occurs first.<br>**Follow-up**: until death, lost to follow-up, withdrawal of consent, or study termination.
**Day**		1	15	1	15	1	15	1	1			
**Visit Window**		±3 d							±14 d		±4 wks	
**Study Procedure**												
**Vital Signs**												
Blood pressure and Heart rate	X	Cycle 1 & Cycle 2: Days 1, 8, 15, 22				X	X	X	X	X		Blood pressure and heart rate monitoring can occur at clinic visit or may also be reported to the site by a home nurse or other qualified medical professionals approved by the investigator and authorized to examine patients.
12-lead ECG	X	*As clinically indicated*										
**Clinical Laboratory Tests**												
Hematology and Potassium	X	Cycle 1: Days 1, 8, 15, 22		X	X	X	X	X	X	X		During screening, labs must be obtained within 14 days of randomization. See Appendix 7.
Chemistry	X	X		X		X		X	X	X		During screening, labs must be obtained within 14 days of randomization. See Appendix 7.
PSA	X	X		X		X		X	X	X		Prior to randomization, obtain at least 2 PSA samples separated by at least 1 week while patient is on ADT to confirm that the patient does not meet PCWG3 criteria for PSA progression to mCRPC. These PSA samples can be obtained as SOC or as part of the Covance screening tests.
LFTs	X	X	X	X	X	X	X	X	X	X		See Appendix 7. Clinical Laboratory Tests for a list of LFT assessments.

Table 2: Schedule of Activities for Screening, Treatment, and Follow-up Phases[a]

Phase	Screening	Treatment									Follow-up	Notes
**Cycle** (Each treatment cycle is 28 days)	≤35 days before randomization unless otherwise specified	1		2		3		4 to 24	25 to EoT<br><br>Every 4 cycles	EoT	Every 4 months	**EoT** visit: within 30 days of last dose of study medication or prior to start of a new anti-prostate cancer therapy, whichever occurs first.<br>**Follow-up**: until death, lost to follow-up, withdrawal of consent, or study termination.
**Day**		1	15	1	15	1	15	1	1			
**Visit Window**		±3 d							±14 d		±4 wks	
**Study Procedure**												
Coagulation (PT/INR and aPTT)	X											Additional samples can be collected if clinically indicated during the trial.
**Clinical Pharmacology Assessments**												
Pharmacokinetic sampling	See Schedule of Activities – Pharmacokinetic Assessments (Table 3)											
**Pharmacodynamics, Biomarkers (eg, plasma, serum, urine, biopsy sample collection; where local regulations allow), and Diagnostics**												
Plasma for RNA[d]		X				X				X		EoT samples should be collected when feasible.
Plasma for DNA[d]		X				X				X		EoT samples should be collected when feasible
Biomarker analysis of tumor tissue for participants who provided a local result.					X							Willing to provide archival or fresh tumor tissue. Participants that submitted laboratory result from the sponsor's commercial tumor tissue test do not need to provide tissue.
**Pharmacogenomics (DNA)**												
Blood sample collection[d]		X										Sample should be collected at the specified time point. However, if necessary, it may be collected at a later time point.
**Ongoing Participant Review**												
Concomitant therapy						X						Collect continuously from informed consent until 30 days after the last dose of study medication
Adverse events						X						
Qualitative Exit Interview										X		In consenting participants at selected study sites

Abbreviations: aPTT=activated partial thromboplastin time; BPI-SF=Brief Pain Inventory-Short Form; C=cycle; CT=computed tomography; d=day; DNA=deoxyribonucleic acid; ECG=electrocardiogram; ECOG PS=Eastern Cooperative Oncology Group performance status; eCRF=electronic case report form;

EoT = End-of-Treatment; EQ-5D-5L=Euro Quality of Life Questionnaire; FACT-P=Functional Assessment of Cancer Therapy – Prostate Questionnaire; INR= International normalized ratio; PRO-CTCAE= Patient-reported Outcomes Common Terminology Criteria for Adverse Events; PT=prothrombin time; LFT=liver function tests; m=month; MRI=magnetic resonance imaging; PSA=prostate-specific antigen; q=every; RNA=ribonucleic acid; ^{99m}Tc=technetium-99m

a. The Open-label Extension Phase and/or Long-term Extension Phase will begin upon notification from the Sponsor. At that time, the applicable procedures should be followed, as detailed in Appendix 10: Open-label Extension Phase and Appendix 11: Long-term Extension Phase.
b. Re-consenting may be done remotely/virtually as per local regulations and in accordance with the sponsor policy.
c. May be obtained at a shorter interval than every 4 months during follow-up if required for database lock.
d. Where local regulations permit.

Table 3: Schedule of Pharmacokinetic Assessments

Phase	Treatment								
Cycle	Cycle 2[a]			Cycle 3[a]			Cycles 4 to 7[b]		
Day	1			1			1		
Visit Window	±3 Days								
Time	Predose	0	+1-3 h	Predose	0	+1-3 h	Predose	0	≥+3 h
Administration of study medication		X			X			X	
PK blood sample	X		X	X		X	X	**or**	X

PK=pharmacokinetic

a. On Day 1 of Cycles 2 and 3, study medications should be taken at the site under the supervision of the investigator or designee and should not be taken at home on the morning of the visits. If the participant takes the study medications prior to the visit, the PK samples should be obtained at the next cycle.
b. On Day 1 of Cycles 4 through 7, 1 PK sample is to be obtained either predose or at least 3 hours postdose. If the study visit is in the morning, participants should refrain from taking the study medications until after the PK sample is obtained at the investigative site. If the visit is in the afternoon, the participant should take their study medications in the morning as usual and the PK sample will be collected during the study visit.

Note: The date and time of the PK samples and of the study medications administered at the investigative site and prior doses taken at home should be recorded.

2. INTRODUCTION

Niraparib is an orally available, highly selective poly (adenosine diphosphate [ADP]-ribose) polymerase (PARP) inhibitor (PARPi), with potent activity against PARP-1 and PARP-2 deoxyribonucleic acid (DNA)-repair polymerases.[29] Niraparib is currently being studied in breast cancer, ovarian cancer, and non-small cell lung cancer. Niraparib was approved in the United States (US) by the Food and Drug Administration on 27 March 2017 for the maintenance treatment of adult patients with recurrent epithelial ovarian, fallopian tube, or primary peritoneal cancer who are in a complete or partial response to platinum-based chemotherapy. Niraparib also received European Commission approval (16 November 2017) for use as a monotherapy for the maintenance treatment of adult patients with platinum-sensitive relapsed high grade serous epithelial ovarian, fallopian tube, or primary peritoneal cancer who are in response (complete or partial) to platinum-based chemotherapy (EMA/CHMP/574018/2017).

Abiraterone acetate [AA; JNJ-212082]) is a pro-drug of abiraterone (JNJ-589485) 17-(3-pyridyl) androsta-5,16-dien-3β-ol], which is an androgen biosynthesis inhibitor. Abiraterone selectively inhibits the enzyme cytochrome P450 (CYP)c17, which is found in the testes and adrenals, as well as in prostate tissues and tumors.[40] The clinical benefits of AA plus prednisone or prednisolone (AAP) for the treatment of prostate cancer were demonstrated in 3 large, randomized, double-blind, placebo-controlled Phase 3 studies. These studies included patients with metastatic castration-resistant prostate cancer (mCRPC) post-chemotherapy (COU-AA-301), chemotherapy-naïve patients (COU-AA-302), and patients with high-risk metastatic hormone-naïve disease (LATITUDE).[11,20,42,45] Abiraterone acetate plus prednisone is currently approved in more than 100 countries worldwide for the treatment of men with metastatic prostate cancer (exact wording of indications varies by region).[34]

This study evaluates the efficacy of niraparib and AA, plus prednisone compared with AA plus prednisone in men with deleterious germline or somatic homologous recombination repair (HRR) gene-mutated metastatic castration-sensitive prostate cancer (mCSPC) receiving androgen deprivation therapy (ADT).

For the most comprehensive nonclinical and clinical information regarding niraparib and AA refer to the latest version of the Investigator's Brochure (IB) and Addenda for niraparib[25] and AA.[26] Information regarding the niraparib/AA fixed-dose combination (FDC) is in the niraparib/AA FDC IB.[24]

The term “study medication” throughout the protocol, refers to study drugs (ie, FDC, niraparib, AA, or relevant placebo, and prednisone).

The term "sponsor" used throughout this document refers to the entities listed in the Contact Information page(s), which will be provided as a separate document.

The term "participant" throughout the protocol refers to the common term "subject".

The term mCSPC used throughout this document is synonymous with metastatic hormone-sensitive prostate cancer (mHSPC).

2.1. Study Rationale

2.1.1. Background on mCSPC

Prostate cancer is the second most common cancer in men worldwide, with an estimated annual global incidence in 2018 of 1.3 million and mortality of 358,989.[4] In the United States, in 2019, the incidence was estimated at 174,650 new cases with 31,620 deaths.[51] In 2015 in the European Union (EU), the incidence of new cases was estimated at 365,000 with 77,000 deaths.[9] For patients who develop metastatic disease, the 5-year relative survival is only 30%.[54] Prognostic factors that influence survival in mCSPC include high prostate-specific antigen (PSA) at diagnosis, high Gleason score, higher primary tumor stage, worse World Health Organization (WHO) performance status, younger age, and the presence of bone metastases. Bone metastases are the most common site of metastases in patients with prostate cancer and are a major cause of morbidity and mortality, and therefore pose a substantial burden as they are associated with skeletal-related events, pain, and the need for radiation therapy or surgery to bone.[52]

2.1.2. Role of Deleterious Germline or Somatic HRR Gene Alterations in Prostate Cancer

Poly (adenosine diphosphate-ribose) polymerases are a family of nuclear protein enzymes involved with the DNA damage response.[15] PARP-1 and PARP-2 are zinc-finger DNA-binding polymerases that detect damaged DNA and promote DNA repair by several mechanisms. After detecting DNA damage, PARP activates the base excision repair pathway via an intracellular signaling mechanism. In cells incapable of DNA repair via homologous repair due to deleterious alterations, PARP inhibition leads to irreparable DNA double-strand breaks, collapsed replication forks, and an increased use of the non-homologous end joining pathway. These disruptions result in genomic instability and ultimately cell death. Treatment with a PARPi represents an opportunity to selectively kill cancer cells with deficiencies in homologous recombination and other DNA repair mechanisms.[18]

Prostate cancer is a heterogenous disease, and not all patients have the same response to therapy. Recent genomic analyses have highlighted specific germline and somatic alterations and alternative driver growth signaling pathways in patients with metastatic disease.[2,44,57] Deleterious germline or somatic homologous HRR gene alterations, with breast cancer gene (BRCA) being the most common, in particular are associated with aggressive disease, rapid progression rates, treatment resistance, and poor prognosis with death at an early age.[1,2,36] Up to 20% to 30% of advanced prostate cancers may harbor these deleterious germline or somatic HRR gene alterations.[14,32]

Based on recent clinical information, National Comprehensive Cancer Network (NCCN) guidelines (v4 Feb 2019)[37] recommend testing for alterations in HRR genes in tumors from patients with mCRPC and also screening for germline alterations in breast cancer 2 (BRCA2) and other DNA repair damage genes in all patients with high-risk localized prostate cancer and

metastatic disease. However, currently there is no approved targeted therapy tailored for patients with mCSPC and deleterious germline or somatic HRR gene-mutated mCSPC. Current treatment management sequences result in shorter responses and shorter survival in patients carrying deleterious germline or somatic HRR gene alterations such as BRCA2.[39] The Spanish PROREPAIR-B Study demonstrated that patients with mCSPC who were carrying breast cancer 1 (BRCA1), (BRCA2), ataxia telangiectasia mutated (ATM), or partner and localizer of BRCA2 (PALB2) germline alterations had shorter median time to reach castration resistance from the start of ADT: 18.6 months (range: 10.6 to 26.6 months) in carriers versus 28.4 months (range: 24.2 to 32.6 months) in non-carriers. Progression to mCRPC in the ATM/BRCA1/BRCA2/PALB2 group was frequently accompanied by radiographic progression (61.5%; p 0.015 versus noncarriers), particularly in BRCA2 carriers (71.4%; p 0.011 versus noncarriers). When DNA-repair gene defect (DRD) positive patients developed mCRPC, the response to first next generation androgen-receptor (AR)-targeting agent was poor, and cause specific survival was halved in germline BRCA2 carriers (17.4 versus 33.2 months; p 0.027), and germline BRCA2 alterations were identified as an independent prognostic factor for cause-specific survival (hazard ratio [HR] 2.11; p 0.033).[5] Thus, deleterious germline or somatic HRR gene alterations, particularly BRCA2, are prognostic for a high risk of tumor progression and predictive of the development of resistance to androgen targeted therapies. Cyclin dependent kinase 12 (CDK12) is thought to have a distinct role in maintaining genomic stability. In prostate cancer, inactivating CDK12 alterations are found in 6-7% of cases.[3]

In mCRPC patients with DNA-repair anomalies, previously treated with 1 line of taxane-based chemotherapy and ≥1 line of AR-targeted therapy, niraparib monotherapy at a dose of 300 mg has demonstrated activity (sponsor study 64091742PCR2001). The effectiveness of PARPi in patients with deleterious germline or somatic HRR gene-mutated prostate cancer was further justified in the PROfound study, a Phase 3 study in second line mCRPC patients comparing olaparib to physician's choice of either enzalutamide or AAP.[11] The primary endpoint was radiographic progression-free survival (rPFS) by blinded independent central review in patients with alterations in BRCA1, BRCA2, and ATM. Patients that were in the olaparib arm had a median rPFS of 7.4 months versus patients in the physician's choice arm who had a median rPFS of 3.6 months (HR 0.34 [0.25, 0.47] $p<0.0001$). In a sub-analysis, patients harboring BRCA2 had a median rPFS of 10.84 months when treated with olaparib, while patients treated with either enzalutamide or AAP had a median rPFS of 3.48 months. The PROfound sub-analysis also showed patients harboring CDK12 gene alterations treated with olaparib had a median rPFS of 5.09 months while those treated with either AAP or enzalutamide had an rPFS of 2.20 months. Based on this data, patients with CDK12 gene alterations are being included in this protocol.

2.2. Background

2.2.1. Summary of Available Nonclinical and Clinical Data for Niraparib

A brief summary of the nonclinical and clinical information available for niraparib to date is provided below. For the most comprehensive nonclinical and clinical information regarding niraparib, refer to the latest version of the niraparib IB.[25]

In an in vitro model, niraparib inhibited PARP-1 and PARP-2 activity with a half-maximal inhibitory concentration (IC_{50}) of 3.8 and 2.1 nM, respectively. In cultured cells, niraparib inhibited PARP-dependent PARylation stimulated by DNA damage with an IC_{50} of 4 nM and a 90% IC_{90} of 40 nM. When a PARPi such as niraparib is used to treat tumor cells or xenograft tumors with HRR pathway bearing alterations for BRCA1 and BRCA2, the tumor cell is unable to efficiently and accurately repair DNA double-strand breaks, which creates a synthetic lethal condition leading to tumor cell death and ultimately tumor growth inhibition/improved survival.[30]

Niraparib is currently approved for the treatment of epithelial ovarian, fallopian tube, or primary peritoneal cancer in multiple lines of therapy[58] and is currently being studied in prostate cancer, breast cancer, ovarian cancer, and non-small cell lung cancer.

In the Phase 3 registration NOVA study (NCT01847274) for patients with ovarian cancer previously treated with platinum-based chemotherapy, the most common grade 3/4 adverse events (AE) were thrombocytopenia (29%), anemia (25%), and neutropenia (20%),[58] which represent the identified important risk of hematological toxicity. The other identified risk is hypertension. Important potential risks include myelodysplastic syndrome/acute myeloid leukemia (MDS/AML), second primary malignancy, embryo fetal toxicity, pneumonitis and thrombo-embolic events.

Niraparib is being evaluated as monotherapy in sponsor Study 64091742PCR2001 for the treatment of patients with mCRPC and DNA-repair anomalies previously treated with at least 1 taxane-based therapy and at least 1 line of AR-directed therapy. As of 23 May 2019, an objective response rate of 41% was observed in 46 BRCA patients with measurable disease at baseline.[53] Preliminary data from this study in 165 patients show that niraparib 300 mg as monotherapy for the treatment of patients with mCRPC and DRD has a safety profile that is similar to the known safety profile of the drug for the treatment of platinum-sensitive ovarian cancer. Hematologic toxicities are a known side effect of niraparib and were also observed in this study (grade 3/4 hematologic toxicities: anemia 29%, thrombocytopenia 15%, and neutropenia 7%); however, the hematologic toxicities are manageable with standard outpatient care and dose interruption/reduction. The most common grade 3 or 4 nonhematologic adverse events were fatigue and back pain (6% each).

2.2.2. Summary of Available Nonclinical and Clinical Data for AA Plus Prednisone

A brief summary of the clinical information available for AAP is provided below. For the most comprehensive nonclinical and clinical information regarding AAP, refer to the latest version of the AA IB.[26]

Abiraterone acetate, the prodrug of abiraterone, inhibits CYP17A1, a critical enzyme in androgen biosynthesis. Abiraterone acetate in combination with prednisone is approved for the treatment of patients with mCRPC and metastatic high-risk castration-sensitive prostate cancer (mCSPC).

The approval in mCSPC is based on a large, randomized, double blind, placebo-controlled Phase 3 study (LATITUDE).[21,21] In the LATITUDE study, 1,199 men with newly diagnosed mCSPC were

randomly assigned to ADT plus AA 1000 mg plus prednisone 5 mg once daily or to ADT plus placebo. Patients had high-risk disease (cM1) with the presence of at least 2 of 3 high-risk parameters: Gleason score ≥8, at least 3 bone lesions, and the presence of measurable visceral metastasis. The pre-specified interim analysis after 406 deaths (169 deaths with AAP and 237 with placebo and median follow-up of 30.4 months) showed a statistically significant lower risk of death in the AAP group compared with placebo group (HR 0.62, 95% CI: 0.51, 0.76, p<0.001).[20] Median rPFS was also statistically significantly superior with 33 months in the AAP group and 14.8 months in the placebo group (HR 0.47, 95% CI: 0.39, 0.55, p<0.001). Subsequent therapies that may prolong overall survival (OS) in mCSPC were used by 21% of patients on AAP and 41% of patients on placebo. The overall incidence of AEs was 93% in both groups. Grade 3 or 4 AEs were reported in 63% of patients in the AAP group and 48% in the placebo group. Grade 3 and Grade 4 hypertension were reported in 20% and 0%, respectively, in the AAP group and 10% and 0.2%, respectively, in the placebo group. Grade 3 and Grade 4 hypokalemia were reported in 10% and 0.8%, respectively, in the AAP group and 10% and 0.2%, respectively in the placebo group.

In an updated survival analysis after 618 deaths (275 deaths with AAP and 343 with placebo and median follow-up of 51.8 months), OS was significantly longer in the AAP group (median 53.3 months) compared with the placebo group (median 36.5 months; HR 0.66; 95% CI: 0.56-0.78, p<0.0001).[21] In clinical studies of patients with metastatic prostate cancer, the most common AEs related to AA include peripheral edema, hypokalemia, urinary tract infection, alanine aminotransferase (ALT) increased, aspartate aminotransferase (AST) increased, dyspepsia, hematuria, hypertension, and fractures.[20,59] AA should be used with caution in patients with a history of cardiovascular disease. Caution should be exercised when treating patients whose underlying medical conditions might be compromised by increases in blood pressure, hypokalemia, or fluid retention.

Prednisone may be associated with fatigue, increased appetite, insomnia, weakness, hyperglycemia, ecchymosis, and symptoms related to gastroesophageal reflux. With long-term glucocorticoid therapy, patients may develop Cushing’s syndrome, characterized by central obesity, thin skin, easy bruising, bone loss, avascular necrosis of the hip, cataract and proximal myopathy. Withdrawal of the corticosteroid may result in symptoms that include fever, myalgia, fatigue, arthralgia, and malaise.

2.2.3. Summary of Available Clinical Data for Niraparib and AA Plus Prednisone in Prostate Cancer

A summary of the nonclinical and clinical information available for niraparib and AA plus prednisone to date is provided below. For the most comprehensive nonclinical and clinical information regarding niraparib and AA, refer to the latest versions of the IBs.[26,25,26]

PARP1 has been shown to be a potent modulator of both AR function and response to DNA damage.[50] There are preclinical data to suggest that the combination of niraparib and AAP may be beneficial. In preclinical studies by the sponsor in the human vertebral cancer of the prostate (VCaP) tumor model, mice bearing VCaP tumors were treated with niraparib either alone, or in combination with AA. The combination of niraparib plus AA showed better inhibition of tumor

growth and survival prolongation than the single agents (sponsor data). Therefore, DRD is another mechanism, in addition to AR, which is active in mCSPC, thus simultaneously addressing both targets could portend greater long term benefit in a deleterious germline or somatic HRR gene-mutated population which clearly has a continued unmet medical need.

The sponsor evaluated AA 1000 mg plus prednisone 10 mg in combination with either niraparib 200 mg or 300 mg in a Phase 1b study.[46] Pharmacokinetic (PK) data suggest no drug-drug interaction between niraparib and AAP. The steady-state maximum observed plasma concentration values at 200 mg or 300 mg niraparib in combination with AAP were both within the recommended Phase 2 dose target exposure range of 575-2501 ng/mL. The steady-state area under the concentration time curve $(AUC)_{0\ 24}$ values at 200 mg and 300 mg were also with the recommended Phase 2 dose exposure range of 9159-37324 ng·h/mL. Among 4 patients evaluated in the niraparib 200 mg cohort there were no AE's that met the dose-limiting toxicity (DLT) criteria. Among 8 patients evaluated for DLT in the 300 mg dose, one patient experienced 2 DLT (Grade 3 fatigue and Grade 4 increase in gamma glutamyltransferase). Two additional patients among these 8 had Grade 4 neutropenia on cycle 2, day 1. An additional 15 patients were enrolled in an expansion phase for 200 mg niraparib and AAP, for a total of 19 patients treated with this dose. The most commonly reported treatment-emergent AEs in this cohort were in the system organ classes of Gastrointestinal disorders (nausea [63.2%], vomiting [52.6%], diarrhea [21.1%]) and General disorders and administration site conditions (fatigue [36.8%]) (sponsor's internal data).

Niraparib 200 mg was selected to be the optimal dose to be combined with AA plus prednisone based on no DLT, an overall tolerable safety profile, and no drug-drug interactions detected between AA plus prednisone, and niraparib. A FDC formulation of niraparib and AA is currently being evaluated in several studies including a Phase 1b-2 study of niraparib combination therapies for the treatment of mCRPC (Protocol 64091742PCR2002), as well as a Phase 1 bioavailability and bioequivalence study (Protocol 67652000PCR1001).

The sponsor is also conducting a double-blind, randomized, placebo-controlled Phase 3 study to evaluate the effectiveness of niraparib in combination with AA plus prednisone compared with AA plus prednisone and placebo as first-line therapy in mCRPC patients (Study 64091742PCR3001 [MAGNITUDE]). Cohort 1 of MAGNITUDE enrolled 423 patients with deleterious HRR alterations (HRR+), with the primary endpoint of rPFS by blinded independent central review in the BRCA1/2 subgroup as well as All HRR+ patients. In the primary analysis, niraparib in combination with AAP significantly improved rPFS in both the BRCA1/2 subgroup and the All HRR+ patients, reducing the risk of radiographic progression or death by 47% and 27%, respectively, compared with placebo +AAP. Niraparib in combination with AAP also delayed time to cytotoxic chemotherapy, time to symptomatic progression and improved the objective response rate in All HRR+ patients. No new safety signals were seen in comparison to the niraparib and AAP single agent clinical experiences and the 64091742PCR1001 study. Both Cohorts 1 and 3 (Cohort 3 enrolled HRR+ patients to open-label treatment with the niraparib/AA FDC) remain ongoing.[28]

2.3. Benefit-Risk Assessment

The combination of niraparib and AA, plus prednisone is hypothesized to have a positive benefit-risk profile when used for the treatment of patients with mCSPC, as proposed for this study.

This hypothesis is based on the following:

- Abiraterone acetate plus prednisone is an established standard of care for the treatment of patients with mCSPC and is included in widely accepted clinical treatment guidelines.[35,37]
- The addition of niraparib to the AAP backbone regimen may improve initial disease control and long-term outcomes compared with AAP alone in a biomarker selected population, as discussed in Section 2.2.3.
- While niraparib is an investigational agent in the castration-sensitive prostate cancer population, it has been approved for the treatment of ovarian cancer. The safety profile of niraparib has been characterized in clinical studies as discussed in Section 2.2.1. Data from sponsor study 64091742PCR1001 and 64091742PCR3001 suggest that the safety profile of niraparib 200 mg in combination with AA 1000 mg plus prednisone 10 mg in patients with mCRPC is similar to that described in the ZEJULA® label.[58] Known toxicities for niraparib include gastrointestinal events, hematological events (ie, thrombocytopenia, neutropenia, and anemia), hypokalemia, and hypertension. These toxicities are managed by monitoring the appropriate laboratory values and making the appropriate medical interventions, such as dose interruptions. This protocol includes a targeted monitoring plan and treatment guidelines to ensure appropriate management of toxicities, including hematologic toxicities (see Section 6.5). In addition, participants are required to have specified hematology laboratory values in the inclusion criteria (see Section 5.1) and uncontrolled hypertension is included as an exclusion criterion (see Section 5.2). As noted in the Schedule of Activities (SoA), blood pressure, heart rate, and hematology laboratory values will be monitored through End of Treatment (EoT).
- The toxicities for AAP are well established and include liver function abnormalities, hypokalemia, and hypertension. These toxicities are manageable with interventions, including proactive laboratory monitoring, dose interruptions, and dose reductions, if needed. Data from sponsor Study 64091742PCR1001 and 64091742PCR3001 (see Section 2.2.3) show that niraparib 200 mg and AAP may be combined safely.[46] This protocol includes a targeted monitoring plan and treatment guidelines to ensure appropriate management of the overlapping toxicities of niraparib and AAP, including hypertension, liver function abnormalities, and hypokalemia (see Section 6.5). In addition, participants are required to have specified laboratory values in the inclusion criteria (see Section 5.1) and uncontrolled hypertension and known active hepatitis B and active hepatitis C are included as exclusion criteria (see Section 5.2). As noted in the SoA, blood pressure, heart rate, potassium, and liver function tests will be monitored through EoT.
- PK data from Study 64091742PCR1001 also show that exposures for both niraparib and AA are comparable to when each drug is administered as a single agent.[46]
- Treatment-emergent AEs and other safety data for the study will be monitored by the Independent Data Monitoring Committee (IDMC) and the sponsor’s medical monitor and internal safety management committee during the conduct of the study, as described throughout this protocol.

- CCI [redacted]

Given that the data suggest that there is a potential for increased efficacy for the combination of niraparib and AA plus prednisone in patients with deleterious germline or somatic HRR gene-mutated metastatic prostate cancer and that the anticipated toxicities of niraparib and AA plus prednisone are recognizable through medical oversight and laboratory monitoring and are able to be managed medically, the sponsor considers that there is a positive benefit/risk profile and strong rationale for evaluating niraparib and AA plus prednisone for the treatment of patients with mCSPC.

Taking into account the measures incorporated to minimize risk to participants of this study, the potential risks identified in association with niraparib/AA FDC, niraparib and AA, plus prednisone are justified by the anticipated benefits that may be afforded to participants with mCSPC.

By-gene analysis from Study 64091742PCR3001 showed no clear evidence of benefit of niraparib combined with AAP in patients with CDK12 alterations.[48] While clinical benefit could not be excluded, given the distinct biology in CDK12-altered prostate cancer, as well as other emerging data suggesting limited efficacy of PARP inhibition in this group of patients, participants with CDK12-only alterations are no longer being enrolled in Study 67652000PCR3002.

More detailed information about the known and expected benefits and risks of niraparib and AA may be found in the niraparib, AA, and niraparib/AA FDC IBs[24,25,26] as well as in the latest version of the package insert for ZEJULA® (niraparib), ZYTIGA® (AA) and prednisone, that is applicable for the local country in which the study is being conducted.

3. OBJECTIVES AND ENDPOINTS

Objectives	Endpoints
**Primary**	
• To determine if niraparib and AA, plus prednisone compared with AA plus prednisone in participants with deleterious germline or somatic HRR gene-mutated mCSPC provides superior efficacy in improving rPFS	• rPFS by investigator (based on PCWG3)
**Secondary**	
• To assess the clinical benefit of niraparib and AA, plus prednisone compared with AA plus prednisone in participants with deleterious germline or somatic HRR gene-mutated mCSPC	• OS[a]<br>• Time to symptomatic progression[b]<br>• Time to subsequent therapy[c]
• To characterize the safety profile of niraparib and AA, plus prednisone compared with AA plus prednisone in participants with deleterious germline or somatic HRR gene-mutated mCSPC	• Incidence and severity of adverse events

Objectives	Endpoints
**Other**	
• To evaluate other efficacy assessments and determine the clinical benefit of niraparib and AA, plus prednisone compared with AA plus prednisone in participants with deleterious germline or somatic HRR gene-mutated mCSPC	• PFS2[d]<br>• Objective response[e]<br>• Time to PSA progression[f]
• To characterize the PK of niraparib when administered as niraparib/AA FDC plus prednisone	• Observed plasma concentrations of niraparib and estimated population PK and exposure parameters for niraparib
• To show the effect of niraparib and AA, plus prednisone is similar to AA plus prednisone on health-related quality of life	• The FACT-P, the EQ-5D-5L, BPI-SF, and the PRO-CTCAE
• To evaluate biomarkers predictive of response	• Objective response, rPFS, and PSA response across deleterious germline or somatic HRR gene alterations

AA=abiraterone acetate; BPI-SF=Brief Pain Inventory Short Form; CDK12=cyclin dependent kinase 12;DR=EQ-5D-5L=Euro Quality of Life questionnaire; FACT-P=Functional Assessment of Cancer Therapy-Prostate; PRO-CTCAE= Patient-reported Outcomes Common Terminology Criteria for Adverse Events; FDC=fixed-dose combination; HRR=homologous recombinant repair; mCSPC=metastatic castration-sensitive prostate cancer; OS=overall survival; PCWG3=Prostate Cancer Working Group 3; PFS2=progression-free survival 2; PK=pharmacokinetic; PSA=prostate-specific antigen; rPFS=radiographic progression-free survival; RECIST=Response Evaluation Criteria in Solid Tumors

a. OS: defined as the time from date of randomization to date of death due to any cause.

b. Time to symptomatic progression is defined as time from the date of randomization to the date of any of the following (whichever occurs first):
- -The use of external beam radiation therapy for skeletal or pelvic symptoms. Note: Only radiation planned prior to randomization will not be considered as symptomatic progression
- -The need for tumor-related orthopedic surgical intervention
- -Other cancer-related procedures (eg, nephrostomy insertion, bladder catheter insertion, external beam radiation therapy, or surgery for tumor symptoms)
- -Cancer-related morbid events (ie, fracture [symptomatic and/or pathologic], cord compression, urinary obstructive events)
- -Initiation of a new systemic anti-cancer therapy because of cancer symptoms.

c. Time to Subsequent Therapy: defined as the time from date of randomization to the date of initiation of subsequent therapy for prostate cancer.

d. PFS2: defined as time from date of randomization to date of first occurrence of disease progression (radiographic, clinical, or PSA progression) on first subsequent therapy for prostate cancer or death, whichever comes first.

e. Objective response: defined as achieving a complete or partial response according to modified RECIST 1.1

f. Time to PSA progression: defined as the time from the date of randomization to the date of PSA progression based on PCWG3 criteria.

Refer to Section 8, Study Assessments and Procedures for evaluations related to endpoints.

HYPOTHESIS

The primary hypothesis of this study is that niraparib and AA, plus prednisone will improve rPFS compared with AA plus prednisone in participants with deleterious germline or somatic HRR gene-mutated mCSPC.

4. STUDY DESIGN

4.1. Overall Design

This is a randomized, placebo-controlled, double-blind, multinational Phase 3 treatment study to evaluate the safety and efficacy of niraparib and AA, plus prednisone compared with AA plus prednisone in men over the age of 18 years with deleterious germline or somatic HRR gene-mutated mCSPC receiving ADT (ie, gonadotropin-releasing hormone analogue [GnRHa; agonist or antagonist] or surgical castration).

Approximately 692 participants will be randomly assigned in a 1:1 ratio to either niraparib 200 mg and AA 1000 mg, plus prednisone 5 mg daily or AA 1000 mg plus prednisone 5 mg daily (Figure 1). All participants will be receiving background ADT therapy, the choice of which is at the discretion of the investigator. Participants will be stratified based on BRCA2 and CDK12 status (BRCA2 alteration present versus CDK12 alteration present versus all other pathogenic alterations), prior docetaxel use (yes versus no), and volume of disease (high versus low). The study will consist of 4 phases: a Prescreening Phase for biomarker evaluation for eligibility only, a Screening Phase, a Treatment Phase, and a Follow-up Phase.

Efficacy evaluations include the following: tumor measurements by computed tomography (CT), magnetic resonance imaging (MRI; abdomen, chest, and pelvis), Technetium-99m (^{99m}Tc) bone scans, serum PSA evaluations, and patient reported outcomes (PROs). Plasma levels of niraparib and its M1 metabolite will be assessed. Safety evaluations include incidence of AEs and clinical laboratory parameters.

Participants receive study medication in 28-day treatment cycles. Study medication should continue until disease progression, unacceptable toxicity, death, withdrawal of consent, or termination of the study by the sponsor (see Section 7.1). Patients with radiographic progression can remain on therapy if still receiving clinical benefit. Imaging will be performed at Day 1 of Cycle 3, Cycle 5, and then every 4 cycles. If the participant has radiographic progression, but not clinical progression, and alternate treatment is not initiated, the participant may continue study medication at the investigator's discretion.

After discontinuing study medication, participants will be contacted every 4 months until death or termination of the study. Participants who discontinue study medication prior to reaching the primary endpoint of rPFS will continue to be scanned until reaching the primary endpoint. In addition to survival follow-up, data will continue to be collected to evaluate all of the secondary and other endpoints. The Euro-Quality of Life Questionnaire (EQ-5D-5L) will also be administered every 4 months for up to 1 year after study medication discontinuation.

Participants will be monitored for safety during the Prescreening, Screening, and Treatment Phases and up to 30 days after the last dose of study medication during the Follow-up Phase. Adverse events, including clinically significant laboratory abnormalities reported as AEs, will be graded and summarized using National Cancer Institute Common Terminology Criteria for Adverse Events (NCI-CTCAE), Version 5. Dose modification guidelines are provided in Section 6.5.

An IDMC will be commissioned for this study and will perform regular review of safety data. Refer to Committees Structure in Section 10.2, Appendix 2, Regulatory, Ethical, and Study Oversight Considerations for details.

A diagram of the study design is provided in Figure 1.

4.2. Scientific Rationale for Study Design

Blinding, Control, Study Phase/Periods, Treatment Groups

An active control will be used in this study. Randomization will be used to minimize bias in the assignment of participants to treatment groups, to increase the likelihood that known and unknown participant attributes (eg, demographic and baseline characteristics) are evenly balanced across treatment groups, and to enhance the validity of statistical comparisons across treatment groups. Blinded treatments will be used to reduce potential bias during data collection and evaluation of clinical endpoints.

The control for this study is AA 1000 mg plus prednisone 5 mg daily.

As treatment options for men with mCSPC include docetaxel, prior docetaxel will be permitted, and prior docetaxel is a stratification factor to ensure balance of this prior therapy between the arms.

Biomarker Collection

Participants enrolled in this study must have demonstrated at least one deleterious germline or somatic HRR gene alteration as listed in Table 4. Adjustments regarding the inclusion of participants with specific deleterious germline or somatic HRR gene alterations may be made based on evidence from emerging data or to ensure enough participants are enrolled to allow for subgroup analysis. Any biomarker-related enrollment changes will be communicated to investigators.

During the Prescreening Phase, participants will be evaluated for HRR gene alterations (see Table 4 for definition of biomarker-positivity). All participants will be required to sign an ICF. After signing the ICF, all participants should have (a) tumor tissue (archival or recently collected), and (b) germline (eg. blood, saliva), and also (c) plasma samples collected (if test is available from the sponsor) (Table 1).

In lieu of prescreening, prior local HRR testing performed in a Clinical Laboratory Improvement Amendment-certified (CLIA) or equivalent laboratory may be used. Sponsor must approve participant's HRR alteration status prior to randomization based on receipt of a redacted biomarker report(s).

The type of samples, testing methodology, and gene alterations used to determine eligibility may be updated by the sponsor, with changes communicated to investigators by letter. All participants will have additional exploratory research samples collected where local regulations allow. Local

tests may be performed using tumor tissue, plasma, or germline sample. Randomized participants that submitted a local result should submit tissue unless the local test result was from the sponsor’s required tissue assay.

For tumor tissue collection, either archival or recently collected tissue is acceptable. If no tumor tissue is available, then the participant may agree to have a new tumor tissue sample collected. If the sample used to determine eligibility fails to produce a conclusive result, then another sample may be assessed.

Table 4: Eligible HRR Gene Alterations

Genes	Definition
BRCA1	Breast Cancer gene 1
BRCA2	Breast Cancer gene 2
BRIP1	BRCA1 Interacting Protein C-terminal Helicase 1 gene
CDK12	Cyclin Dependent Kinase 12
CHEK2	Checkpoint Kinase 2 gene
FANCA	Fanconi Anemia Complementation Group A gene
PALB2	Partner and Localizer of BRCA2 gene
RAD51B	RAD51 paralog B
RAD54L	RAD54-Like

In addition to determining participant eligibility, exploratory research samples will be collected at timepoints outlined in the SoA as local regulations permit and may be evaluated to understand response or resistance in participants, to evaluate surrogates of tumor burden or to further develop diagnostic tests.

Medical Resource Utilization Data Collection

Treatment of mCSPC with niraparib and AA, plus prednisone versus AA plus prednisone may result in a higher number or duration of medical encounters; therefore, comparison will be done across treatment groups.

4.2.1. Participant Input into Design

Qualitative research including 43 patients with metastatic prostate cancer has been performed in order to understand patients’ perceptions of clinical studies in general, study concept, and opportunities for improving study concept and study adherence. A mixed methodology of mini-groups, an on-line community, and web-assisted telephone interviews was used. Patients included clinical study naïve patients (n 35) and clinical study experienced patients (n 8) and were recruited from different regions (US and Canada n 17, Europe n 6, South America n 6, Asia Pacific n 6).

Results included patients’ perception that the study process is burdensome and difficult for them to comply with; they are more willing to accept treatment requirements but have concerns about

pre- and post-treatment phases. Based on this insight, during the follow-up phase, the visits can be performed by phone.

4.2.2. Study-Specific Ethical Design Considerations

The potential benefits of treatment with niraparib and AA, plus prednisone in men with metastatic prostate cancer, both with prospectively selected deleterious germline or somatic HRR gene alterations, outweighs the potential risks involved as described in Section 2.3.

Potential participants will be fully informed of the risks and requirements of the study and, during the study, participants will be given any new information that may affect their decision to continue participation. They will be told that their consent to participate in the study is voluntary and may be withdrawn at any time with no reason given and without penalty or loss of benefits to which they would otherwise be entitled. Only participants who are fully able to understand the risks, benefits, and potential AEs of the study, and provide their consent voluntarily will be enrolled.

All participants will receive full supportive care and will be followed closely for safety and efficacy throughout the study. As rPFS is the primary endpoint, scheduled imaging is incorporated into the protocol. The timing of imaging is designed to capture progression events and allow the clinical investigator to make timely treatment decisions yet balancing this with preventing unnecessary exposure to radiation. The frequency of scanning is consistent with the internationally accepted Response Evaluation Criteria in Solid Tumors (RECIST) 1.1 criteria and Prostate Cancer Working Group 3 (PCWG3) criteria. An IDMC will be commissioned for this study and will perform regular review of safety data. Refer to Committees Structure in Section 10.2, Appendix 2, Regulatory, Ethical, and Study Oversight Considerations for details.

As with all clinical and PK studies, there are risks associated with venipuncture and multiple blood sample collections. To avoid multiple venipunctures, the use of intravenous indwelling catheters is permitted in this study. The blood sample collection scheme will be designed to collect the minimum number of blood samples that can determine the safety, antitumor activity, PK, pharmacodynamics, and biomarker requirements of the study. Note that the total volume of blood to be collected (approximately 1500 mL) is an estimate (see Section 8); the actual amount may vary depending on laboratory standard procedures and the number of days a participant is enrolled in the study. The total blood volume to be collected is considered to be an acceptable amount of blood to be collected over this time period from the population in this study based upon the standard of the WHO guidelines which suggest a standard donation of 450 mL of whole blood and a maximum volume of 3 L donated in any consecutive 12-month period.[56] For more details regarding blood collection, see Section 8.

The results of genetic testing done as part of this study could have implications to family members of the participant. The investigator will arrange for genetic counseling as needed.

4.3. Justification for Dose

The dose of niraparib in this study is 200 mg administered with AA 1000 mg in combination with prednisone 5 mg; all study medications are administered once daily.

As described in Section 2.2.3, in study 64091742PCR1001 niraparib 200 mg was selected to be the optimal dose to be combined with AAP based on the absence of dose-limiting toxicities and an overall tolerable safety profile. No drug-drug interaction has been detected between AAP and niraparib. The safety of niraparib in combination with AAP was further confirmed in the primary analysis of Study 64091742PCR3001.

Based on the above information, a dose of niraparib 200 mg once daily has been selected for this combination study to maximize efficacy, while mitigating the potential for toxicity.

Dose modifications are addressed in Section 6.5.

4.4. End of Study Definition

End of Study Definition

The end of study is considered as the last scheduled study assessment for the last participant in the study, including the Open-label Extension (OLE) and Long-term Extension (LTE) Phases, as applicable. The final data from the study site will be sent to the sponsor (or designee) after completion of the final participant assessment at that study site, in the time frame specified in the Clinical Trial Agreement.

Study Completion Definition

A participant will be considered to have completed the Treatment and Follow-up Phase when a clinical endpoint is reached (death, radiographic progression, clinical progression, or unacceptable toxicity, whichever comes first). All participants will be followed for survival.

5. STUDY POPULATION

Men ≥18 years of age (or the local legal age of consent) with deleterious germline or somatic HRR gene-mutated metastatic prostate cancer (see Section 4.2, Scientific Rationale for Study Design, Biomarker Collection) are eligible for this study. Prescreening activities to determine biomarker status may occur any time prior to the Screening Phase.

The inclusion and exclusion criteria for enrolling participants in this study are described below. If there is a question about these criteria, the investigator must consult with the appropriate sponsor representative and resolve any issues before enrolling a participant in the study. Waivers are not allowed.

For a discussion of the statistical considerations of participant selection, refer to Section 9.2, Sample Size Determination.

Prescreening for Molecular Eligibility

Molecular eligibility will be established for each potential participant before screening for other eligibility criteria (Figure 2).

Figure 2. Determination of Molecular Eligibility

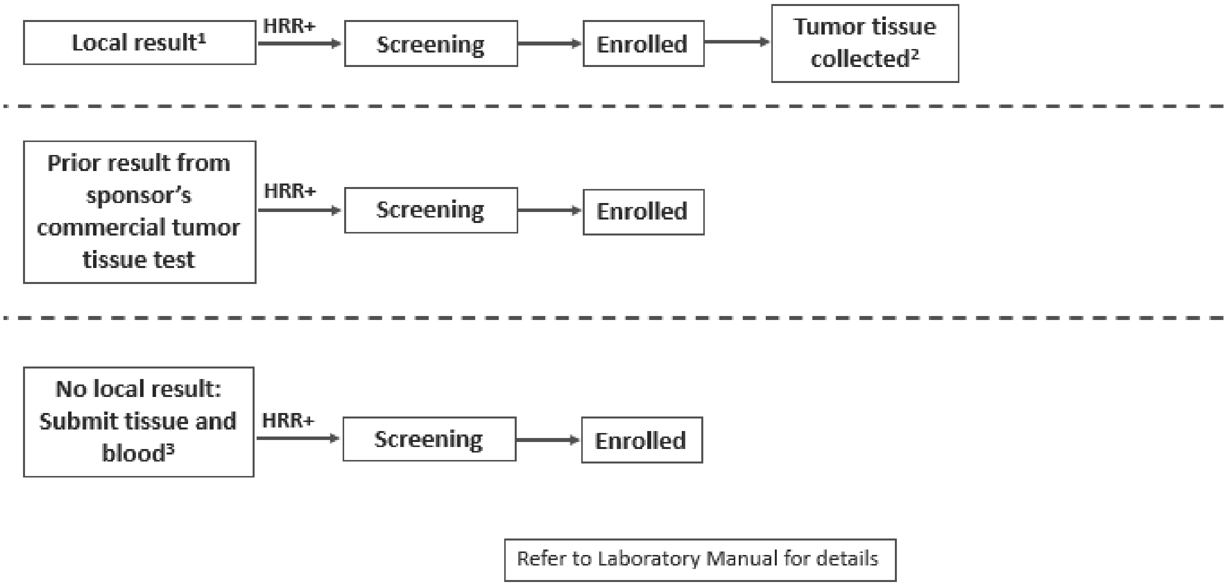

HRR = homologous recombination repair
[1]Local result: Testing performed at study site, commercially, or through another Janssen study in a Clinical Laboratory Improvement Amendment-certified (CLIA) or equivalent laboratory
[2]Tumor tissue (archival or fresh biopsy). Participants that submitted a laboratory result from the sponsor's commercial tumor tissue test do not need to provide tissue.
[3]For circulating tumor DNA (ctDNA) and germline testing, where local regulations permit

Prescreening Eligibility Criteria For Participants Who Do Not Have Prior Results Available from Either a Local Test or Sponsor's Commercial Tumor Tissue Test:

1. Criterion modified per Amendment 3

   1.1 Prescreening informed consent obtained.

2. ≥18 years of age (or the local legal age of consent).

3. Criterion modified per Amendment 2.

   3.1. Diagnosis of metastatic prostate adenocarcinoma.

4. Criterion modified per Amendment 1.

   4.1. Criterion modified per Amendment 2

   4.2. Willing to provide tumor tissue (archival or fresh), blood or saliva, and plasma (if test is available from the sponsor) for determination of deleterious germline or somatic HRR gene alterations (see Table 4), if no local (testing done at investigator center or commercial testing) or prior sponsor-approved test result is available (see Figure 2).

5.1. Inclusion Criteria

Each potential participant must satisfy all of the following criteria to be enrolled in the study:

1. ≥18 years of age (or the local legal age of consent).

2. Criterion modified per Amendment 2.

   2.1. Pathological diagnosis of prostate adenocarcinoma.

3. Criterion deleted per Amendment 1.

4. Criterion modified per Amendment 2.

   4.1. Metastatic disease documented by conventional imaging with CT or MRI (for soft tissue lesions) or 99mTc bone scan (for bone lesions). Participants with a single bone lesion on 99mTc bone scan with no other non-nodal metastatic disease must have confirmation of bone metastasis by CT or MRI.

   a. Participants with lymph node-only disease are not eligible.

5 Criterion modified per Amendment 1.

   5.1. Must have at least one of the deleterious germline or somatic HRR gene alterations listed in Table 4.

6. Eastern Cooperative Oncology Group Performance Status (ECOG PS) Grade ≤2.

7. Androgen deprivation therapy (either medical or surgical castration) must have been started ≥14 days prior to randomization and participants be willing to continue ADT through the treatment phase. Participants who start a GnRH agonist ≤28 days prior to randomization will be required to take a first-generation anti-androgen for ≥14 days prior to randomization. The anti-androgen must be discontinued prior to randomization.

8. Criterion modified per Amendment 1.

   8.1. Criterion modified per Amendment 2.

   8.2. Participants who have received prior docetaxel treatment must meet the following criteria:

   a. Received a maximum of 6 cycles of docetaxel therapy for mCSPC

   b. Received the last dose of docetaxel ≤3 months prior to randomization

   c. Maintained a response to docetaxel of stable disease or better, by investigator assessment of imaging and/or PSA, prior to randomization.

9. Criterion modified per Amendment 1.

   9.1. Criterion modified per Amendment 2.

   9.2. Other allowed prior therapy for mCSPC:

   a. Maximum of 1 course of radiation and 1 surgical intervention for symptomatic control of prostate cancer (eg, uncontrolled pain, impending spinal cord compression or obstructive symptoms). Participants with radiation or surgical interventions to all known sites of metastatic disease will be excluded from trial participation. Radiation must be completed prior to randomization.

   b. Up to a maximum of 6 months of ADT prior to randomization.

   c. Up to a maximum of 45 days of AAP prior to randomization.

   d. Up to a maximum of 2 weeks of ketoconazole for prostate cancer prior to randomization.

10. Criterion modified per Amendment 2.

   10.1. Allowed prior treatments for localized prostate cancer include radical prostatectomy (with or without lymph node dissection), radiation therapy, and other locally directed treatments to the prostate per institutional standards of care.

   a. Participants who received ADT or first-generation anti-androgens for the treatment of localized prostate cancer

      i. ADT: must have had ≤ 3 years total and must have completed ≥1 year prior to randomization

      ii. First-generation anti-androgen: must have had ≤3 years total and must have completed ≥1 year prior to randomization.

11. Criterion modified per Amendment 1.

   11.1. Clinical laboratory values at Screening:

   a. Absolute neutrophil count ≥1.5 x 10^9/L

   b. Hemoglobin ≥9.0 g/dL, independent of transfusions for at least 28 days

   c. Platelet count ≥100 x 10^9/L

   d. Creatinine ≤2 x upper limit of normal (ULN)

   e. Serum potassium ≥3.5 mmol/L

   f. Serum total bilirubin ≤1.5× ULN or direct bilirubin ≤1 x ULN (*Note:* In participants with Gilbert's syndrome, if total bilirubin is >1.5 × ULN, measure direct and indirect bilirubin, and if direct bilirubin is ≤1.5 × ULN, participant may be eligible)

   g. AST or ALT ≤3 × ULN

12. Able to swallow the study medication tablets whole.

13. Criterion modified per Amendment 1.

13.1. Must sign informed consent (written or remote/virtual) indicating that he understands the purpose of, and procedures required for, the study and is willing to participate in the study including providing a DNA sample.

14. Criterion modified per Amendment 1

14.1. While on study medication and for 3 months following the last dose of study medication, a male participant must agree to use an adequate contraception method as deemed appropriate by the investigator and as specified in Section 5.3. Lifestyle Considerations.

15. Criterion modified per Amendment 1

15.1. A male participant must agree not to donate sperm while on study treatment and for a minimum of 3 months following the last dose of study medication.

5.2. Exclusion Criteria

Any potential participant who meets any of the following criteria will be excluded from participating in the study:

1. Pathological finding consistent with small cell or neuroendocrine carcinoma of the prostate.

2. Prior treatment with a PARP inhibitor.

3. Criterion modified per Amendment 1.

3.1. Criterion modified per Amendment 2.

3.2. Prior AR-targeted therapy (eg, apalutamide, enzalutamide, darolutamide), immunotherapy, or radiopharmaceutical agents for prostate cancer with the Exception: allowed prior therapies are noted in inclusion criteria 9.2.

4. Criterion deleted per Amendment 2.

5. History of adrenal dysfunction

6. Long-term use of systemically administered corticosteroids (>5 mg of prednisone or the equivalent) during the study is not allowed. Short-term use (≤4 weeks, including taper) and locally administered steroids (eg, inhaled, topical, ophthalmic, and intra-articular) are allowed, if clinically indicated.

7.
- Active malignancies (ie, progressing or requiring treatment change in the last 24 months) other than the disease being treated under study. The only allowed exceptions are:
  a. non-muscle invasive bladder cancer;
  b. skin cancer (non-melanoma or melanoma) treated within the last 24 months that is considered completely cured;

c. breast cancer adequately treated lobular carcinoma in situ or ductal carcinoma in situ;
d. malignancy that is considered cured with minimal risk of recurrence.

8. History or current diagnosis of MDS/AML.

9. Current evidence within 6 months prior to randomization of any of the following: severe/unstable angina, myocardial infarction, symptomatic congestive heart failure, clinically significant arterial or venous thromboembolic events (ie. pulmonary embolism), or clinically significant ventricular arrhythmias.

10. Presence of sustained uncontrolled hypertension (systolic blood pressure >160 mm Hg or diastolic blood pressure >100 mm Hg). Participants with a history of hypertension are allowed, provided that blood pressure is controlled to within these limits by an anti-hypertensive treatment.

11. Known allergies, hypersensitivity, or intolerance to the excipients of niraparib, AA, or niraparib/AA FDC (refer to the IBs for niraparib and AA).[24,25,26]

12. Current evidence of any medical condition that would make prednisone use contraindicated.

13. Received an investigational intervention (including investigational vaccines) or used an invasive investigational medical device within 30 days before the planned first dose of study medication.

14. Participants who have had the following ≤28 days prior to randomization:

a. A transfusion (platelets or red blood cells);
b. Hematopoietic growth factors;
c. Major surgery (sponsor should be consulted regarding what constitutes major surgery).

15. Criterion was deleted per Amendment 1

16. Human immunodeficiency virus positive participants with 1 or more of the following:

a. Not receiving highly active antiretroviral therapy or on antiretroviral therapy for less than 4 weeks.
b. Receiving antiretroviral therapy that may interfere with the study medication (consult the sponsor for review of medication prior to enrollment).
c. A change in antiretroviral therapy within 6 months of the start of screening (except if, after consultation with the sponsor on exclusion criterion 16.b, a change is made to avoid a potential drug-drug interaction with the study medication).
d. CD4 count <350 at screening.

e. An acquired immunodeficiency syndrome-defining opportunistic infection within 6 months of the start of screening.

f. Human immunodeficiency virus load ≥400 copies/mL.

17. Active or symptomatic viral hepatitis or chronic liver disease; encephalopathy, ascites or bleeding disorders secondary to hepatic dysfunction.

18. Criterion modified per Amendment 2.

18.1. Moderate or severe hepatic impairment (Class B and C per Child-Pugh classification system (Appendix 9).

NOTE: Investigators should ensure that all study enrollment criteria have been met at screening. If a participant's clinical status changes (including any available laboratory results or receipt of additional medical records) after screening but before the first dose of study medication is given such that he no longer meets all eligibility criteria, then the participant should be excluded from participation in the study. Section 5.4, Screen Failures, describes options for retesting. The required source documentation to support meeting the enrollment criteria are noted in Section 10.2, Appendix 2, Regulatory, Ethical, and Study Oversight Considerations.

5.3. Lifestyle Considerations

Potential participants must be willing and able to adhere to the following lifestyle restrictions during the course of the study to be eligible for participation:

1. Refer to Section 6.8 for details regarding prohibited and restricted therapy during the study.

2. Agree to follow all requirements that must be met during the study as noted in the Inclusion and Exclusion Criteria (eg, contraceptive requirements).

3. Must agree to always use a condom during sexual intercourse (even in case of prior vasectomy or in case of intercourse with an already pregnant woman) or to remain abstinent during the study and for 3 months after the last study treatment administration (Section 7.1 Discontinuation of Study Medication).

   If the participant is engaged in sexual activity with a woman of childbearing potential, then a condom should be used along with another highly effective contraceptive method. Highly effective methods of contraception (methods that can achieve a failure rate of less than 1% per year when used consistently and correctly) include:

      combined hormonal (estrogen + progesterone or progesterone only) contraception associated with inhibition of ovulation: oral, injectable or implantable;

placement of an intrauterine device (IUD) or intrauterine hormone releasing system (IUS);

bilateral tubal occlusion;

vasectomy;

sexual abstinence; please note that sexual abstinence is considered a highly effective method only if defined as refraining from heterosexual intercourse during the entire period of risk associated with the study treatments. The reliability of sexual abstinence needs to be evaluated in relation to the duration of the clinical trial and the preferred and usual lifestyle of the participant.

A highly effective contraceptive method should be used for the duration of the study and for 3 months after the last study medication administration.

5.4. Screen Failures

Participant Identification, Enrollment, and Screening Logs

The investigator agrees to complete a participant identification and enrollment log to permit easy identification of each participant during and after the study. This document will be reviewed by the sponsor study-site contact for completeness. This study will use interactive web response system (IWRS). The investigator will generate screening and enrollment logs directly from IWRS.

The participant identification and enrollment log will be treated as confidential and will be filed by the investigator in the study file. To ensure participant confidentiality, no copy will be made. All reports and communications relating to the study will identify participants by participant identification and age at initial informed consent. In cases where the participant is not randomized into the study, the date seen and age at initial informed consent will be used.

Participants who do not meet all inclusion criteria, or who meet an exclusion criterion, may be rescreened once. Rescreening is at the discretion of the investigator. After discussion with the sponsor, rescreened participants may be able to use the initial screening laboratory results as long as safety laboratory evaluations (hematology panel, serum chemistry panel, and liver function tests [LFTs]) described in Section 8.2.4 have been performed within 14 days of randomization. Initial CT/MRI and bone scans may be used to determine eligibility if within 6 weeks of planned randomization. All other rescreening and subsequent randomization activities must be conducted in accordance with all protocol defined windows and timelines. The sponsor or designee will review results from the screening visit prior to randomization to confirm participant selection for the study.

6. STUDY MEDICATION AND CONCOMITANT THERAPY

6.1. Study Medication(s) Administered

Designation	Product
Investigational Medicinal Products	Niraparib<br>AA<br>Niraparib/AA FDC tablets*<br>Matching placebo for the above IMPs.<br>Status in EU: product will be used in accordance with the terms of their marketing authorization.<br>*not authorized for the indication investigated in this study.
Authorized Auxiliary Medicinal Product	Androgen-deprivation therapy: GnRHa such as leuprolide, goserelin, histrelin, triptorelin, degarelix, relugolix (ATC: L02AE and L02BX).<br>Prednisone/prednisolone.<br>Status in the EU: product will be used in accordance with the terms of their marketing authorization.
Authorized Diagnostic Auxiliary Medicinal Product	Tracers used for bone scans such as ^{99m}Tc (ATC: V09I).<br>Status in the EU: product will be used in accordance with the terms of their marketing authorization.

99mTc=technetium-99m; AA=abiraterone acetate; ATC=Anatomical Therapeutic Chemical; EU=European Union; FDC=fixed-dose combination; GnRHa=gonadotropin-releasing hormone analogue; IMP=investigational medicinal product

For the purpose of the study, "study medication" refers to niraparib, AA, Fixed Dose Combination (niraparib+AA), matching placebos and prednisone.

6.1.1. ADT Administration

Continuous treatment with a GnRHa (if not previously surgically castrated) is mandatory for all participants. The choice of GnRHa is at the discretion of the investigator. Dose and dose schedule (without interruption) will be consistent with the prescribing information and should only be adjusted if clinically indicated to maintain castrate concentrations of testosterone. For participants who did not undergo surgical castration, concurrent treatment with a GnRHa must be documented in the electronic case report form (eCRF).

6.1.2. Study Medication

Study medication is administered orally, once daily on an outpatient basis. A treatment cycle is defined as 28 days. Further details regarding how study medications are supplied are described in Table 5, Table 6, and Table 7. Sufficient study medication for each treatment cycle will be distributed on the first day of each cycle. Participants will begin taking study medication on Day 1 of Cycle 1. If a participant forgets to take the study medication, then the missed dose(s) should only be replaced within the same day.

Participants will be randomized to 1 of 2 treatments groups:

- niraparib 200 mg and AA 1000 mg, plus prednisone 5 mg daily (Experimental)
- AA 1000 mg plus prednisone 5 mg daily (Control)

Matching placebos will also be administered.

Participants randomized to receive niraparib and AA plus prednisone will receive 2 niraparib/AA regular-strength FDC tablets (100 mg niraparib/500 mg AA per tablet), 4 AA placebo tablets, plus 5 mg prednisone.

Participants randomized to receive AA plus prednisone will receive placebo corresponding to 2 FDC tablets (containing neither niraparib nor AA), 4 AA tablets (250 mg each), plus 5 mg prednisone.

A low strength FDC tablet containing 50 mg niraparib/500 mg AA is available for participants requiring a dose reduction of niraparib.

If a dose reduction is required that cannot be supplied using a FDC tablet, equivalent single-agent medication will be supplied.

For treatment modifications due to toxicities, see Section 6.5. For dose modification, see single-agent formulations in Table 6 and low strength FDC formulations in Table 7.

For special dosing instructions during PK visits, see Section 6.1.3.

Table 5: Description of Study Medications

Arm Name	Niraparib/AA plus Prednisone			AA plus Prednisone		
**Study Medication Name**	Niraparib/AA FDC	Placebo AA	Prednisone*	Placebo FDC	AA	Prednisone
**Type**	Combination product containing 2 drugs	Drug placebo	Drug	Drug placebo	Drug	Drug
**Dose Formulation**	FDC film coated tablet	tablet	tablet	FDC film coated tablet	tablet	tablet
**Unit Dose Strength(s)**	regular strength FDC 100 mg niraparib/500 mg AA per tablet	250 mg AA placebo	5 mg prednisone per tablet	regular strength FDC placebo	250 mg AA per tablet	5 mg prednisone per tablet
**Dosage Level(s)**	2 tablets of regular strength FDC: 100 mg niraparib/ 500 mg AA (total dose 200 mg niraparib/ 1000 mg AA) once daily	4 tablets of placebo once daily	1 tablet (5 mg) once daily	2 tablets of regular strength FDC placebo once daily	4 tablets (total dose 1000 mg AA) once daily	1 tablet (5 mg) once daily
**Route of Administration**	oral	oral	oral	oral	oral	oral
**Sourcing**	Provided centrally by the sponsor	Provided centrally by the sponsor	Provided locally by the study site, subsidiary, or designee	Provided centrally by the sponsor	Provided centrally by the sponsor	Provided locally by the study site, subsidiary, or designee
**Packaging and Labeling** (Labels will contain information to meet the applicable regulatory requirements)	high density polyethylene bottles with child resistant closure	high density polyethylene bottles with child resistant closure.	as provided locally by the study site, subsidiary, or designee	high density polyethylene bottles with child resistant closure.	high density polyethylene bottles with child resistant closure	as provided locally by the study site, subsidiary, or designee
**Delivery Instructions**	See Section 6.1.3 for special directions for PK visits					
**Food/Fasting Requirement**	Take on an empty stomach. No food should be consumed for at least 2 hours before and for at least 1 hour after dosing for study drug except Prednisone.<br>*Prednisolone may be administered in countries where prednisone is not available. Prednisone/Prednisolone should be taken along with food.					

Table 6: Single-Agent Formulations

Study Medication Name	Niraparib	Placebo for Niraparib	AA	Placebo for AA
**Type**	drug	drug - placebo	drug	drug - placebo
**Dose Formulation**	capsule or tablet*	capsule	tablet	tablet
**Unit Dose Strength**	100 mg per capsule or tablet*	placebo	250 mg per tablet	placebo
**Dosage Level(s)**	100 mg or 200 mg once daily – see Section 6.5	once daily	500 mg or 1000 mg once daily – see Section 6.5	once daily
**Route of Administration**	oral	oral	oral	oral
**Sourcing**	Provided centrally by the sponsor	Provided centrally by the sponsor	Provided centrally by the sponsor	Provided centrally by the sponsor
**Packaging and Labeling** (Labels will contain information to meet the applicable regulatory requirements)	high-density polyethylene bottles with child-resistant closure			
**Food/Fasting Requirement**	Take on an empty stomach. No food should be consumed for at least 2 hours before and for at least 1 hour after dosing			

Note: In the event the FDC formulation is not available, single agent formulations will be used.
*Niraparib tablet may only be used during the Open label Extension/Long term Extension Phase after regulatory approval in the jurisdiction as applicable.

Table 7: Low-strength niraparib FDC Formulations for Use When Dose Modification is Required and When Available

Study Medication Name	Low-strength niraparib/AA FDC	Low-strength placebo FDC
**Type**	Combination product containing 2 drugs	Drug - placebo
**Dose Formulation**	FDC film-coated tablet	FDC film-coated tablet
**Unit Dose Strength(s)**	low-strength FDC: 50 mg niraparib/500 mg AA per tablet	low-strength FDC: placebo
**Dosage Level(s)**	2 tablets of low-strength FDC 50 mg niraparib/500 mg AA FDC (total dose = 100 mg niraparib/1000 mg AA) once daily	2 tablets of low-strength FDC placebo once daily
**Route of Administration**	oral	oral
**Sourcing**	Provided centrally by the sponsor	Provided centrally by the sponsor
**Packaging and Labeling** (Labels will contain information to meet the applicable regulatory requirements)	high-density polyethylene bottles with child resistant closure	high-density polyethylene bottles with child resistant closure
**Delivery instructions**	See Section 6.1.3 for special directions for PK visits	See Section 6.1.3 for special directions for PK visits
**Food/Fasting Requirement**	Take on an empty stomach. No food should be consumed for at least 2 hours before and for at least 1 hour after dosing.	Take on an empty stomach. No food should be consumed for at least 2 hours before and for at least 1 hour after dosing.

Study medication administration must be captured in the source documents and the eCRF. Study-site personnel will instruct participants on how to store study medication for at-home use as indicated for this protocol.

Niraparib, AA, placebo niraparib, and placebo AA will be manufactured and provided under the responsibility of the sponsor. Refer to the IBs for a list of excipients for niraparib and AA.[24,25,26]

Regular-strength niraparib/AA FDC and low-strength niraparib/AA FDC, as well as placebos for these products, will be manufactured and provided under the responsibility of the sponsor. The FDC drug product formulations will be manufactured as film-coated 100 mg niraparib/500 mg AA and 50 mg niraparib/500 mg AA tablets for oral administration containing 159.40 or 79.70 mg of niraparib tosylate monohydrate drug substance, respectively, equivalent to 100 or 50 mg niraparib free base, and 500 mg of AA drug substance. Refer to the IB for a list of excipients for niraparib/AA FDC.[24]

For a definition of study medication overdose, refer to Section 6.7, Treatment of Overdose.

6.1.3. Special Dosing Instructions for Pharmacokinetic Visits

For PK sampling on Day 1 of Cycles 2 and 3, study medications should be taken at the site under the supervision of the investigator or designee and should not be taken at home on the morning of the visits; PK samples are to be obtained predose and 1-3 hours postdose. If the participant takes the study medications prior to the visit, the PK samples should be obtained at the next cycle. On Day 1 of Cycles 4 through 7, one PK sample is to be obtained either predose or at least 3 hours postdose. If the study visit is in the morning, participants should refrain from taking the study medications until after the PK sample is obtained at the investigative site. If the visit is in the afternoon, the participant should take their study medications in the morning as usual, and the PK sample will be collected during the study visit. If a participant's study medication is interrupted (eg, for toxicity), the site should contact the sponsor for instructions regarding PK sample collection. Details of PK sampling days and times are provided in the SoA Table 3.

Additional details regarding PK sampling are provided in Section 8.4. Details of blood sample handling and storage procedures for PK are provided in the laboratory manual.

6.2. Preparation/Handling/Storage/Accountability

Preparation/Handling/Storage

Niraparib could cause embryonic or fetal harm, including embryo-lethality and teratogenic effects. Caregivers should not handle niraparib without gloves. Abiraterone acetate is contraindicated in women who are or may potentially be pregnant. There are no human data on the use of AA in pregnancy. Women who are pregnant or may be pregnant should not handle AA without protection (eg, gloves). Caregivers should also not handle niraparib/AA FDC tablets without gloves.

It is not known whether niraparib or its metabolites are present in or have transient effects on the composition of semen. It is not known whether abiraterone or its metabolites are present in semen. Therefore, to avoid risk of drug exposure for study medications through the ejaculate (even

participants with vasectomies), participants must use a condom during sexual activity while on study medication and for 3 months following the last dose of study medication. Donation of sperm is not allowed while on study medication and for 3 months following the last dose of study medication.

Refer to the pharmacy manual/study site investigational product and procedures manual for additional guidance on study medication preparation, handling, and storage.

Accountability

The investigator is responsible for ensuring that all study medication received at the site is inventoried and accounted for throughout the study. The dispensing of study medication to the participant, and the return of study medication from the participant (if applicable), must be documented. Participants, or their legally acceptable representatives where applicable, must be instructed to return all original containers, whether empty or containing study medication. All study medication will be stored and disposed of according to the sponsor's instructions. Study-site personnel must not combine contents of the study medication containers.

Study medications must be handled in strict accordance with the protocol and the container label and must be stored at the study site in a limited-access area or in a locked cabinet under appropriate environmental conditions. Unused study medication, and study medication returned by the participant, must be available for verification by the sponsor's study site monitor during on-site monitoring visits. The return to the sponsor of unused study medication, or used returned study medication for destruction, will be documented. When the study site is an authorized destruction unit and study medication supplies are destroyed on-site, this must also be documented.

Potentially hazardous materials containing hazardous liquids, should be disposed of immediately in a safe manner and therefore will not be retained for accountability purposes.

Study medications should be dispensed under the supervision of the investigator or a qualified member of the study-site personnel, or by a hospital/clinic pharmacist. Study medications will be supplied only to participants participating in the study. Returned study medication must not be dispensed again, even to the same participant. Whenever a participant brings his or her study medications to the study site for pill count, this is not seen as a return of supplies. Study medications may not be relabeled or reassigned for use by other participants. The investigator agrees neither to dispense the study medication from, nor store it at, any site other than the study sites agreed upon with the sponsor. Further guidance and information for the final disposition of unused study medications are provided in the Investigational Product and Procedures Manual.

6.3. Measures to Minimize Bias: Randomization and Blinding

Study Medication Allocation

Procedures for Randomization and Stratification

Central randomization will be implemented in this study. Participants will be randomly assigned to 1 of 2 treatment groups based on a computer-generated randomization schedule prepared by the

sponsor. The randomization will be balanced by using randomly permuted blocks and will be stratified based on 3 factors: HRR gene alterations (BRCA2 alteration present versus CDK12 alteration present versus all other pathogenic alterations), prior docetaxel use (yes versus no), and volume of disease at screening (high versus low). High-volume mCSPC is based on an adaptation of the CHAARTED study and is defined as 1) visceral metastases or 2) at least 4 bone lesions, with at least 1 bone lesion outside of the vertebral column or pelvis.[55] Low-volume mCSPC is defined as the presence of bone lesion(s) not meeting the definition of high-volume mCSPC.

The IWRS will assign a unique intervention code, which will dictate the intervention assignment and matching study medication kit for the participant. The requestor must use his or her own user identification and personal identification number when contacting the IWRS and will then give the relevant participant details to uniquely identify the participant.

Blinding

The investigator will not be provided with randomization codes. The codes will be maintained within the IWRS, which has the functionality to allow the investigator to break the blind for an individual participant.

Under normal circumstances, the blind should not be broken until completion of the study or the IDMC recommendation for unblinding is accepted by the sponsor and the database is finalized. The investigator may, in a medical emergency, determine the identity of the study medication by contacting the IWRS. While the responsibility to break the study medication code in emergency situations resides solely with the investigator, it is recommended that the investigator contact the sponsor or its designee, if possible, to discuss the particular situation, before breaking the blind. Telephone contact with the sponsor or its designee will be available 24 hours per day, 7 days per week. In the event the blind is broken, the sponsor must be informed as soon as possible. The date and reason for the unblinding must be documented by the IWRS and in the source document. The documentation received from the IWRS indicating the code break must be retained with the participant's source documents in a secure manner. Participants who have had their treatment assignment unblinded must discontinue study medication, have an end-of-treatment (EoT) visit, and continue with follow-up evaluations.

6.4. Study Medication Compliance

A count of all study medications provided by the sponsor will be conducted during the Treatment Phase of this study. Study medications will be dispensed, and dosing compliance will be assessed at study visits as described in the SoA. If there are recurrent issues with dosing compliance in the absence of toxicity, the investigator or designated study-site personnel should re-instruct participants regarding proper dosing procedures.

The study site must maintain accurate records demonstrating dates and amount of study medication received, to whom dispensed (participant-by-participant accounting), and accounts of any study medication accidentally or deliberately destroyed. The amount of study medication dispensed will be recorded and compared with the amount returned. At the end of the study, reconciliation must

be made between the amount of study medication supplied, dispensed, and subsequently destroyed or returned to the sponsor or its representative (see also Section 6.2).

6.5. Dose Modification

Any dose/dosage adjustment should be overseen by medically qualified study-site personnel (principal or sub-investigator unless an immediate safety risk appears to be present).

All dose interruptions and dose reductions (including missed doses) and the reason for the interruption/reduction are to be recorded in the eCRF. Note that cycle days are fixed based on the Cycle 1 Day 1 date and will not change due to dose interruptions or delays. Management of toxicities should be performed as detailed in Table 9.

Once the dose of study medication is reduced, any re-escalation must be discussed in advance with the sponsor's medical monitor.

In this section, the terms niraparib and AA refer to FDC (regular or low strength), single-agents (niraparib and AA) or relevant placebos.

In general, dose interruptions/modifications should be managed as follows:

- All dose modifications documented in the eCRF should have an associated IWRS transaction to ensure participants are dispensed appropriate study medications per treatment assignment.
- The dose of prednisone can remain unchanged with dose modifications of niraparib and AA.
- If niraparib is interrupted or permanently discontinued due to toxicity, then AA may be continued. If AA is interrupted or permanently discontinued due to toxicity, then niraparib may be continued. If AA is permanently discontinued, prednisone should also be discontinued (with a taper if clinically indicated).
- Niraparib must be discontinued for non-hematologic treatment-related Grade ≥3 toxicities lasting more than 28 days while the participant is administered niraparib 100 mg once daily.

General guidelines for dose modifications are provided here (Table 8), in the pharmacy manual, and IWRS instruction manual.

Table 8: Guidelines for Dose Modification

General Dose Reduction Guidelines for Participants on the FDC Treatment Regimen

Dose Required	Dose of Niraparib	Dose of AA	Instruction
Full dose	200 mg	1000 mg	Participants will receive regular-strength FDC tablets (active or placebo) and AA tablets (active or placebo).
Reduced niraparib and full dose AA	100 mg	1000 mg	Participants will receive low-strength FDC (active or placebo) and AA tablets (active or placebo).
Full dose niraparib and reduced AA	200mg	500 mg	Participants will receive a single-agent combination of niraparib (active or placebo) and AA.
Reduced both niraparib and AA	100 mg	500 mg	Participants will receive one regular-strength FDC tablet (active or placebo) and two AA tablets (active or placebo).

Dose Required	Dose of Niraparib	Dose of AA	Instruction
AA only		500 or 1000 mg	Participants will receive single-agent AA if niraparib (active or placebo) is interrupted or discontinued.
Niraparib only	100 or 200mg		Participants will receive single-agent niraparib (active or placebo) if AA is interrupted or discontinued.

AA = abiraterone acetate; FDC = fixed-dose combination

- Non-hematologic Toxicities

For participants who develop drug-related Grade 3 or higher toxicities, treatment with either niraparib or AA (or both if applicable) should be interrupted, unless appropriately controlled per institutional standard. Treatment with study drug(s) must not be reinitiated until symptoms of the toxicity have resolved to Grade 1 or baseline or controlled per institutional standard. If the toxicity cannot be definitively attributed to either niraparib or AA only, then both niraparib/placebo and AA should be interrupted. In cases where both study drugs have been interrupted, both niraparib and AA may be resumed upon resolution of the toxicity (to Grade 1, baseline, or controlled per institutional standard of care). When an ongoing adverse event is attributed to only one of the study medications, it is permissible to resume one study medication while continuing to hold the other (eg, resume niraparib and hold AA or resume AA and hold niraparib.).

Participants who are reported with treatment-related Grade 4 hypertensive crisis will have both study drugs (niraparib and AA) permanently discontinued.

If dose reduction is used for AE management, then please note the following:

Only 1 dose-level reduction will be permitted for niraparib (from 200 mg to 100 mg). Note that if a participant was on a reduced dose of niraparib (100 mg), niraparib must be discontinued for non-hematologic niraparib related Grade ≥3 toxicities lasting continuously for more than 28 days.

Only 1 dose-level reduction will be permitted for AA (from 1000 mg to 500mg).

Table 9: Guidelines for Dose Modification on Specific Adverse events

Adverse Event	Grade	Action
**Guidelines on Specific Adverse Events**		
**Hepatic Toxicities**		
⬆ AST, ALT, or bilirubin	1-2	• No change in niraparib or AA<br>• For grade 2, monitor AST/ALT/bilirubin at least weekly until levels return to grade 1 or baseline.
⬆ AST, ALT, or bilirubin	3	• Interrupt niraparib and AA<br>• Monitor AST/ALT bilirubin at least weekly until AST and ALT return to grade 1 or baseline and total bilirubin <2.0 x ULN then:<br>• Resume AA at a reduced dose of 500 mg and niraparib at 200 mg after discussion and agreement with medical monitor<br>• Refer to Table 8 to determine the correct IWRS dispensation for niraparib and AA
⬆ AST, ALT, or bilirubin	Recurrence Grade 3	• Interrupt niraparib and AA<br>• Monitor AST/ALT/bilirubin at least weekly until AST and ALT return to baseline or grade 1 and total bilirubin ≤2.0 x ULN then:

Adverse Event	Grade	Action
		• AA must be permanently discontinued if already dose reduced to 500 mg<br>• Discussion with medical monitor required if considering resuming niraparib at 100 mg
⬆ AST, ALT, or bilirubin	4	• Interrupt niraparib and AA<br>• AA must be interrupted and discussed with medical monitor.<br>• If AST or ALT is ≥20xULN or bilirubin is ≥10 x ULN, discontinue and do not re-treat with AA<br>• Niraparib must be interrupted. Discussion with medical monitor required if considering resuming niraparib at 100 mg once all values return to baseline
Participants who develop a concurrent elevation of ALT >3 x ULN and a total bilirubin >2 x ULN in the absence of biliary obstruction or other causes responsible for the concurrent elevation should permanently discontinue study medications.		
Notes:<br>During dose interruptions, LFTs should be closely monitored per institutional guidelines (weekly or more frequently as clinically indicated). For participants being retreated, LFTs should be monitored at a minimum of every 2 weeks for 3 months, monthly for the next 3 months and then follow SoA.		
**Hematological Toxicities**		
Decreased platelet and/or neutrophil counts	1	• No change, consider weekly monitoring.
Decreased platelet and/or neutrophil counts	2	• At least weekly monitoring.<br>• Consider interrupting niraparib. If niraparib is interrupted:<br>• Resume niraparib at same dose once grade ≤1 or baseline<br>• Refer to Table 8 to determine the correct IWRS dispensation for AA<br>• Continue single-agent AA.<br>• Weekly monitoring is recommended for 28 days after restarting niraparib.
Decreased platelet and/or neutrophil counts	≥3	• Interrupt niraparib and continue single-agent AA<br>• Monitor blood counts weekly until grade ≤1 or baseline, then resume study medication with following modifications:<br>• Niraparib may be resumed at full dose (200 mg) or reduced dose (100 mg) at the investigator's discretion<br>• AA should be continued at the same dose<br>• Refer to Table 8 to determine the correct IWRS dispensation for niraparib and AA<br>• If participant was already on 100 mg niraparib due to non-hematologic toxicity, discuss with medical monitor prior to resuming.<br>• Weekly monitoring is recommended for 28 days after restarting niraparib.
Decreased platelet and/or neutrophil counts	Second Occurrence Grade ≥3	• Follow above guidelines for dose interruption and monitoring for grade 3 toxicity<br>• Resume study medications with the following modifications:<br>• Resume niraparib at reduced dose (100 mg)<br>• AA should be continued at the same dose<br>• Refer to Table 8 to determine the correct IWRS dispensation for niraparib and AA<br>• If a participant was already receiving 100 mg niraparib, discuss with medical monitor prior to resuming.<br>• Weekly monitoring is recommended for 28 days after restarting dose.

<table>
<tr><th>Adverse Event</th><th>Grade</th><th>Action</th></tr>
<tr><td>Decreased platelet and/or neutrophil counts</td><td>Third Occurrence Grade ≥3</td><td>• Interrupt niraparib and continue single-agent AA
• Monitor levels weekly until Grade ≤1 or baseline
• Permanently discontinue niraparib and continue single-agent AA if neutrophils and/or platelets do not return to Grade 1 or baseline within 28 days of dose interruption and already on 100 mg niraparib.</td></tr>
<tr><td colspan="3">Notes:
• For participants with a platelet count ≤10,000 cells/µL, platelet transfusion per institutional standard of care may be considered. For participants taking anti-coagulant or anti-platelet therapy, consider the risk/benefit of interrupting these drugs or transfusion at an alternative threshold per institutional guidelines or standard of care (eg. ≤20,000 cells/µL).
• Weekly monitoring and/or niraparib interruption are not required if the adverse event is at baseline or lower grade.
• If a participant requires platelet transfusion, has neutropenic fever, or neutropenia requiring granulocyte-colony stimulating factor for a Grade ≥3 AE deemed to be related to niraparib, interrupt niraparib and restart at reduced dose (100 mg) after resolution to grade 1 or baseline. Continue AA at same dose. If the participant was previously dose-reduced for the same hematologic toxicity, permanently discontinue niraparib.
• For the management of anemia, supportive measures such as blood transfusions may be performed as deemed necessary by the investigator per institutional standard of care. Weekly monitoring and/or drug interruptions for anemia are not required for screening hemoglobin values that are Grade 1 or 2. For grade ≥3 anemia, niraparib should be interrupted until resolution to Grade <3. Continue AA at the same dose.
• The site should contact the medical monitor for discussion and consider discontinuation of niraparib (continue single-agent AA) if:
    o Hematologic toxicity has not recovered to Grade 1 or baseline after prolonged period of dose interruption.
        ▪ Niraparib must be discontinued in case of inadequate recovery of platelet counts and neutrophil counts to acceptable levels (Grade 1 or baseline) within 28 days of the dose interruption period, and if the participant has already undergone dose reduction to 100 mg.</td></tr>
<tr><td colspan="3">During the study, participants who receive a diagnosis of MDS/AML, confirmed by a hematologist or oncologist, must permanently discontinue niraparib (See Section 8.2.4).</td></tr>
</table>

Adverse Event	Grade	Action
**Hypokalemia Guidelines[1, 2]**		
Hypokalemia (<LLN – 3 mmol/L to symptomatic with ≤LLN – 3 mmol/L)	1-2	• Maintenance of the participant's potassium level at ≥4.0 mmol should be considered.<br>• If hypokalemia persists despite optimal potassium supplementation and adequate oral intake, the dose of prednisone may be increased by 5mg/day and documented in the study medication eCRF.
Hypokalemia (<3.0 mmol/L)	3-4	• Interrupt AA and dispense single-agent niraparib only<br>• Provide appropriate medical management (eg, obtain electrocardiogram and provide potassium supplement).<br>• Monitor potassium until resolution to Grade 1 or baseline then resume study medications with the following modifications:<br>◦ AA may be resumed at full dose (1000 mg) or dose reduced (500 mg) at investigator's discretion<br>◦ Niraparib should be continued at the same dose<br>◦ Refer to Table 8 to determine the correct IWRS dispensation for niraparib and AA<br>• If hypokalemia persists despite optimal potassium supplementation and adequate oral intake, the dose of prednisone may be increased by 5mg/day to manage refractory mineralocorticoid related toxicities and should be documented in the study medication eCRF.<br>• Hypokalemia may also be associated with niraparib. If hypokalemia does not resolve with an AA dose reduction, consider niraparib dose reduction.<br>• For Grade 3-4 hypokalemia events, consider hospitalization of the participant at the investigator's discretion, based on institutional guidelines or standard of care.

AA=abiraterone acetate; AE=adverse event; ALT=alanine aminotransferase; AML=acute myeloid leukemia; AST=aspartate aminotransferase; LFT=liver function test; LLN=lower limit of normal; MDS= myelodysplastic syndrome; ULN=upper limit of normal; WNL=within normal limits

1 Concomitant medications should be reviewed for potential etiologies of hypokalemia

2 Prednisone should not be interrupted unless previously discussed with medical monitor

6.5.1. Posterior Reversible Encephalopathy Syndrome (PRES)

Participants who develop neurological symptoms suggestive for PRES should be immediately referred for neurological assessment and intake of niraparib interrupted. If diagnosis of PRES is confirmed, treatment with FDC/placebo (or niraparib/placebo, if applicable) must be permanently discontinued. Patients can remain on study and continue to receive AAP.

6.6. Continued Access to Study Medication After the End of the Study

In the event of early completion or study termination by the sponsor, participants who are clinically benefitting from the study medication will be provided with study medication until disease progression, unacceptable toxicity, or an alternate method is in place to avoid treatment interruption. During this time, an abbreviated schedule of study-related procedures will be implemented and limited data collected; details will be provided at a later date.

Telephone contact may be made to determine survival status, subsequent therapy, and disease status progression on subsequent therapy every 4 months until death after the last dose of study

medication, unless the participant has died, is lost to follow-up, has withdrawn consent or the study is terminated by the sponsor. If the information on survival status, subsequent therapy, and disease status progression on subsequent therapy is obtained via telephone contact, written documentation of the communication must be available for review in the source documents. If the participant has died, the date and cause of death will be collected and documented in the eCRF.

Investigators may recontact the participant to obtain long-term follow-up information regarding the participant's safety or survival status including endpoint status as noted in the informed consent form (ICF; refer to Informed Consent in Section 10.2, Appendix 2, Regulatory, Ethical, and Study Oversight Considerations).

6.7. Treatment of Overdose

Overdose is addressed in the niraparib and AA IBs[26,25,26] and in the prednisone product information as follows:

- Niraparib: There is no specific treatment in the event of niraparib overdose. Physicians should follow general supportive measures, interrupt niraparib, and treat symptomatically.
- AA: In the event of an overdose, interrupt AA, undertake general supportive measures, including monitoring for arrhythmias and cardiac failure as well as assess liver functions.
- Prednisone: Treatment of acute overdose (ie, ingestion of large quantities of prednisone over a very short period of time) is immediate gastric lavage or emesis followed by supportive and symptomatic therapy.

In the event of an overdose, the investigator or treating physician should:

- Closely monitor the participant for AE/SAEs and laboratory abnormalities and record in the eCRF. In the event an AE/SAE or laboratory abnormality is observed, continue monitoring with implementation of supportive care measures as clinically indicated until resolution or return to baseline.
- Document the quantity of the excess dose, as well as the duration of the overdose, in the eCRF.
- Contact the Medical Monitor prior to re-starting study drug.

6.8. Concomitant Therapy

Prestudy and concomitant therapies must be recorded throughout the study beginning from providing informed consent up to 30 days after the last dose of study medication.

All therapies (prescription or over-the-counter medications, including vaccines, vitamins, herbal supplements; non-pharmacologic therapies such as electrical stimulation, acupuncture, special diets, exercise regimens, or other specific categories of interest) different from the study medication must be recorded in the eCRF.

6.8.1. Permitted Supportive Care and Interventions

Supportive care medications and interventions are permitted following institutional guidelines. The following supportive care therapies are considered permissible during the study:

- Surgical interventions and procedures such as transurethral resection of the prostate (TURP) and placement of ureteral stents or local radiation therapy for the management of complications due to local progression.
- Transfusions and hematopoietic growth factors per institutional practice guidelines (Note that blood product support and growth factor support are not allowed in the period ≤28 days prior to randomization.).
- Prednisone dose increase by 5mg/day is permitted to manage refractory mineralocorticoid related toxicities and should be documented in the study medication eCRF.

6.8.2. Prohibited Concomitant Therapies

The sponsor must be notified in advance (or as soon as possible thereafter) of any instances in which the following prohibited therapies are administered. If the permissibility of a specific drug/treatment is in question, please contact the sponsor.

- Investigational agents other than the study medications
- Other anti-cancer therapy (including, but not limited to immunotherapy and chemotherapy)
- Other systemic agents that target the androgen axis (eg, anti-androgens, CYP17 inhibitors), except for continued GnRHa.
- Long-term use of systemically administered corticosteroids (>5 mg prednisone or the equivalent) during the study is not allowed. Short-term use (≤4 weeks, including taper) and locally administered steroids (eg, inhaled, topical, ophthalmic, and intra-articular) are allowed, if clinically indicated.
- Testosterone
- Diethylstilbestrol (DES) or similar estrogen receptor agonists
- Spironolactone
- Radiopharmaceuticals such as radium-223 (^{223}Ra), strontium (^{89}Sr), or samarium (^{153}Sm)
- Strong inducers of CYP3A4 (eg, rifampin)

6.8.3. Restricted Concomitant Medications

- Restrictions based on the drug interaction potential with AAP are as follows:
- Substrates of CYP2D6: caution is advised when AA is administered with medicinal products activated by or metabolized by CYP2D6, particularly with medicinal products that have a narrow therapeutic index. Dose reduction of medicinal products with a narrow therapeutic index that are metabolized by CYP2D6 should be considered. Examples of medicinal products metabolized by CYP2D6 include metoprolol, propranolol, desipramine, venlafaxine, haloperidol, risperidone, propafenone, flecainide, codeine, oxycodone, and tramadol (the latter 3 products requiring CYP2D6 to form their active analgesic metabolites).
- Substrates of CYP2C8: In a CYP2C8 drug-drug interaction study in healthy subjects, the AUC of pioglitazone was increased by 46% when pioglitazone was administered with a single dose of 1000 mg AA. Although these results indicate that no clinically meaningful increases in exposure are expected when AA is combined with drugs that are predominantly eliminated by

CYP2C8, participants should be monitored for signs of toxicity related to a CYP2C8 substrate with a narrow therapeutic index (eg, paclitaxel) if used concomitantly with AA.

For the most current information regarding potential drug-drug interactions with niraparib and AA, refer to the latest versions of the IBs for niraparib, and AA.[26,25,26] Additional information is provided in Section 10.3, Appendix 3, Additional Information on CYP450 Drug Interactions.

7. DISCONTINUATION OF STUDY MEDICATION AND PARTICIPANT DISCONTINUATION/WITHDRAWAL

7.1. Discontinuation of Study Medication

Participants should remain on study medication until investigator-assessed radiographic progression. Local symptoms (ie, rising PSA, need for catheter placement, pelvic radiation, etc.) are not reasons for discontinuation of study medication. If a participant's study medication must be discontinued before disease progression, the participant will continue on the study and will continue to be scanned until development of radiographic progression. If the participant has developed radiographic progression but in the opinion of the investigator is continuing to receive benefit from therapy, the participant may continue on study medication.

A participant's study medication must be discontinued if the investigator believes that for safety reasons or tolerability reasons (eg, AE) it is in the best interest of the participant to discontinue study medication (also refer to Section 6.5). Study medication may also be discontinued if the study is terminated by the sponsor.

Study medication should be continued for participants until radiographic or clinical progression. PSA values are not considered a reliable measure of disease progression and should not be used as an indication to discontinue study medication.[47]

A participant may choose at any time to withdraw from the study medication and remain on the study for further follow-up. All attempts should be made to collect data from the follow-up phase such as imaging (if appropriate), survival status, electronic PROs, and any initiation of subsequent therapies (see SoA).

7.2. Participant Discontinuation/Withdrawal From the Study

A participant will be withdrawn from the study for any of the following reasons:

- Lost to follow-up
- Withdrawal of participant consent for subsequent data collection
- Study is terminated by the sponsor

When a participant withdraws from the study medication and procedures before study completion, the exact wishes of the participant with respect to what follow-up is allowed (eg, permission to contact the participant by phone for survival status, permission to access medical records from other providers, etc.) should be documented. When a participant withdraws all consent for any and all further follow-up before study completion, the reason for withdrawal is to be documented in

the eCRF and in the source document. If the reason for withdrawal from the study is withdrawal of consent, then no additional assessments are allowed.

If a participant is lost to follow-up, effort must be made by the study-site personnel to contact the participant and determine endpoint status and the reason for discontinuation/withdrawal. The measures taken to follow-up must be documented. The informed consent will stipulate that even if a participant decides to discontinue the study medications, he will agree to be contacted periodically by the investigator to assess endpoint status. This can be done by telephone or by chart review. If the participant withdraws consent for all study-related procedures, then no further contact is permitted by the investigator or the sponsor. Participant's survival status may be obtained by public record or cancer registry where permitted by local regulations.

Withdrawal of Consent

A participant declining to return for scheduled visits does not necessarily constitute withdrawal of consent. Alternate follow-up mechanisms that the participant agreed to when providing informed consent apply (eg, consult with family members, contacting the participant's other physicians, medical records, database searches, use of locator agencies at study completion) as local regulations permit.

Prior to a participant withdrawing consent for follow-up, the investigator should offer the participant an opportunity for one of the alternative reduced follow-up mechanisms described below. Withdrawal of consent should be an infrequent occurrence in clinical studies,[42] therefore, prior to the start of the study the sponsor and the investigator should discuss and reach a clear understanding of what constitutes withdrawal of consent in the context of the available reduced follow-up mechanisms listed.

Circumstances for Reduced Follow-up

In the situation where a participant may be at risk for withdrawal of consent and is unable to return for scheduled visits at the protocol-defined frequency, the investigator may consider options for reduced follow-up. These may include (as local regulations permit):

- Less frequent clinical visits
- Telephone, email, letter, social media, fax, or other contact with:
    - participant
    - relatives of the participant
    - participant's physicians (general or specialist)
- Review of any available medical records

Details regarding these contacts must be properly documented in source records including responses by participants.

7.2.1. Withdrawal From the Use of Research Samples

A participant who withdraws from the study will have the following options regarding the analysis of research samples:

- The collected samples will be retained and used in accordance with the participant's original separate informed consent for research samples.
- The participant may withdraw consent for analysis of research samples, in which case the samples will be destroyed and no further testing will take place. To initiate the sample destruction process, the investigator must notify the sponsor study site contact of withdrawal of consent for the research samples and to request sample destruction. The sponsor study site contact will, in turn, contact the biomarker representative to execute sample destruction. If requested, the investigator will receive written confirmation from the sponsor that the samples have been destroyed.

Withdrawal From the Use of Samples in Future Research

The participant may withdraw consent for use of samples for future research (refer to Section 10.2, Long-Term Retention of Samples for Future Research). In such a case, samples will be destroyed after they are no longer needed for the clinical study. Details of the sample retention for research are presented in the ICF.

7.3. Lost to Follow-up

To reduce the chances of a participant being deemed lost to follow-up, prior to randomization attempts should be made to obtain contact information from each participant, eg, home, work, and mobile telephone numbers and email addresses for both the participant as well as appropriate family members.

A participant will be considered lost to follow-up if he repeatedly fails to return for scheduled visits and is unable to be contacted by the study site. A participant cannot be deemed lost to follow-up until all reasonable efforts made by the study-site personnel to contact the participant are deemed futile. The following actions must be taken if a participant fails to return to the study site for a required study visit:

- The study-site personnel must attempt to contact the participant to reschedule the missed visit as soon as possible, to counsel the participant on the importance of maintaining the assigned visit schedule, to ascertain whether the participant wishes to or should continue in the study.
- Before a participant is deemed lost to follow-up, the investigator or designee must make every reasonable effort to regain contact with the participant (where possible, 3 telephone calls, e-mails, fax, and, if necessary, a certified letter to the participant’s last known mailing address, or local equivalent methods. Locator agencies may also be used as local regulations permit. These contact attempts should be documented in the participant’s medical records.
- Should the participant continue to be unreachable, they will be considered to have withdrawn from the study.
- Should a study site close, it is expected that the investigator will work with the sponsor to transfer the participant to another study site.

8. STUDY ASSESSMENTS AND PROCEDURES

Overview

The SoA (Table 2) summarizes the frequency and timing of efficacy, biomarker, medical resource utilization, and safety measurements applicable to this study. The SoA (Table 3) summarizes the frequency and timing of PK assessments.

If multiple assessments are scheduled for the same timepoint, it is recommended that procedures be performed in the following sequence: PRO questionnaires first (to prevent influencing participant perceptions), electrocardiograms (ECGs), vital signs, and any type of blood draw last. Blood collections for PK assessments should be kept as close to the specified time as possible. Other measurements may be done earlier than specified timepoints if needed. Actual dates and times of assessments will be recorded in the source documentation and eCRF. Other measurements may be done earlier than specified timepoints if needed.

Medical resource utilization data will be collected. Refer to Section 8.7, Medical Resource Utilization and Health Economics for details.

Qualitative Exit Interviews will be conducted with a sub-set of participants. Refer to Section 8.8, Qualitative Exit Interview, for details.

For each participant, the maximum amount of blood drawn should not exceed 60 mL at any visit. Refer to the laboratory manual for details regarding blood volumes to be collected for each visit. Repeat or unscheduled samples may be taken for safety reasons or for technical issues with the samples.

The total blood volume to be collected from each participant will be approximately 1500 mL, depending upon what samples are taken and assuming 41 cycles of treatment and an EoT visit (see Section 4.2.2).

Sample Collection and Handling

The actual dates and times of sample collection must be recorded in the eCRF or laboratory requisition form.

Refer to the SoA for the timing and frequency of all sample collections.

Instructions for the collection, handling, storage, and shipment of samples are found in the laboratory manual that will be provided. Collection, handling, storage, and shipment of samples must be under the specified, and where applicable, controlled temperature conditions as indicated in laboratory manual.

Study-Specific Materials

The investigator will be provided with the following supplies:

- Investigator's brochures

- Pharmacy manual
- Master ICF
- Study protocol
- Laboratory manual

Additional documents will be provided as per local and study requirements.

Prescreening Phase For Biomarker Evaluation

Molecular eligibility (deleterious germline or somatic HRR gene alteration) will be established for each potential participant before screening for other eligibility criteria (Figure 2).

Serious adverse events (SAEs) related to the study procedures will be collected from the time the prescreening informed consent is obtained.

Screening Phase

Participants will enter the screening phase if the deleterious germline or somatic HRR gene alteration results are positive. During screening, eligibility criteria will be reviewed, and a complete clinical evaluation will be performed as specified in the SoA. Standard of care procedures performed prior to informed consent will be allowed during screening and include physical exams, vital signs, chemistry, hematology, coagulation panel (PT/INR and aPTT), PSA, ECG, CT/MRI and bone scans.

Treatment Phase

The Treatment Phase will begin at Cycle 1 Day 1 and will continue until study medications are discontinued. Participants must start study medication within 72 hours or 3 calendar days after randomization. The last measurements taken on Day 1 of Cycle 1 before administration of the study medication or at screening (whichever value was last) will be defined as the baseline values. Visits for each cycle will have a ±3-day window through Cycle 24, and a ±14-day window for cycles 25 through EoT, unless otherwise specified. Study visits will be calculated from the Cycle 1 Day 1 date, irrespective of any treatment interruptions. Participants may have imaging performed within ±7 days of visits requiring images. Refer to the SoA for treatment visits and assessments during the Treatment Phase.

Clinical evaluations and laboratory studies may be repeated more frequently, if clinically indicated. Any on-study surgical procedures should be captured on the Surgical Procedures form in the eCRF. Study medication will continue until disease progression, unacceptable toxicity, death, or the sponsor terminates the study. Patients with radiographic progression can remain on therapy if still receiving clinical benefit.

End-of-Treatment Visit

An EoT visit must be scheduled within 30 days after the last dose of study medication or prior to administration of a new anti-prostate cancer therapy, whichever occurs first. Refer to the SoA for required assessments at the EoT visit. If a participant is unable to return to the site for the EoT visit, then the participant should be contacted to collect AEs or SAEs that occurred, and concomitant medications taken, up to 30 days after the last dose of study medications, unless the participant has died, is lost to follow-up, or has withdrawn consent. If the information on concomitant therapies and AEs is obtained via telephone contact, then written documentation of the communication must be available for review in the source documents. If the participant dies, then the date and cause of death will be collected and documented in the eCRF. Note that bone, CT, or MRI scans performed ≤6 weeks prior to the EoT visit may serve as EoT scans. If the participant has consented to be contacted for the Exit Interview sub-study they should be reminded that they will be contacted by a third-party vendor Clinical Outcomes Solutions (COS) to schedule their interview (see also Section 8.8).

Follow-up Phase

Once a participant has completed the Treatment Phase for a reason other than radiographic progression, CT/MRI and bone scans (^{99m}Tc) should be collected every 4 months (±4 weeks) until confirmed radiographic progression provided the participant does not withdraw consent, is lost to follow-up, or dies. If a participant has documented radiographic progression during the Treatment Phase, additional radiographic assessments are not required during the Follow-up Phase.

After discontinuation of all study medications, participant status will be monitored every 4 months until participant death, lost to follow-up, withdrawal of consent, or study termination. Information regarding survival status and initiation of subsequent prostate cancer therapies, as well as disease progression status on the subsequent therapies, will be collected.

For the secondary and other endpoints, subsequent therapy, radiation, cancer related procedures, symptomatic progression, and morbid events will continue to be collected as specified in the SoA. Visits to the clinic are not required; sites may collect the information by telephone interview, chart review, or other convenient methods. If the follow-up information is obtained via telephone contact, then written documentation of the communication must be available for review in the source documents.

During the Follow-up Phase, deaths regardless of causality and SAEs thought to be related to study medications, including associated concomitant medications, will be collected and reported within 24 hours of discovery or notification of the event. Related AEs should be reported as per the procedures in Section 10.4, Appendix 4, Adverse Events: Definitions and Procedures for Recording, Evaluating, Follow-Up, and Reporting.

Information regarding study conduct during the Coronavirus Disease 2019 pandemic is available in Section 10.5, Appendix 5, Guidance on Study Conduct During the COVID-19 Pandemic.

8.1. Efficacy Assessments

Efficacy evaluations will be conducted as specified in the SoA. Unscheduled assessments should be considered if clinically indicated, and results collected in the eCRF.

8.1.1. Evaluations for Primary Efficacy Assessment

The primary endpoint for this study is rPFS as assessed by the investigator, which will be evaluated using chest, abdomen, and pelvis CT or MRI scans and whole-body bone scans (^{99m}Tc). Positron emission tomography or positron emission tomography CT scans cannot be used in place of technetium bone scan, conventional CT, or MRI. The same imaging modality should be used throughout the evaluation of an individual participant, if possible. Unscheduled tumor assessment and appropriate imaging should be considered if signs or symptoms suggestive of disease progression, including escalating pain not attributed to another cause, worsening ECOG PS status grade, or physical examination findings consistent with disease progression, are recorded.

Evaluation of rPFS will also be assessed by blinded independent central review (see also Section 8.1.1.1 for definitions). All participant scans (CT/MRI and ^{99m}Tc bone scans) will be submitted to a third-party core imaging laboratory. It is important to the integrity of the study that all imaging studies are promptly forwarded to the core imaging laboratory throughout the study. Blinded Independent Central Review of imaging scans may be ceased upon Sponsor decision communicated to investigators by letter. Further details regarding materials to be forwarded for central quality assessment can be found in the Imaging Manual or Investigator Site File.

Sites are encouraged to continue submitting scans until radiographic progression is documented by both the investigator and blinded independent central review.

Participants without radiographic progression or death will be censored at the last disease assessment.

8.1.1.1. Criteria for Primary Endpoint

Radiographic progression should be evaluated as follows:

- Progression of soft tissue lesions measured by CT or MRI as defined in RECIST 1.1.
- Progression by bone lesions observed by bone scan based on PCWG3. Under these criteria, any bone progression must be confirmed by a subsequent scan ≥6 weeks later. The Week 8 scan (first post-treatment scan, ie, Cycle 3 Day 1) should be used as the reference to which all subsequent scans are compared to determine progression. Bone progression is defined as one of the following:
    1. Participant whose Week 8 scan is observed to have ≥2 new bone lesions would fall into one of the 2 categories below:
        a) Participant whose confirmatory scan (which is performed ≥6 weeks later) shows ≥2 new lesions compared with the Week 8 scan (ie, a total of ≥4 new lesions compared to baseline scan) will be considered to have bone scan progression at Week 8.

b) Participant whose confirmatory scan did not show ≥2 new lesions compared with the Week 8 scan will not be considered to have bone scan progression. The Week 8 scan will be considered as the reference scan to which subsequent scans are compared. The FIRST scan timepoint that shows ≥2 new lesions compared with the Week 8 scan will be considered as the bone scan progression timepoint if these new lesions are confirmed by a subsequent scan ≥6 weeks later.

2. For a participant whose Week 8 scan does not have ≥2 new bone lesions compared with the baseline scan, the FIRST scan timepoint that shows ≥2 new lesions compared with the Week 8 scan will be considered as the bone scan progression timepoint if these new lesions are confirmed by a subsequent scan ≥6 weeks later.

8.1.2. Evaluations for Other Efficacy Assessments

Other efficacy evaluations include the following:

- Survival status
- Symptomatic progression
- Subsequent systemic therapy for prostate cancer
- Cancer-related radiation therapy or surgical procedures
- Serum PSA
- PROs
- ECOG PS

8.1.2.1. Patient Reported Outcomes

Disease-related symptoms will be assessed using the Brief Pain Inventory-Short Form (BPI-SF) and the prostate cancer symptom subscale of the Functional Assessment of Cancer Therapy-Prostate (FACT-P). The single item GP5 from the FACT-P regarding “bothered by side effects” will be described to evaluate treatment-related tolerability from the patient perspective. Overall health-related quality of life (HRQoL) will be measured with the FACT-P and the EQ-5D-5L to assess any impact to HRQoL from either disease-related or treatment-related experiences. Patient-reported outcomes should be collected as per the timepoints in SoA.

All visit-specific PRO assessments should be conducted and completed before any tests, procedures, or other consultations for that visit to prevent influencing participant perceptions. Detailed instructions for administering the PRO questionnaires will be provided in a PRO manual.

All PRO questionnaires can be completed either at the site, via the web-based platform or over the telephone as per the time points in the schedule of activities (SOA). In rare circumstances, such as in case of technical issues that cannot be resolved during the visit and as a last resort where other options for electronic completion have been explored and deemed not feasible, a paper questionnaire should be completed by the participant, and submitted to the vendor by site staff. Every effort should be made to have the ePRO completed by patient electronically.

During the Follow-Up Phase, only the EQ-5D-5L will be required to be completed for 1-year post-study medication discontinuation and this can be either at the site, via the web-based platform or over the telephone.

Efforts will be made to minimize and manage missing data to show a robust result (see Section 9.4.4.1). Sites will be proactively trained on how to assist participants with PRO collection and sufficient time will be given to complete PROs. Electronic PRO information will also be monitored to evaluate for any trends in missing data so that investigative site staff can be retrained, if needed.

8.1.2.1.1. BPI-SF

The BPI-SF is a self-administered, 11-item questionnaire, including 4 items regarding pain intensity and 7 items on how pain has interfered with the participant's life and activities.[8,10] The recall period for the majority of the items is the last 24 hours. All items are rated on an 11-point numeric rating scale (0-10), with pain intensity items scored from 0 (no pain) to 10 (pain as bad as you can imagine) and pain interference items scored from 0 (does not interfere) to 10 (completely interferes). The BPI-SF can generally be completed in 5 minutes.

8.1.2.1.2. FACT-P

The FACT-P is a self-administered, 39-item questionnaire measuring HRQoL in participants with prostate cancer.[16] The recall period for all items is the past 7 days. The FACT-P includes 4 subscales that make up the FACT-General (FACT-G version 4): physical well-being, social/family well-being, emotional well-being, and functional well-being plus a prostate cancer-specific subscale. Responses to all items are rated on a 5-point Likert response scale ranging from 0 "not at all" to 4 "very much". The FACT-P can generally be completed in 8-10 minutes. Total score is calculated with general function and prostate-cancer-specific scores and ranges from 0 to 156 (higher scores indicate better functional status).

8.1.2.1.3. EQ-5D-5L

The EQ-5D-5L is a self-administered, standardized measure of health status in a wide range of health conditions and treatments.[17,23,27] It provides a descriptive profile and a single index value for health status that can be used in the clinical and economic evaluation of health care. The recall period for all items is 'Today'. The EQ-5D-5L consists of the EQ-5D descriptive system and the EQ visual analogue scale (EQ-VAS). The EQ-5D descriptive system is comprised of 5 items across the following 5 dimensions: mobility, self-care, usual activities, pain/discomfort and anxiety/depression. The EQ-5D-5L uses a 5-point Likert response scale ranging from "No problems" to "Extreme problems". The EQ-5D health states defined by the EQ-5D descriptive system can be converted into a single index value using country-specific value sets. The index value facilitates the calculation of quality-adjusted life years that are used to inform economic evaluations of health care interventions. The EQ-5D also includes an EQ VAS that has endpoints labeled "best imaginable health state" and "worst imaginable health state" anchored at 100 and 0, respectively. Participants are asked to indicate how they rate their own health by indicating the point on the EQ VAS which best represents their own health on that day. The EQ5D-5L can generally be completed in 2-3 minutes.

8.1.2.1.4. PRO CTCAE

The NCI Patient Reported Outcomes version of the Common Terminology Criteria for Adverse Events (PRO-CTCAE™) Measurement System was developed to evaluate symptomatic toxicities by self-report in adults, adolescents and children participating in cancer clinical trials. It was designed to be used as a companion to the Common Terminology Criteria for Adverse Events (CTCAE), the standard lexicon for adverse event reporting in cancer trials. The PRO-CTCAE assessment is being piloted to gain experience with the use of this assessment for this patient population. Therefore, it will only be performed in the US and in English.

8.1.2.2. ECOG Performance Status

The ECOG PS scale (provided in Section 10.6, Appendix 6, ECOG Performance Status) will be used to grade changes in the participant's daily living activities. The frequency of ECOG PS assessment is provided in the SoA (Table 2).

8.2. Safety Assessments

Safety assessments will be based on medical review of AE reports and the results of physical examinations, vital sign measurements (heart rate and blood pressure), ECGs, and clinical laboratory tests as described in the SoA (Table 2).

Details regarding the IDMC are provided in Committees Structure in Section 10.2, Appendix 2, Regulatory, Ethical, and Study Oversight Considerations.

Adverse events will be reported and followed by the investigator as specified in Section 8.3, Adverse Events and Serious Adverse Events and Section 10.4, Appendix 4, Adverse Events: Definitions and Procedures for Recording, Evaluating, Follow-Up, and Reporting.

Any clinically significant abnormalities persisting at the end of the study/early withdrawal will be followed by the investigator until resolution or until a clinically stable condition is reached.

The study will include the following evaluations of safety and tolerability according to the time points provided in the SoA.

8.2.1. Physical Examinations

The screening physical examination will include, at a minimum, the general appearance of the participant, height and weight, examination of the skin, ears, nose, throat, lungs, heart, abdomen, extremities, musculoskeletal system, lymphatic system, and nervous system. During the Treatment Phase and at the EoT visit, limited symptom-directed physical examination is required. Only clinically relevant abnormalities found on physical examination should be reported as AEs in the eCRF.

8.2.2. Vital Signs

As hypertension has been reported with both AA and niraparib blood pressure and heart rate will be monitored weekly during the first 2 months (Cycles 1-2), every 2 weeks in Cycle 3, once per

month in Cycles 4 through 24, and then every 4 cycles thereafter through the End of Treatment. Blood pressure collection methodology may occur as deemed reliable per investigator decision.

Pulse/heart rate and blood pressure will be assessed and recorded on the eCRF.

Pulse/heart rate and blood pressure measurements will be assessed with a completely automated device. Manual techniques will be used only if an automated device is not available.

Blood pressure and heart rate monitoring can occur at clinic visit or may also be reported to the site by a home nurse or other qualified medical professionals approved by the investigator and authorized to examine patients.

8.2.3. ECGs

ECGs (12-lead) will be recorded at screening and as clinically indicated. Computer-generated interpretations of ECGs should be reviewed for data integrity and reasonableness by the investigator.

During the collection of ECGs, participants should be in a quiet setting without distractions (eg, television, cell phones). Participants should rest in a supine position for at least 5 minutes before ECG collection and should refrain from talking or moving arms or legs. If blood sampling or vital sign measurement is scheduled for the same time point as ECG recording, the procedures should be performed in the following order: ECG(s), vital signs, blood draw.

8.2.4. Clinical Safety Laboratory Assessments

Blood samples for serum chemistry and hematology will be collected as noted in Section 10.7, Appendix 7, Clinical Laboratory Tests. The investigator must review the laboratory results, document this review, and record any clinically relevant changes occurring during the study in the adverse event section of the eCRF.

Scheduled blood samples to assess the safety of the study medications will typically be processed by a central laboratory. Local laboratory tests may be performed as necessary. The investigator must review laboratory results, document this review, and record any clinically relevant changes occurring during the study in the AE section of the eCRF. For each clinically relevant laboratory abnormality reported as an AE, the following should be reported: the value indicative of the onset of each toxicity grade; the most abnormal value observed during the AE, and, if applicable, the value supporting recovery to Grade 1 or to baseline values.

In the event of additional safety monitoring, unscheduled laboratory assessments may be performed as required.

For any suspected MDS/AML case reported while a participant is receiving treatment or being followed for post-treatment assessments, bone marrow aspirate and biopsy testing must be completed. The study site must receive a copy of the pathology evaluation of the aspirate/biopsy findings, and other sample testing reports related to MDS/AML. Data from the report will be

entered on the appropriate eCRF pages and the site must keep a copy of the report with the participant's study file.

Clinically laboratory tests will be performed as outlined in the SoA (Table 2) and Section 10.7, Appendix 7, Clinical Laboratory Tests. Additional testing may be performed as clinically indicated.

8.3. Adverse Events and Serious Adverse Events

Timely, accurate, and complete reporting and analysis of safety information, including adverse events, serious adverse events, and product quality complaints (PQC) from clinical studies are crucial for the protection of participants, investigators, and the sponsor, and are mandated by regulatory agencies worldwide. The sponsor has established Standard Operating Procedures in conformity with regulatory requirements worldwide to ensure appropriate reporting of safety information; all clinical studies conducted by the sponsor or its affiliates will be conducted in accordance with those procedures.

Adverse events will be reported by the participant (or, when appropriate, by a caregiver, surrogate, or the participant's legally acceptable representative) for the duration of the study.

For further details on AEs and SAEs (Definitions and Classifications; Attribution Definitions; Severity Criteria; Special Reporting Situations; Procedures) as well as product quality complaints, refer to Section 10.4, Appendix 4, Adverse Events: Definitions and Procedures for Recording, Evaluating, Follow-Up, and Reporting.

8.3.1. Time Period and Frequency for Collecting Adverse Event and Serious Adverse Event Information

All Adverse Events

All AEs and special reporting situations, whether serious or non-serious, will be reported from the time informed consent is obtained until completion of the participant's last study-related procedure, which may include contact for follow-up of safety. Serious adverse events, including those spontaneously reported to the investigator within 30 days after the last dose of study medication, must be reported using the Serious Adverse Event Form. The sponsor will evaluate any safety information that is spontaneously reported by an investigator beyond the time frame specified in the protocol.

Serious Adverse Events

All SAEs, as well as PQC, occurring during the study must be reported to the appropriate sponsor contact person by study-site personnel within 24 hours of their knowledge of the event. Information regarding SAEs must be completed and transmitted to the sponsor using the Serious Adverse Event Form and Safety Report form of the eCRF, as well as reviewed by a physician from the study site, within 24 hours of awareness of the SAE. The initial and follow-up reports of a SAE should be transmitted electronically or by facsimile (fax). Telephone reporting should be the exception and the reporter should be asked to complete the appropriate form(s) first.

8.3.2. Method of Detecting Adverse Events and Serious Adverse Events

Care will be taken not to introduce bias when detecting AEs or SAEs. Open-ended and nonleading verbal questioning of the participant is the preferred method to inquire about AE occurrence.

Solicited AEs

Solicited AEs are predefined local and systemic events for which the participant is specifically questioned.

Unsolicited AEs

Unsolicited AEs are all adverse events for which the participant is not specifically questioned.

8.3.3. Follow-up of Adverse Events and Serious Adverse Events

The investigator is obligated to perform or arrange for the conduct of supplemental measurements and evaluations as medically indicated to elucidate the nature and causality of the adverse event, serious adverse event, or PQC as fully as possible. This may include additional laboratory tests or investigations, histopathological examinations, or consultation with other health care professionals.

Adverse events, including pregnancy, will be followed by the investigator as specified in Section 10.4, Appendix 4, Adverse Events: Definitions and Procedures for Recording, Evaluating, Follow-up, and Reporting.

8.3.4. Regulatory Reporting Requirements for Serious Adverse Events and Anticipated Events

The sponsor assumes responsibility for appropriate reporting of the safety information to the regulatory authorities/Independent Ethics Committee (IEC)/Institutional Review Board (IRB) in each respective country/territory, as applicable. The sponsor will also report to the investigator (and the head of the investigational institute where required) all suspected unexpected serious adverse reactions (SUSARs). The investigator (or sponsor where required) must report SUSARs to the appropriate IEC/IRB that approved the protocol unless otherwise required and documented by the IEC/IRB. A SUSAR will be reported to regulatory authorities unblinded. Participating investigators and IEC/IRB will receive a blinded SUSAR summary, unless otherwise specified.

An anticipated event is an AE that commonly occurs in the study population independent of exposure to the drug under investigation.

These anticipated events will be periodically analyzed in aggregate by the sponsor during study conduct. The sponsor will prepare a safety report in narrative format if the aggregate analysis indicates that the anticipated event occurs more frequently in the treatment group than in the control group and the sponsor concludes there is a reasonable possibility that the drug under investigation caused the anticipated event.

The plan for monitoring and analyzing the anticipated events is specified in a separate Anticipated Events Safety Monitoring Plan. The assessment of causality will be made by the sponsor's unblinded safety assessment committee.

The sponsor assumes responsibility for appropriate reporting of the listed anticipated events according to the requirements of the countries/territories in which the studies are conducted.

Anticipated events are discussed further in Section 10.8, Appendix 8, Anticipated Adverse Events.

8.3.5. Pregnancy

Niraparib has the potential to cause teratogenicity or embryo-fetal death because niraparib is genotoxic and targets actively dividing cells in animals and patients (eg, bone marrow). In animal studies, niraparib showed effects on sperm (reduced sperm count, spermatids, and germ cells in epididymites and testes). Based on animal studies, niraparib may impair fertility in males of reproductive potential. Abiraterone acetate was not noted to be genotoxic in in vitro studies; however, abiraterone is contraindicated in women who are or may become pregnant.[26]

All initial reports of pregnancy in partners of male participants must be reported to the sponsor by the study-site personnel within 24 hours of their knowledge of the event using the appropriate pregnancy notification form. Abnormal pregnancy outcomes (eg, spontaneous abortion, fetal death, stillbirth, congenital anomalies, ectopic pregnancy) are considered SAEs and must be reported using the Serious Adverse Event Form.

Follow-up information regarding the outcome of the pregnancy in a partner of a male participant and any postnatal sequelae in the infant will be required. Pregnant partners of male participants should also be appraised of the potential risk to the fetus.

8.3.6. Disease-Related Events and Disease-Related Outcomes Not Qualifying as Adverse Events or Serious Adverse Events

Progression of disease should not be considered nor should be reported as an AE (or SAE). However, signs and symptoms of disease progression or of clinical sequelae resulting from disease progression/lack of efficacy that are determined by the investigator to be of clinical significance should be reported per the usual reporting requirements (refer to Adverse Event Definitions and Classifications in Section 10.4, Appendix 4, Adverse Events: Definitions and Procedures for Recording, Evaluating, Follow-up, and Reporting). All participants must be followed for survival until death and information relating to a participant's death (eg, date of death and primary cause of death) should be recorded. Fatal AEs (regardless of relationship to study medication) after screening should be reported as SAEs for participants until 30 days after the last dose of study medications. Death is an outcome of an AE and not an AE itself. All reports of death due to an AE within 30 days of the last dose of study medication should include an AE term for the cause of death (if known). Fatal events occurring after that 30-day window will not be reported as SAEs and will be captured on the designated case report form.

8.3.7. Adverse Events of Special Interest

Adverse events of clinical interest are:

- MDS/AML
- Grade 3 or higher anemia, thrombocytopenia, and/or neutropenia.
- Hypertension
- Hypokalemia
- Fluid retention/edema
- Hepatotoxicity
- Osteoporosis (including fracture)
- Rhabdomyolysis/myopathy
- Allergic alveolitis
- CYP2D drug interactions and food effect
- Major Adverse Cardiovascular Events (MACE).

Participants with these AEs of special interest may be counted or listed.

8.4. Pharmacokinetics

Blood samples will be used to evaluate the PK of niraparib and its metabolite, M1. Blood collected for PK may additionally be used to evaluate safety or efficacy and exploratory exposure response analysis aspects that address concerns arising during or after the study period. Genetic analyses will not be performed on these serum samples. Participant confidentiality will be maintained.

8.4.1. Evaluations

Venous blood samples of approximately 2 mL will be collected from all participants for measurement of plasma concentrations of niraparib and its metabolite, M1, on Day 1 of Cycles 2 through 7, as specified in the SoA Table 3.

Samples collected for analyses of the plasma concentration for niraparib and its metabolite, M1, may additionally be used to evaluate safety or efficacy aspects that address concerns arising during or after the study period or for the evaluation of relevant biomarkers. Participant confidentiality will be maintained. Additional information about the collection, handling, and shipment of biological samples can be found in the Laboratory Manual.

8.4.2. Analytical Procedures

Pharmacokinetics

Plasma samples will be analyzed to determine concentrations of niraparib and its metabolite, M1, using a validated liquid chromatography with tandem mass spectrometry (LC-MS/MS) method by or under the supervision of the sponsor. If required, some plasma samples may be analyzed to document the presence of circulating metabolites using a qualified research method.

8.4.3. Pharmacokinetic Parameters and Evaluations

Parameters

Based on the individual plasma concentration-time data, using the actual dose taken and the actual sampling times, PK parameters and exposure information of niraparib and its metabolite, M1, will be derived using population PK modelling. Baseline covariates (eg, body weight, age, sex, creatinine clearance, race) may be included in the model, if relevant.

Pharmacokinetic/Pharmacodynamic Evaluations

Niraparib exposure-response relationship will be explored for key efficacy (eg, rPFS, OS) and safety parameters as data allow.

8.5. Genetics

A blood sample will be collected to allow for pharmacogenomic evaluations of deleterious germline HRR gene alterations during prescreening (where local regulations permit). An on-study blood sample will be collected (where local regulations permit) and may be used for additional research to identify predictors of response to niraparib and AA or to allow for bridging and/or diagnostic test development as local regulations permit.

8.6. Biomarkers

Sample collection and testing will comply with local regulations.

As described in Section 4.2, Scientific Rationale for Study Design, Biomarker Collection and Section 5, Study Population, participants must have demonstrated deleterious germline or somatic HRR gene alterations.

Stopping Analysis

Biomarker analyses are dependent upon the availability of appropriate biomarker assays and clinical response rates. Biomarker analysis may be deferred or not performed, if during or at the end of the study, it becomes clear that the analysis will not have sufficient scientific value for biomarker evaluation, or if there are not enough samples or responders to allow for adequate biomarker evaluation. In the event the study is terminated early or shows poor clinical efficacy, completion of biomarker assessments is based on justification and intended utility of the data.

Additional Collections

If it is determined at any time before study completion that additional material is needed from a formalin-fixed, paraffin-embedded tumor sample for the successful completion of the protocol-specified analyses, the sponsor may request that additional material be retrieved from existing samples. Also, based on emerging scientific evidence, the sponsor may request additional material from previously collected tumor samples during or after study completion for a retrospective analysis. In this case, such analyses would be specific to research related to the study medication(s) or diseases being investigated. These additional sample collections and analyses will only be performed as local regulations permit.

8.6.1. Evaluations

Exploratory biomarkers (including DNA, RNA, or protein) will be collected where local regulations allow. Any samples remaining after the protocol defined research analyses described below may be used for future studies.

8.6.1.1. Plasma for RNA

Plasma samples will be collected as specified in the SoA. Multiple RNA transcripts found in prostate tumors are detectable in the plasma and these samples may be used to evaluate potential mechanisms of resistance or response that may emerge with niraparib.

8.6.1.2. Plasma for DNA

Plasma samples will be collected as specified in SoA and will be used to isolate circulating tumor DNA (ctDNA) to evaluate deleterious germline or somatic HRR alterations status (where local regulations permit) and may also be used to identify additional DNA biomarkers. In addition, the cell pellet will be collected from Cycle 1 Day 1 to serve as a reference genome for sequencing analysis. Plasma isolated ctDNA may be evaluated for changes in alterations observed over time, to monitor for potential markers of resistance or response to niraparib or AA plus prednisone, minimal residual disease or to further develop the diagnostic test to identify patients suitable for treatment with therapeutic regimens that include niraparib. Plasma samples may also be used to develop tests/assays related to niraparib and AA and prostate cancer.

8.6.1.3. Tumor Tissue Analysis

Tumor tissue (either archival or recently collected) will be obtained to evaluate deleterious germline or somatic HRR alteration status (when indicated) and may also be used to identify additional DNA biomarkers associated with response to niraparib or AA plus prednisone. Furthermore, tumor tissue may be assessed for RNA or protein signatures predictive of response. Tumor tissue may also be used for potential bridging studies or to further develop the diagnostic test to identify patients suitable for treatment with therapeutic regimens that include niraparib.

8.7. Medical Resource Utilization and Health Economics

Health Economics parameters are not evaluated in this study.

Medical resource utilization data, associated with medical encounters, will be collected in the eCRF by the investigator and study-site personnel for all participants throughout the study. Protocol-mandated procedures, tests, and encounters are excluded. The data collected may be used to conduct exploratory economic analyses and will include:

- Number and duration of medical care encounters, including surgeries, and other selected procedures (inpatient and outpatient)
- Duration of hospitalization (total days length of stay, including duration by wards, eg, intensive care unit)
- Number and character of diagnostic and therapeutic tests and procedures

- Outpatient medical encounters and treatments (including physician or emergency room visits, tests and procedures, and medications).

8.8. Qualitative Exit Interview

Qualitative Exit Interviews will be conducted as a sub-study in consenting participants at selected study sites. Participants will be offered a consent for the Exit Interview and may choose to forgo Exit Interviews without impacting any other aspect of the study.

Exit Interviews will be conducted by a third-party vendor, Clinical Outcomes Solutions (COS), using interviewers trained in qualitative semi-structured interviewing.

Consenting participants will be entered into a Participant Tracker by study-site personnel via secure HIPAA and General Data Protection Regulation (GDPR) compliant file sharing. COS will track participants' expected EOT visit timing in order to contact the participant to schedule the interview as close as possible after their scheduled EOT visit.

The interviews will be conducted via online video call or telephone and last approximately 60 minutes. Interviews follow a semi-structured interview guide using open-ended questions and probes to guide discussion without leading. Interviews will explore participants' experience of treatment benefit, including questions about changes in symptoms or function and the meaning or impact of perceived changes.

During the interview participants may discuss side effects that could be AEs and may or may not have already been reported by the study site investigator. All interviewers will be trained on AE reporting requirements and will inform the study site via secure file sharing if the participant reports any potential AEs during the Exit Interview. In order to avoid any duplication of AE reports the study site investigator will be responsible for handling the report as needed as per section 8.3. There will be no further reconciliation of AEs with the Exit Interviews and interviewers will not follow-up with participants after their interview.

- Further details on the Exit Interview process can be found in the "Study Guide for Qualitative Interview Sub-Study to the Amplitude Clinical Trial in Prostate Cancer" and associated Participant Interview Guide.

9. STATISTICAL CONSIDERATIONS

Statistical analysis will be done by the sponsor or under the authority of the sponsor. A general description of the statistical methods to be used to analyze the efficacy and safety data is outlined below. Specific details will be provided in the Statistical Analysis Plan (SAP).

9.1. Statistical Hypotheses

The hypothesis is that niraparib and AA, plus prednisone will demonstrate improved rPFS compared with AA plus prednisone in all participants with deleterious germline or somatic HRR gene-mutated mCSPC.

9.2. Sample Size Determination

Approximately 692 participants will be randomized in a 1:1 ratio to receive niraparib and AA, plus prednisone or AA plus prednisone. It is estimated that approximately [redacted] will be required to provide 95% power in detecting a HR of 0.64 [redacted] plus prednisone treatment arm versus [redacted] plus prednisone treatment arm) at a 2-tailed level of significance of 0.05. With a [redacted]-month accrual period and an additional [redacted] months of follow-up, the study duration to reach the required number of rPFS events will be approximately [redacted] months.

Long term survival follow-up will continue until approximately [redacted] events have been observed. [redacted]

The sponsor may delineate a target subpopulation of genes to be specified based on emerging external data and may adjust accrual accordingly.

9.3. Populations for Analyses

For purposes of analysis, the following populations are defined:

Population	Description
Intent-to-treat (ITT)	All randomized participants classified according to their assigned treatment group, regardless of the actual treatment received. Participant disposition and efficacy analyses will be performed on data from the ITT population
Safety	All randomized participants who take at least 1 dose of study medication as treated.

9.4. Statistical Analyses

The SAP will be finalized prior to database lock and it will include a more technical and detailed description of the statistical analyses described in this section. This section is a summary of the planned statistical analyses of the most important endpoints including primary and key secondary endpoints.

All continuous variables will be summarized using number of participant (n), mean, standard deviation (SD), median, minimum, and maximum. Discrete variables will be summarized with number and percent. All efficacy endpoints will be analyzed using the ITT population. The Kaplan Meier product limit method and a stratified Cox model will be used to estimate the time-to-event variables and to obtain the HR along with the associated confidence intervals. Unless otherwise specified, stratified log-rank tests will be used to test the treatment effect for time-to-event variables; response rate variables will be evaluated using the chi square statistic or the exact test if the cell counts are small.

9.4.1. General Considerations

9.4.1.1. Multiplicity Adjustment for Testing of Primary and Key Secondary Endpoints

The SAP will provide details on the graphical testing framework that will be used to strictly control the familywise type 1 error rate at 2-sided 0.05 level.[33]

9.4.2. Primary Endpoint

The primary endpoint is rPFS, as assessed by the investigator, and defined as the time from the date of randomization to the date of radiographic progression or death, whichever occurs first. Radiographic progression is defined in Section 8.1.1.

See the SAP for further details about the analysis of the primary endpoint, including sensitivity and subgroup analyses.

9.4.3. Secondary Endpoints

The secondary endpoints are defined as below (details in the SAP):

- OS: defined as the time from date of randomization to date of death due to any cause. Participants alive at the time of analysis will be censored on the last date the participant was known to be alive.
- Time to symptomatic progression: defined as time from date of randomization to the date of any of the following (whichever occurs first):

    The use of external beam radiation for skeletal or pelvic symptoms. *Note:* Only radiation planned prior to randomization will not be considered as symptomatic progression

    The need for tumor-related orthopedic surgical intervention

    Other cancer-related procedures (eg, nephrostomy insertion, bladder catheter insertion, or surgery for tumor symptoms)

    Cancer-related morbid events (ie, fracture [symptomatic and/or pathologic], cord compression, urinary obstructive events)

    Initiation of a new systemic anti-cancer therapy because of cancer symptoms

- Time to subsequent therapy: defined as the time from date of randomization to the date of initiation subsequent therapy for prostate cancer.

9.4.4. Other Endpoints

Other endpoints include the following:

- Patient-reported outcomes (see Section 9.4.4.1)
- PFS2 Time from date of randomization to date of first occurrence of disease progression (radiographic, clinical, or PSA progression) on first subsequent therapy for prostate cancer or death, whichever occurs first

- Objective response is defined as achieving a partial response or complete response according to modified RECIST 1.1.
- Time to PSA progression is defined as the time from the date of randomization to the date of PSA progression based on PCWG3 criteria.[49]
- Objective response, rPFS, and PSA response across individual and subgroups of deleterious germline or somatic HRR gene alterations (see Section 9.4.6).

9.4.4.1. Patient-reported Outcomes Analyses

Established minimum clinically important differences will be used for time-to-deterioration and proportional analyses.[6,7] The minimum clinically important differences for this study, based on the PROs selected, are provided in Table 10.

Table 10: Minimum Clinically Important Differences

PRO total score or domain	Threshold for definition of deterioration
	Scores increasing reflect a worsening/deterioration
Worst Pain (BPI Question #3)	≥30% of baseline<br>≥2 points
Pain Interference score (combination of BPI scale scores 9a through 9g)	≥ half standard deviation of baseline
Average pain (Average of BPI3-6)	≥30% of baseline
FACT-P	Scores decreasing is worsening/deterioration
Physical Well-Being (PWB)	≤3
Social/Family Well-Being (SFWB)	≤3
Emotional Well-Being (EWB)	≤3
Functional Well-Being (FWB)	≤3
FACT-G (General) Scale	≤9
Prostate Cancer Subscale (PCS)	≤3
Trial Outcome Index (TOI)	≤9
FACT-P Total Scale	≤10
FACT-P Pain Scale	≤2
FAPSI-8	≤3
EQ5D HUI	0.07 and 0.09
EQ VAS[41]	7 and 10

PROs will be analyzed by accounting for change from baseline with a mixed model of repeated measures and using a pattern mixture model as a sensitivity analysis. Fixed effects for the models will include treatment and visit number as discrete parameters, and interaction between time and treatment; participant is included as random effect. In addition, as a sensitivity analysis, a pattern mixed model will be applied assuming PRO data is not missing at random.[31]

Time-to-deterioration and mixed model of repeated measures will be reported for BPI item 3, BPI Pain Interference, BPI Average pain, FACT-P total and all subscale scores, and EQ-5D-5L Health Utility Index and VAS scores. Individual items of particular interest (eg, FACT-P item GP5 “I am bothered by side effects of treatment”) will be described by distribution of item responses. Details of PRO analyses will be provided in the PRO-SAP.

9.4.5. Safety Analyses

All safety analyses will be made on the Safety Population.

Adverse Events

The verbatim terms used in the eCRF by investigators to identify adverse events will be coded using the Medical Dictionary for Regulatory Activities. Any AE occurring at or after the initial administration of study medication through the day of last dose plus 30 days is considered to be treatment-emergent. All reported treatment-emergent AEs will be included in the analysis. For each AE, the percentage of participants who experience at least 1 occurrence of the given event will be summarized by treatment group.

Summaries, listings, datasets, or participant narratives may be provided, as appropriate, for those participants who die, who discontinue study medication due to an AE, or who experience a severe or a serious adverse event.

Parameters with predefined NCI-CTCAE toxicity grades will be summarized. Change from baseline to the worst adverse event grade experienced by the participant during the study will be provided as shift tables.

Clinical Laboratory Tests

Laboratory data will be summarized by type of laboratory test. Descriptive statistics will be calculated for each laboratory analyte at baseline and for observed values and changes from baseline at each scheduled time point. Parameters with predefined toxicity grades will be summarized. Change from baseline to the worst grade experienced by the participant during the study will be provided as shift tables.

Vital Signs

Vital signs including pulse/heart rate and blood pressure (systolic and diastolic) values and changes from baseline will be summarized over time, using descriptive statistics and/or graphically. The percentage of participants with values beyond clinically important limits from baseline will be summarized.

Physical Examinations

Abnormal findings in physical examination will be recorded and summarized as AEs.

9.4.6. Other Analyses

Pharmacokinetic Analyses

If feasible, population PK analysis of plasma concentration-time data of niraparib (and its M1 metabolite if needed) may be performed using nonlinear mixed-effects modeling. Data may be combined with those of other selected studies to support a relevant structural model. Available baseline participant characteristics (demographics, laboratory variables, genotypes, race, etc.) will be tested as potential covariates affecting PK parameters. Details will be given in a population PK analysis plan and the results of the population PK analysis will be presented in a separate report.

A snapshot date for PK samples to be analyzed will be defined, if required. Samples collected before this date will be analyzed for niraparib and M1 and included in the population PK analysis.

Samples collected after the snapshot date will be analyzed at a later date and may be included in a population PK re-analysis when they become available after database lock.

Data will be listed for all participants with available plasma concentrations of niraparib and M1 per treatment group. Participants will be excluded from the PK analysis if their data do not allow for accurate assessment of the PK (eg, incomplete administration of the study medication; missing information of dosing and sampling times; concentration data not sufficient for PK parameter calculation).

All concentrations below the lowest quantifiable concentration or missing data will be labeled as such in the concentration database. All participants and samples excluded from the analysis will be clearly documented in the population PK analysis report.

Descriptive statistics, including arithmetic mean, SD, coefficient of variation, median, minimum, and maximum will be calculated for all individual derived relevant PK parameters for niraparib.

Biomarker Analyses

The association of biomarker-positivity with clinical response or time-to-event endpoints will be assessed using appropriate statistical methods, (such as analysis of variance, categorical, or survival models), depending on the endpoints.

Adjustments regarding the inclusion of participants with specific deleterious germline or somatic HRR gene alterations may be made based on evidence from external data or to ensure enough participants are enrolled to allow for subgroup analysis. Any biomarker-related enrollment changes will be communicated to investigators.

Changes in RNA over time will be summarized by treatment group. Associations between baseline levels and changes from baseline in select markers and clinical response (ie, rPFS and/or PSA response) may be explored. Biomarker analyses will be summarized in separate technical reports.

DNA anomalies will be summarized by treatment group. Associations between gene level variants and groups of variants in selected or groups of genes and clinical response (ie. rPFS, and/or PSA response) may be explored.

Pharmacokinetic/Pharmacodynamic Analyses

The exposure-response relationship between niraparib measures of exposure (eg, derived AUC or trough concentrations) and key efficacy (eg, rPFS and OS) and safety parameters, will be explored graphically, as data allow. In addition, the relationship may be characterized using an exposure-response or logistic regression model. Details will be provided in an analysis plan and detailed results may be reported separately from the CSR.

Pharmacogenomic Analyses

DNA samples may be used for research related to niraparib and AA or prostate cancer. They may also be used to develop tests/assays related to niraparib and AA and prostate cancer.

Pharmacogenomic research may consist of the analysis of one or more candidate genes in relation to niraparib and AA or prostate cancer clinical endpoints.

Results may be presented in a separate report.

Medical Resource Utilization Analyses

Medical resource utilization will be descriptively summarized by treatment group. Detailed analyses of medical resource utilization will be reported separately from the CSR.

9.5. Interim Analysis for Overall Survival

Three formal interim analyses and a final analysis for OS are planned. Details are provided in the SAP.

9.6. Data Monitoring Committee or Other Review Board

An IDMC will be established as noted in Committees Structure in Section 10.2, Appendix 2, Regulatory, Ethical, and Study Oversight Considerations.

The IDMC will monitor data on an ongoing basis to ensure the continuing safety of the participants enrolled in the study and to review efficacy information. The IDMC responsibilities, authorities, and procedures will be documented in a separate charter.

Complete details regarding the composition and governance of the IDMC will be outlined in the IDMC Charter.

10. SUPPORTING DOCUMENTATION AND OPERATIONAL CONSIDERATIONS

10.1. Appendix 1: Abbreviations

^{99m}Tc	Technetium-99m
AA	abiraterone acetate
AAP	abiraterone acetate plus prednisone
ADT	androgen deprivation therapy
AE	adverse event
ALT	alanine aminotransferase
AML	acute myeloid leukemia
AR	androgen receptor
AST	aspartate aminotransferase
ATM	ataxia telangiectasia mutated
AUC	area under the concentration time curve
BP	blood pressure
BPI-SF	Brief Pain Inventory-Short Form
BRCA1	breast cancer 1
BRCA2	breast cancer 2
CDK12	cyclin dependent kinase 12
CHEK2	checkpoint kinase 2
COS	Clinical Outcome Solutions
COVID-19	Coronavirus Disease 2019
CT	computed tomography
CTCAE	Common Terminology Criteria for Adverse Events
ctDNA	circulating tumor deoxyribonucleic acid
CTR	Clinical Trial Regulation
CYP	cytochrome P450
DLT	dose-limiting toxicity
DNA	deoxyribonucleic acid
DRD	DNA-repair gene defect
eCRF	electronic case report form
ECG	electrocardiogram
ECOG PS	Eastern Cooperative Oncology Group Performance Status
eDC	electronic data capture
EI	Exit Interview
EoT	end-of-treatment
EQ-5D-5L	Euro-Quality of Life Questionnaire
EQ VAS	EQ-5D visual analogue scale
EU	European Union
FACT-P	Functional Assessment of Cancer Therapy-Prostate
FDC	fixed-dose combination
FOIA	Freedom of Information Act
GCP	Good Clinical Practice
GnRHa	gonadotropin-releasing hormone analogue
HR	hazard ratio
HRQoL	health-related quality of life
HRR	homologous recombination repair
IB	Investigator's Brochure
IC_{50}	half-maximal inhibitory concentration
ICF	informed consent form
ICH	International Council for Harmonisation
IDMC	Independent Data Monitoring Committee
IEC	Independent Ethics Committee
INR	International normalized ratio
IRB	Institutional Review Board
ITT	intent-to-treat
IUD	intrauterine device

IUS	intrauterine hormone releasing system
IWRS	interactive web response system
LFT	liver function test
LTE	Long-term Extension
mCRPC	metastatic castration-resistant prostate cancer
mCSPC	metastatic castration-sensitive prostate cancer
mHSPC	metastatic hormone-sensitive prostate cancer
MDS	myelodysplastic syndrome
MRI	magnetic resonance imaging
NCCN	National Comprehensive Cancer Network
NCI-CTCAE	National Cancer Institute Common Terminology Criteria for Adverse Events
OLE	Open-label Extension
OS	overall survival
PALB2	partner and localizer of BRCA2 gene
PARP(i)	poly (adenosine diphosphate-ribose) polymerase (inhibitor)
PCWG3	Prostate Cancer Working Group 3
PK	pharmacokinetic(s)
PQC	Product Quality Complaint
PRES	Posterior Reversible Encephalopathy Syndrome
PRO	patient-reported outcome(s) (paper or electronic as appropriate for this study)
PRO-CTCAE	Patient-reported Outcomes Common Terminology Criteria for Adverse Events
PSA	prostate sensitive antigen
RECIST	Response Evaluation Criteria in Solid Tumors
RNA	ribonucleic acid
rPFS	radiographic progression-free survival
SAE	serious adverse event
SAP	Statistical Analysis Plan
SD	standard deviation
SoA	Schedule of Activities
SUSAR	suspected unexpected serious adverse reaction
ULN	upper limit of normal
US	United States
VCaP	vertebral cancer of the prostate
WHO	World Health Organization

10.2. Appendix 2: Regulatory, Ethical, and Study Oversight Considerations

REGULATORY AND ETHICAL CONSIDERATIONS

Investigator Responsibilities

The investigator is responsible for ensuring that the study is performed in accordance with the protocol, current International Council for Harmonisation (ICH) guidelines on Good Clinical Practice (GCP), and applicable regulatory and country- or territory -specific requirements.

Good Clinical Practice is an international ethical and scientific quality standard for designing, conducting, recording, and reporting studies that involve the participation of human participants. Compliance with this standard provides public assurance that the rights, safety, and well-being of study participants are protected, consistent with the principles that originated in the Declaration of Helsinki, and that the study data are credible.

Protocol Amendments

Neither the investigator nor the sponsor will modify this protocol without a formal amendment by the sponsor. All protocol amendments must be issued by the sponsor and signed and dated by the investigator. Protocol amendments must not be implemented without prior IEC/IRB approval, or when the relevant competent authority has raised any grounds for non-acceptance, except when necessary to eliminate immediate hazards to the participants, in which case the amendment must be promptly submitted to the IEC/IRB and relevant competent authority. Documentation of amendment approval by the investigator and IEC/IRB must be provided to the sponsor. When the change(s) involve only logistic or administrative aspects of the study, the IEC/IRB (where required) only needs to be notified.

During the course of the study, in situations where a departure from the protocol is unavoidable, the investigator or other physician in attendance will contact the appropriate sponsor representative listed in the Contact Information page(s), which will be provided as a separate document. Except in emergency situations, this contact should be made before implementing any departure from the protocol. In all cases, contact with the sponsor must be made as soon as possible to discuss the situation and agree on an appropriate course of action. The data recorded in the eCRF and source documents will reflect any departure from the protocol, and the source documents will describe this departure and the circumstances requiring it.

Regulatory Approval/Notification

This protocol and any amendment(s) must be submitted to the appropriate regulatory authorities in each respective country/territory, if applicable. A study may not be initiated until all local regulatory requirements are met.

Required Prestudy Documentation

The following documents must be provided to the sponsor before shipment of study medication to the study site:

- Protocol and amendment(s), if any, signed and dated by the principal investigator
- A copy of the dated and signed (or sealed, where appropriate per local regulations), written IEC/IRB approval of the protocol, amendments, ICF, any recruiting materials, and if applicable, participant compensation programs. This approval must clearly identify the specific protocol by title and number and must be signed (or sealed, where appropriate per local regulations) by the chairman or authorized designee.
- Name and address of the IEC/IRB, including a current list of the IEC/IRB members and their function, with a statement that it is organized and operates according to GCP and the applicable laws and regulations. If accompanied by a letter of explanation, or equivalent, from the IEC/IRB, a general statement may be substituted for this list. If an investigator or a member of the study-site personnel is a member of the IEC/IRB, documentation must be obtained to state that this person did not participate in the deliberations or in the vote/opinion of the study.
- Regulatory authority approval or notification, if applicable
- Signed and dated statement of investigator (eg, Form FDA 1572), if applicable
- Documentation of investigator qualifications (eg, curriculum vitae)
- Completed investigator financial disclosure form from the principal investigator, where required
- Signed and dated clinical trial agreement, which includes the financial agreement
- Any other documentation required by local regulations

The following documents must be provided to the sponsor before enrollment of the first participant:

- Completed investigator financial disclosure forms from all subinvestigators
- Documentation of subinvestigator qualifications (eg, curriculum vitae)
- Name and address of any local laboratory conducting tests for the study, and a dated copy of current laboratory normal ranges for these tests, if applicable
- Local laboratory documentation demonstrating competence and test reliability (eg, accreditation/license), if applicable

Independent Ethics Committee or Institutional Review Board

Before the start of the study, the investigator (or sponsor where required) will provide the IEC/IRB with current and complete copies of the following documents (as required by local regulations):

- Final protocol and, if applicable, amendments
- Sponsor-approved ICF (and any other written materials to be provided to the participants)
- IB (or equivalent information) and amendments/addenda
- Sponsor-approved participant recruiting materials

- Information on compensation for study-related injuries or payment to participants for participation in the study, if applicable
- Investigator's curriculum vitae or equivalent information (unless not required, as documented by the IEC/IRB)
- Information regarding funding, name of the sponsor, institutional affiliations, other potential conflicts of interest, and incentives for participants
- Any other documents that the IEC/IRB requests to fulfill its obligation

This study will be undertaken only after the IEC/IRB has given full approval of the final protocol, amendments (if any, excluding the ones that are purely administrative, with no consequences for participants, data or study conduct, unless required locally), the ICF, applicable recruiting materials, and participant compensation programs, and the sponsor has received a copy of this approval. This approval letter must be dated and must clearly identify the IEC/IRB and the documents being approved.

Approval for the collection of samples for research and for the corresponding ICF must be obtained from the IEC/IRB.

During the study the investigator (or sponsor where required) will send the following documents and updates to the IEC/IRB for their review and approval, where appropriate:

- Protocol amendments (excluding the ones that are purely administrative, with no consequences for participants, data or study conduct)
- Revision(s) to ICF and any other written materials to be provided to participants
- If applicable, new or revised participant recruiting materials approved by the sponsor
- Revisions to compensation for study-related injuries or payment to participants for participation in the study, if applicable
- New edition(s) of the IB and amendments/addenda
- Summaries of the status of the study at intervals stipulated in guidelines of the IEC/IRB (at least annually)
- Reports of adverse events that are serious, unlisted/unexpected, and associated with the study medication
- New information that may adversely affect the safety of the participants or the conduct of the study
- Deviations from or changes to the protocol to eliminate immediate hazards to the participants
- Report of deaths of participants under the investigator's care
- Notification if a new investigator is responsible for the study at the site
- Development Safety Update Report and Line Listings, where applicable
- Any other requirements of the IEC/IRB

For all protocol amendments (excluding the ones that are purely administrative, with no consequences for participants, data or study conduct), the amendment and applicable ICF revisions must be submitted promptly to the IEC/IRB for review and approval before implementation of the change(s).

At least once a year, the IEC/IRB will be asked to review and reapprove this study, where required.

At the end of the study, the investigator (or sponsor where required) will notify the IEC/IRB about the study completion (if applicable, the notification will be submitted through the head of investigational institution).

Country/Territory Selection

This study will only be conducted in those countries/territories where the intent is to launch or otherwise help ensure access to the developed product if the need for the product persists, unless explicitly addressed as a specific ethical consideration in Section 4.2.1, Study-Specific Ethical Design Considerations.

Other Ethical Considerations

For study-specific ethical design considerations, refer to Section 4.2.1.

FINANCIAL DISCLOSURE

Investigators and subinvestigators will provide the sponsor with sufficient, accurate financial information in accordance with local regulations to allow the sponsor to submit complete and accurate financial certification or disclosure statements to the appropriate regulatory authorities. Investigators are responsible for providing information on financial interests during the course of the study and for 1 year after completion of the study.

INFORMED CONSENT PROCESS

Each participant must give written informed consent according to local requirements after the nature of the study has been fully explained. The ICF must be signed before performance of any study-related activity. The ICFs that are used must be approved by both the sponsor and by the reviewing IEC/IRB and be in a language that the participant can read and understand. Reconsent may be obtained remotely/virtually in accordance with sponsor procedures and where local regulations allow. Refer to local applicable guidelines to ensure compliance. The informed consent should be in accordance with principles that originated in the Declaration of Helsinki, current ICH and GCP guidelines, applicable regulatory requirements, and sponsor policy.

Before enrollment in the study, the investigator or an authorized member of the study-site personnel must explain to potential participants the aims, methods, reasonably anticipated benefits, and potential hazards of the study, and any discomfort participation in the study may entail. Participants will be informed that their participation is voluntary and that they may withdraw consent to participate at any time. They will be informed that choosing not to participate will not affect the care the participant will receive for the treatment of his disease. Participants will be told that alternative treatments are available if they refuse to take part and that such refusal will not

prejudice future treatment. Finally, they will be told that the investigator will maintain a participant identification register for the purposes of long-term follow-up if needed and that their records may be accessed by health authorities and authorized sponsor personnel without violating the confidentiality of the participant, to the extent permitted by the applicable law(s) or regulations. By signing the ICF, the participant is authorizing such access, which includes permission to obtain information about his or her survival status. It also denotes that the participant agrees to allow his or her study physician to recontact the participant for the purpose of obtaining consent for additional safety evaluations, and subsequent disease-related treatments, if needed. The physician may also recontact the participant for the purpose of obtaining consent to collect information about his or her survival status.

The participant will be given sufficient time to read the ICF and the opportunity to ask questions. After this explanation and before entry into the study, a written consent should be appropriately recorded by means of the participant's personally dated signature. After having obtained the consent, a copy of the ICF must be given to the participant.

Participants who are rescreened are required to provide new consent.

Participants will be asked for consent to provide samples for research (where local regulations permit). After informed consent for the study is appropriately obtained, the participant may be asked to provide separate informed consent to participate in the research component. A copy of the completed ICF will be given to the participant.

Where local regulations require, a separate ICF may be used for the required DNA component of the study.

RECRUITMENT STRATEGY

The first site open is considered the first act of recruitment and it becomes the study start date.

The Sponsor has provided the study sites with optional patient recruitment material templates, to be used in accordance with local regulations and policies.

DATA PROTECTION

Privacy of Personal Data

The collection and processing of personal data from participants enrolled in this study will be limited to those data that are necessary to fulfill the objectives of the study.

These data must be collected and processed with adequate precautions to ensure confidentiality and compliance with applicable data privacy protection laws and regulations. Appropriate technical and organizational measures to protect the personal data against unauthorized disclosures or access, accidental or unlawful destruction, or accidental loss or alteration must be put in place. Sponsor personnel whose responsibilities require access to personal data agree to keep the identity of participants confidential.

The informed consent obtained from the participant includes explicit consent for the processing of personal data and for the investigator/institution to allow direct access to his or her original medical records (source data/documents) for study-related monitoring, audit, IEC/IRB review, and regulatory inspection. This consent also addresses the transfer of the data to other entities and to other countries/territories.

The participant has the right to request through the investigator access to his or her personal data and the right to request rectification of any data that are not correct or complete. Reasonable steps will be taken to respond to such a request, taking into consideration the nature of the request, the conditions of the study, and the applicable laws and regulations.

In the event of a data security breach, the sponsor will apply measures to adequately manage and mitigate possible adverse effects taking into consideration the nature of the data security breach as necessary to address other obligations such as notifying appropriate authorities in accordance with applicable data protection law.

Exploratory DNA, pharmacogenomics, biomarker, and PK research is not conducted under standards appropriate for the return of data to participants. In addition, the sponsor cannot make decisions as to the significance of any findings resulting from exploratory research. Therefore, exploratory research data will not be returned to participants or investigators, unless required by law or local regulations. Privacy and confidentiality of data generated in the future on stored samples will be protected by the same standards applicable to all other clinical data.

LONG-TERM RETENTION OF SAMPLES FOR FUTURE RESEARCH

Samples collected in this study may be stored for up to 15 years (or according to local regulations) for future research. Samples will only be used to understand niraparib and AA, to understand prostate cancer in patients with deleterious germline or somatic HRR gene mutations, to understand differential study medication responders, and to develop tests/assays related to niraparib and AA and prostate cancer in patients with deleterious germline or somatic HRR gene mutations or other genes related to niraparib or AA. The research may begin at any time during the study or the post-study storage period.

Stored samples will be coded throughout the sample storage and analysis process and will not be labeled with personal identifiers. Participants may withdraw their consent for their samples to be stored for research (refer to Section 7.2.1, Withdrawal From the Use of Research Samples).

COMMITTEES STRUCTURE

Independent Data Monitoring Committee

A IDMC will be established to monitor data on an ongoing basis to review interim data, to ensure the continuing safety of the participants enrolled in this study, and to meet efficacy objectives. This committee will consist of at least one medical expert in the relevant therapeutic area and at least one statistician; committee membership responsibilities, authorities, and procedures will be documented in its charter.

PUBLICATION POLICY/DISSEMINATION OF CLINICAL STUDY DATA

All information, including but not limited to information regarding niraparib/AA FDC or the sponsor's operations (eg, patent application, formulas, manufacturing processes, basic scientific data, prior clinical data, formulation information) supplied by the sponsor to the investigator and not previously published, and any data, including pharmacogenomic or exploratory biomarker research data, generated as a result of this study, are considered confidential and remain the sole property of the sponsor. The investigator agrees to maintain this information in confidence and use this information only to accomplish this study and will not use it for other purposes without the sponsor's prior consent.

The investigator understands that the information developed in the study will be used by the sponsor in connection with the continued development of niraparib and AA and thus may be disclosed as required to other clinical investigators or regulatory agencies. To permit the information derived from the clinical studies to be used, the investigator is obligated to provide the sponsor with all data obtained in the study.

The results of the study will be reported in a Clinical Study Report generated by the sponsor and will contain data from all study sites that participated in the study as per protocol. Recruitment performance or specific expertise related to the nature and the key assessment parameters of the study will be used to determine a coordinating investigator for the study. Results of pharmacogenomic and exploratory biomarker analyses performed after the Clinical Study Report has been issued will be reported in a separate report and will not require a revision of the Clinical Study Report.

Study participant identifiers will not be used in publication of results. Any work created in connection with performance of the study and contained in the data that can benefit from copyright protection (except any publication by the investigator as provided for below) shall be the property of the sponsor as author and owner of copyright in such work.

Consistent with Good Publication Practices and International Committee of Medical Journal Editors (ICMJE) guidelines, the sponsor shall have the right to publish such primary (multicenter) data and information without approval from the investigator. The investigator has the right to publish study site-specific data after the primary data are published. If an investigator wishes to publish information from the study, a copy of the manuscript must be provided to the sponsor for review at least 60 days before submission for publication or presentation. Expedited reviews will be arranged for abstracts, poster presentations, or other materials. If requested by the sponsor in writing, the investigator will withhold such publication for up to an additional 60 days to allow for filing of a patent application. In the event that issues arise regarding scientific integrity or regulatory compliance, the sponsor will review these issues with the investigator. The sponsor will not mandate modifications to scientific content and does not have the right to suppress information. For multicenter study designs and sub-study approaches, secondary results generally should not be published before the primary endpoints of a study have been published. Similarly, investigators will recognize the integrity of a multicenter study by not submitting for publication data derived from the individual study site until the combined results from the completed study have been

submitted for publication, within 18 months after the study end date, or the sponsor confirms there will be no multicenter study publication. Authorship of publications resulting from this study will be based on the guidelines on authorship, such as those described in the ICMJE Recommendations for the Conduct, Reporting, Editing and Publication of Scholarly Work in Medical Journals, which state that the named authors must have made a significant contribution to the conception or design of the work; or the acquisition, analysis, or interpretation of the data for the work; and drafted the work or revised it critically for important intellectual content; and given final approval of the version to be published; and agreed to be accountable for all aspects of the work in ensuring that questions related to the accuracy or integrity of any part of the work are appropriately investigated and resolved.

Registration of Clinical Studies and Disclosure of Results

The sponsor will register and disclose the existence of and the results of clinical studies as required by law. The disclosure of the final study results will be performed after the end of study in order to ensure the statistical analyses are relevant.

DATA QUALITY ASSURANCE

Data Quality Assurance/Quality Control

Steps to be taken to ensure the accuracy and reliability of data include the selection of qualified investigators and appropriate study sites, review of protocol procedures with the investigator and study-site personnel before the study, periodic monitoring visits by the sponsor, and direct transmission of clinical laboratory data from a central laboratory into the sponsor's database. Written instructions will be provided for collection, handling, storage, and shipment of samples.

Guidelines for eCRF completion will be provided and reviewed with study-site personnel before the start of the study. The sponsor will review eCRF for accuracy and completeness during on-site monitoring visits and after transmission to the sponsor; any discrepancies will be resolved with the investigator or designee, as appropriate. After upload of the data into the study database they will be verified for accuracy and consistency with the data sources.

CASE REPORT FORM COMPLETION

Case report forms are prepared and provided by the sponsor for each participant in electronic format. All data relating to the study must be recorded in eCRF. All eCRF entries, corrections, and alterations must be made by the investigator or authorized study-site personnel. The investigator must verify that all data entries in the eCRF are accurate and correct.

The study data will be transcribed by study-site personnel from the source documents onto an eCRF, if applicable. Study-specific data will be transmitted in a secure manner to the sponsor.

Worksheets may be used for the capture of some data to facilitate completion of the eCRF. Any such worksheets will become part of the participant's source documents. Data must be entered into eCRF in English. The eCRF must be completed as soon as possible after a participant visit and the forms should be available for review at the next scheduled monitoring visit.

All participative measurements (eg, pain scale information or other questionnaires) will be completed by the same individual who made the initial baseline determinations whenever possible.

If necessary, queries will be generated in the electronic Data Capture (eDC) tool. If corrections to a eCRF are needed after the initial entry into the eCRF, this can be done in either of the following ways:

- Investigator and study-site personnel can make corrections in the eDC tool at their own initiative or as a response to an auto query (generated by the eDC tool).
- Sponsor or sponsor delegate can generate a query for resolution by the investigator and study-site personnel.

SOURCE DOCUMENTS

At a minimum, source documents consistent in the type and level of detail with that commonly recorded at the study site as a basis for standard medical care must be available for the following: participant identification, eligibility, and study identification; study discussion and date informed consent was obtained; dates of visits; results of safety and efficacy parameters as required by the protocol; record of all adverse events and follow-up of adverse events; concomitant medication; study medication receipt/dispensing/return records; study medication administration information; and date of study completion and reason for early discontinuation of study medication or withdrawal from the study, if applicable.

The author of an entry in the source documents should be identifiable.

Specific details required as source data for the study and source data collection methods will be reviewed with the investigator before the study and will be described in the monitoring guidelines (or other equivalent document).

The minimum source documentation requirements for Section 5.1, Inclusion Criteria and Section 5.2, Exclusion Criteria that specify a need for documented medical history are as follows:

- Referral letter from treating physician or
- Complete history of medical notes at the site
- Discharge summaries

Inclusion and exclusion criteria not requiring documented medical history must be verified at a minimum by participant interview or other protocol required assessment (eg, physical examination, laboratory assessment) and documented in the source documents.

An eSource system may be utilized, which contains data traditionally maintained in a hospital or clinic record to document medical care (eg, electronic source documents) as well as the clinical study-specific data fields as determined by the protocol. This data is electronically extracted for use by the sponsor. If eSource is utilized, references made to the eCRF in the protocol include the eSource system but information collected through eSource may not be limited to that found in the eCRF.

MONITORING

The sponsor will perform on-site monitoring visits as frequently as necessary. The monitor will record dates of the visits in a study site visit log that will be kept at the study site. The first post-initiation visit will be made as soon as possible after enrollment has begun. At these visits, the monitor will compare the data entered into the eCRF with the source documents (eg, hospital/clinic/physician's office medical records). The nature and location of all source documents will be identified to ensure that all sources of original data required to complete the eCRF are known to the sponsor and study-site personnel and are accessible for verification by the sponsor study-site contact. If electronic records are maintained at the study site, the method of verification must be discussed with the study-site personnel.

Direct access to source documents (medical records) must be allowed for the purpose of verifying that the recorded data are consistent with the original source data. Findings from this review will be discussed with the study-site personnel. The sponsor expects that, during monitoring visits, the relevant study-site personnel will be available, the source documents will be accessible, and a suitable environment will be provided for review of study-related documents. The monitor will meet with the investigator on a regular basis during the study to provide feedback on the study conduct.

In addition to on-site monitoring visits, remote contacts can occur. It is expected that during these remote contacts, study-site personnel will be available to provide an update on the progress of the study at the site.

ON-SITE AUDITS

Representatives of the sponsor's clinical quality assurance department may visit the study site at any time during or after completion of the study to conduct an audit of the study in compliance with regulatory guidelines and company policy. These audits will require access to all study records, including source documents, for inspection. Participant privacy must, however, be respected. The investigator and study-site personnel are responsible for being present and available for consultation during routinely scheduled study-site audit visits conducted by the sponsor or its designees.

Similar auditing procedures may also be conducted by agents of any regulatory body, either as part of a national GCP compliance program or to review the results of this study in support of a regulatory submission. The investigator should immediately notify the sponsor if he or she has been contacted by a regulatory agency concerning an upcoming inspection.

RECORD RETENTION

In compliance with the ICH/GCP guidelines, the investigator/institution will maintain all eCRF and all source documents that support the data collected from each participant, as well as all study documents as specified in ICH/GCP Section 8, Essential Documents for the Conduct of a Clinical Trial, and all study documents as specified by the applicable regulatory requirement(s). The investigator/institution will take measures to prevent accidental or premature destruction of these documents.

Essential documents must be retained until at least 2 years after the last approval of a marketing application in an ICH region and until there are no pending or contemplated marketing applications in an ICH region or until at least 2 years have elapsed since the formal discontinuation of clinical development of the investigational product. These documents will be retained for a longer period if required by the applicable regulatory requirements or by an agreement with the sponsor. It is the responsibility of the sponsor to inform the investigator/institution as to when these documents no longer need to be retained.

If the responsible investigator retires, relocates, or for other reasons withdraws from the responsibility of keeping the study records, custody must be transferred to a person who will accept the responsibility. The sponsor must be notified in writing of the name and address of the new custodian. Under no circumstance shall the investigator relocate or dispose of any study documents before having obtained written approval from the sponsor.

If it becomes necessary for the sponsor or the appropriate regulatory authority to review any documentation relating to this study, the investigator/institution must permit access to such reports.

STUDY AND SITE START AND CLOSURE

First Act of Recruitment

The first site open is considered the first act of recruitment and it becomes the study start date.

Study Termination

The sponsor reserves the right to close the study site or terminate the study at any time for any reason at the sole discretion of the sponsor. Study sites will be closed upon study completion. A study site is considered closed when all required documents and study supplies have been collected and a study-site closure visit has been performed.

The investigator may initiate study-site closure at any time, provided there is reasonable cause and sufficient notice is given in advance of the intended termination.

Reasons for the early closure of a study site by the sponsor or investigator may include but are not limited to:

- Failure of the investigator to comply with the protocol, the requirements of the IEC/IRB or local health authorities, the sponsor's procedures, or GCP guidelines
- Inadequate recruitment of participants by the investigator
- Discontinuation of further study medication development

10.3. Appendix 3: Additional Information on CYP450 Drug Interactions

http://www.fda.gov/drugs/developmentapprovalprocess/developmentresources/druginteractionslabeling/ucm093664.htm.[19]

http://medicine.iupui.edu/clinpharm/ddis/table.aspx

10.4. Appendix 4: Adverse Events, Serious Adverse Events, Product Quality Complaints, and Other Safety Reporting: Definitions and Procedures for Recording, Evaluating, Follow-up, and Reporting

ADVERSE EVENT DEFINITIONS AND CLASSIFICATIONS

Adverse Event

An adverse event is any untoward medical occurrence in a clinical study participant administered a pharmaceutical (investigational or non-investigational) product. An adverse event does not necessarily have a causal relationship with the study medication. An adverse event can therefore be any unfavorable and unintended sign (including an abnormal finding), symptom, or disease temporally associated with the use of a medicinal (investigational or non-investigational) product, whether or not related to that medicinal (investigational or non-investigational) product. (Definition per ICH).

This includes any occurrence that is new in onset or aggravated in severity or frequency from the baseline condition, or abnormal results of diagnostic procedures, including laboratory test abnormalities.

Note: The sponsor collects adverse events starting with obtaining informed consent (refer to All Adverse Events under Section 8.3.1, Time Period and Frequency for Collecting Adverse Events and Serious Adverse Events Information, for time of last adverse event recording).

Serious Adverse Event

A serious adverse event based on ICH and EU Guidelines on Pharmacovigilance for Medicinal Products for Human Use is any untoward medical occurrence that at any dose:

- Results in death
- Is life-threatening
  (The participant was at risk of death at the time of the event. It does not refer to an event that hypothetically might have caused death if it were more severe.)
- Requires inpatient hospitalization or prolongation of existing hospitalization
- Results in persistent or significant disability/incapacity
- Is a congenital anomaly/birth defect
- Is a suspected transmission of any infectious agent via a medicinal product
- Is Medically Important*

*Medical and scientific judgment should be exercised in deciding whether expedited reporting is also appropriate in other situations, such as important medical events that may not be immediately life threatening or result in death or hospitalization but may jeopardize the participant or may require intervention to prevent one of the other outcomes listed in the definition above. These should usually be considered serious.

Unlisted (Unexpected) Adverse Event/Reference Safety Information

An adverse event is considered unlisted if the nature or severity is not consistent with the applicable product reference safety information. For niraparib/AA FDC, the expectedness of an adverse event will be determined by whether or not it is listed in the niraparib, AA or FDC IBs. For single agent niraparib, the expectedness of an adverse event will be determined by whether or not it is listed in the IB. For single agent AA, the expectedness of an adverse event will be determined by whether or not it is listed in the IB.

ATTRIBUTION DEFINITIONS

Assessment of Causality

The causal relationship to study medication is determined by the Investigator. The following selection should be used to assess all adverse events (AE).

Related

There is a reasonable causal relationship between study medication administration and the AE.

Not Related

There is not a reasonable causal relationship between study medication administration and the AE.

The term "reasonable causal relationship" means there is evidence to support a causal relationship.

SEVERITY CRITERIA

An assessment of severity grade will be made using the NCI-CTCAE (Version 5.0). Any AE not listed in the NCI-CTCAE will be graded according to the investigator's clinical judgment using the standard grades as follows:

Grade 1 (Mild): Awareness of symptoms that are easily tolerated, causing minimal discomfort and not interfering with everyday activities.

Grade 2 (Moderate): Sufficient discomfort is present to cause interference with normal activity.

Grade 3 (Severe): Extreme distress, causing significant impairment of functioning or incapacitation. Prevents normal everyday activities.

Grade 4, Life-threatening: Urgent intervention indicated.

Grade 5, Death: Death.

The investigator should use clinical judgment in assessing the severity of events not directly experienced by the participant (eg, laboratory abnormalities).

SPECIAL REPORTING SITUATIONS

Safety events of interest on a sponsor study medication in an interventional study that may require expedited reporting or safety evaluation include, but are not limited to:

- Overdose of a sponsor study medication
- Suspected abuse/misuse of a sponsor study medication
- Accidental or occupational exposure to a sponsor study medication
- Medication error, intercepted medication error, or potential medication error involving a Johnson & Johnson medicinal product (with or without patient exposure to the Johnson & Johnson medicinal product, eg, product name confusion, product label confusion, intercepted prescribing or dispensing errors)

Special reporting situations should be recorded in the eCRF. Any special reporting situation that meets the criteria of a serious adverse event should be recorded on the serious adverse event page of the eCRF.

PROCEDURES

All Adverse Events

All adverse events, regardless of seriousness, severity, or presumed relationship to study medication, must be recorded using medical terminology in the source document and the eCRF. Whenever possible, diagnoses should be given when signs and symptoms are due to a common etiology (eg, cough, runny nose, sneezing, sore throat, and head congestion should be reported as "upper respiratory infection"). Investigators must record in the eCRF their opinion concerning the relationship of the adverse event to study therapy. All measures required for adverse event management must be recorded in the source document and reported according to sponsor instructions.

For all studies with an outpatient phase, including open-label studies, the participant must be provided with a "wallet (study) card" and instructed to carry this card with them for the duration of the study indicating the following:

- Study number
- Statement, in the local language(s), that the participant is participating in a clinical study
- Investigator's name and 24-hour contact telephone number
- Local sponsor's name and 24-hour contact telephone number (for medical personnel only)
- Site number
- Participant number
- Any other information that is required to do an emergency breaking of the blind

Serious Adverse Events

All serious adverse events that have not resolved by the end of the study, or that have not resolved upon discontinuation of the participant's participation in the study, must be followed until any of the following occurs:

- The event resolves

- The event stabilizes
- The event returns to baseline, if a baseline value/status is available
- The event can be attributed to agents other than the study medication or to factors unrelated to study conduct
- It becomes unlikely that any additional information can be obtained (participant or health care practitioner refusal to provide additional information, lost to follow-up after demonstration of due diligence with follow-up efforts)

Suspected transmission of an infectious agent by a medicinal product will be reported as a serious adverse event. Any event requiring hospitalization (or prolongation of hospitalization) that occurs during the course of a participant's participation in a study must be reported as a serious adverse event, except hospitalizations for the following:

- Hospitalizations not intended to treat an acute illness or adverse event (eg, social reasons such as pending placement in long-term care facility)
- Surgery or procedure planned before entry into the study (must be documented in the eCRF).

Expected progression of disease should not be considered an adverse event (or serious adverse event). However, if determined by the investigator to be more likely related to the study medication than the underlying disease, the clinical signs or symptoms of progression and the possibility that the study medication is enhancing disease progression, should be reported per the usual reporting requirements.

During the Follow-up Phase of the study, deaths regardless of causality will be reported in the eCRF. Serious AEs, including those spontaneously reported to the investigator within 30 days after the last dose of study medication, must be reported using the Serious Adverse Event Form. Serious AEs that occur after 30 days following the last study medication administration thought to be related to study medication will be collected and reported via the Serious Adverse Event Form within 24 hours of discovery or notification of the event and documented.

CONTACTING SPONSOR REGARDING SAFETY

The names (and corresponding telephone numbers) of the individuals who should be contacted regarding safety issues or questions regarding the study are listed in the Contact Information page(s), which will be provided as a separate document.

PRODUCT QUALITY COMPLAINT HANDLING

A product quality complaint (PQC) is defined as any suspicion of a product defect related to manufacturing, labeling, or packaging, ie, any dissatisfaction relative to the identity, quality, durability, reliability, or performance of a distributed product, including its labeling, drug delivery system, or package integrity. A PQC may have an impact on the safety and efficacy of the product.. In addition, it includes any technical complaints, defined as any complaint that indicates a potential quality issue during manufacturing, packaging, release testing, stability monitoring, dose preparation, storage or distribution of the product or the drug delivery system.

Procedures

All initial PQCs must be reported to the sponsor by the study-site personnel within 24 hours after being made aware of the event.

A sample of the suspected product should be maintained under the correct storage conditions until a shipment request is received from the sponsor.

Contacting Sponsor Regarding Safety, Including Product Quality

The names (and corresponding telephone numbers) of the individuals who should be contacted regarding safety issues, PQC, or questions regarding the study are listed in the Contact Information page(s), which will be provided as a separate document.

10.5. Appendix 5: Guidance on Study Conduct During the COVID-19 Pandemic

It is recognized that the Coronavirus Disease 2019 (COVID-19) pandemic may have an impact on the conduct of this clinical study due to, for example, self-isolation/quarantine by participants and study-site personnel; travel restrictions/limited access to public places, including hospitals; and study site personnel being reassigned to critical clinical tasks.

In alignment with recent health authority guidance, the sponsor is providing options for study-related participant management in the event of disruption to the conduct of the study. This guidance does not supersede any local or government requirements or the clinical judgement of the investigator to protect the health and well-being of participants and site staff. If at any time a participant's safety is considered to be at risk, study medication will be discontinued, and study follow-up will be conducted.

Scheduled visits that cannot be conducted in person at the study site may be performed to the extent possible remotely/virtually or delayed until such time that on-site visits can be resumed. At each contact, participants should be interviewed to collect safety data. Key efficacy endpoint assessments should be performed if required and as feasible. Participants will also be questioned regarding general health status to fulfill any physical examination requirement.

Every effort should be made to adhere to protocol-specified assessments for participants on study medication, including follow-up. Modifications to protocol-required assessments may be permitted via COVID-19 Appendix after consultation with the participant, investigator, and the sponsor. Examples of modifications are presented under the GUIDANCE SPECIFIC TO THIS PROTOCOL. Missed assessments/visits will be captured in the clinical trial management system for protocol deviations. Discontinuations of study medications and withdrawal from the study should be documented with the prefix "COVID-19-related" in the CRF.

The sponsor will continue to monitor the conduct and progress of the clinical study, and any changes will be communicated to the sites and to the health authorities according to local guidance. If a participant has tested positive for COVID-19, the investigator should contact the sponsor's responsible medical officer to discuss plans for study medication and follow-up. Modifications made to the study conduct as a result of the COVID-19 pandemic should be summarized in the clinical study report.

GUIDANCE SPECIFIC TO THIS PROTOCOL

These provisions are meant to minimize the risk of exposure to COVID-19 and to safely maintain participants on study medication while site capabilities are compromised by COVID-19-related restrictions. As restrictions are lifted and the acute phase of the COVID-19 pandemic resolves, sites should revert to original protocol conduct as soon as feasible.

Study Visits - General

- To protect study participants from being unnecessarily exposed to COVID-19, at sites that have been affected, those participants who are doing well and for whom there is no safety concern may have home health or tele-health visits (conducted via phone or computer). Normal

study procedures (eg, Eastern Cooperative Oncology Group assessment, adverse event reporting) should be followed for the applicable visit as possible. Regular blood pressure monitoring can be performed at home, if possible. Other programs may be implemented by or with approval from the sponsor, such as home health care visits for study assessments and procedures (eg, physical examination, lab draws, etc), where feasible and permissible by local policy and regulations.

- The benefit of continuing treatment should be balanced with the risks of treatment without the usual clinical supervision. For participants who have recently had adverse events that required a study medication interruption or dose reduction, or for those participants who are at increased risk for severe COVID-19 infection based on age or comorbidities, a temporary interruption of study medication should be considered by the investigator and documented in the source and electronic CRF (eCRF).

Prescreening, Screening and Randomization

- Prescreening, screening, and randomization to the study may continue during the COVID-19 pandemic if the benefits of study medication outweigh the risks, as per the investigator's assessment. If it is anticipated that the situation may worsen at the site and that health care capacities may become more limited, then screening and randomization should be delayed for participant safety. When randomization occurs at the site, the screening procedures may be utilized after discussion with the sponsor, despite being out of window.

Study Visits – Assessments

- If possible, central safety laboratory testing is to be continued. However, if central laboratory tests cannot be performed, the use of a local laboratory is allowed for study evaluations. A copy of the local laboratory report should be reviewed by the investigator and retained, along with the reference ranges, for the source documentation and documented in the eCRF. If research-specific blood collection is not possible (eg, biomarker, pharmacokinetic collection) then the missed sample should be documented as a deviation.
- If visits are conducted remotely, then electronic patient-reported outcomes information should be collected via phone using interview mode on the site tablet or web-based platform available to participants.
- If safe to do so, then participants should come to their usual site for imaging visits. If imaging cannot be performed at the site due to travel restrictions, the use of a local facility close to the participant's home is permitted, but provision of scans to the investigative site should be arranged and documented. If no imaging is possible, the missed visit should be documented as a deviation, and imaging should be resumed when possible.

Study Medication Supply

- For participants able to visit the clinic/hospital, but who request to reduce visit frequency, an additional supply of study medications can be provided.
- For participants unable to visit the clinic/hospital, direct-to-patient (DTP) shipment of study medications may be implemented, where allowed per local regulations and if requested by the treating study physician. Where DTP shipments are deemed necessary, the process should be

coordinated between the site and sponsor staff following standard DTP procedures for arranging shipment and adhering to associated approvals and documentation requirements.

Exposure to COVID-19

- If a participant develops COVID-19 infection, niraparib and AA, plus prednisone or AA plus prednisone should be interrupted until the infection resolves.
- Resumption of treatment: When a participant recovers from suspected or confirmed COVID-19 infection, niraparib and AA, plus prednisone or AA plus prednisone may be resumed when safe to so as determined by the study physician in consultation with the sponsor prior to resuming.
- COVID-19 infection should be reported to the sponsor and follow the usual Adverse Event/Serious Adverse Event reporting requirements.

Reporting of Protocol Deviations

- All deviations from the study protocol will be reported based on standard guidance. If related to the COVID-19 pandemic situation, these deviations will be recorded as COVID-19 related by the sponsor.

COVID vaccine

- Subjects eligible for COVID vaccination should be vaccinated with non-live vaccines (mRNA or non-replicating viral shell). Based on available information, there is no impact on the safety or physical or mental integrity of the clinical study participants, or the scientific value of the study. Please note that the type of vaccine, dates of first and second vaccinations, the doses used, and any adverse events should also be recorded.
- Note: administration of non-live vaccines approved or authorized for emergency use (eg, COVID 19) by local health authorities are allowed before or during this study. For guidance on vaccination, please refer to recommendations for oncology study participants.[13,22,38]

10.6. Appendix 6: ECOG Performance Status

ECOG Grade	Scale (with Karnofsky conversion)
0	Fully active, able to carry on all predisease performance without restriction. (Karnofsky 90-100)
1	Restricted in physically strenuous activity but ambulatory and able to carry out work on a light or sedentary nature, eg, light housework, office work. (Karnofsky 70-80)
2	Ambulatory and capable of all self-care but unable to carry out any work activities. Up and about more than 50% of waking hours. (Karnofsky 50-60)
3	Capable of only limited self-care; confined to bed or chair more than 50% of waking hours. (Karnofsky 30-40)
4	Completely disabled. Cannot carry on any self-care. Totally confined to bed or chair. (Karnofsky 10-20)
5	Dead. (Karnofsky 0)

10.7. Appendix 7: Clinical Laboratory Tests

The following tests will be performed according to the Schedule of Activities typically by a central laboratory. Local laboratory tests may be performed as necessary.

Protocol-Required Safety Laboratory Assessments

Laboratory Assessments	Parameters
Hematology[a]	• Platelet count<br>• Red blood cell count<br>• Hemoglobin<br>• White blood cell count with differential<br>• Absolute neutrophil count<br>• Absolute lymphocyte count
Clinical Chemistry[a]	• Potassium<br>• Creatinine<br>• Glucose<br>• Lactate dehydrogenase<br>• Albumin
Liver Function Tests[a]	• Aspartate aminotransferase (AST)/Serum glutamic-oxaloacetic transaminase<br>• Alanine aminotransferase (ALT)/Serum glutamic-pyruvic transaminase<br>• Total bilirubin (if above normal, measure direct bilirubin)<br>• Alkaline phosphatase
Other tests	• Prostate-specific antigen (PSA)<br>• International normalized ratio (INR)<br>• Prothrombin time (PT)<br>• Activated partial thromboplastin time (aPTT)

[a] Safety labs must be obtained within 14 days of randomization to ensure patient still meets eligibility criteria. Screening labs can be used if they are within this window. Starting from C1D1, laboratory assessments may be performed up to 3 days prior to study visit. If screening laboratory safety assessments are done within 3 days of C1D1, no need to repeat on C1D1.

10.8. Appendix 8: Anticipated Adverse Events

An anticipated event is an adverse event (serious or nonserious) that commonly occurs as a consequence of the underlying disease or condition under investigation (disease-related) or background regimen. For the purposes of this study, the following events will be considered anticipated events:

**Disease-specific Events**	**ADT Events**
erectile dysfunction	depression
hematuria	gynecomastia
incontinence	libido decreased
lymphoedema	osteoporosis
nocturia	sexual dysfunction
painful ejaculation	testicular atrophy
prostatic specific antigen increased	
urinary flow decreased	
urinary hesitation	
urinary tract obstruction	

Reporting of Anticipated Events

All AEs will be recorded in the eCRF regardless of whether considered to be anticipated events and will be reported to the sponsor as described in Section 10.4 Appendix 4 Adverse Events: Definitions and Procedures for Recording, Evaluating, Follow-Up, and Reporting. Any anticipated event that meets serious criteria will be reported to the sponsor as described in Section 10.4 Appendix 4 Adverse Events: Definitions and Procedures for Recording, Evaluating, Follow-Up, and Reporting. Each anticipated event will be assessed by the investigator at the individual case level and if considered to be drug-related will undergo expedited reporting (if appropriate) per applicable clinical trial legislation to Health Authorities and IRB/ECs If an anticipated event is considered disease-related or not related to study drug the event will be exempt from expedited reporting.

To meet US regulatory clinical trial legislation, the sponsor will perform aggregate review of anticipated events as outlined below, and if determined to be drug-related will implement expedited reporting of these events to Health Authorities and IRBs/ECs. If an interim analysis of trial results

leads to an unblinded, aggregate review of safety data by the study team, the sponsor may terminate the review of pre-specified anticipated events outlined above.

Safety Assessment Committee (SAC)

A Safety Assessment Committee (SAC) will be established to perform reviews of pre-specified anticipated events at an aggregate level. The SAC is a safety committee within the sponsor's organization that is independent of the sponsor's study team. The SAC will meet to aid in the recommendation to the sponsor's study team as to whether there is a reasonable possibility that an anticipated event is related to the study medication based on a review of the aggregate data by arm.

Statistical Analysis

Details of statistical analysis of anticipated events, including the frequency of review and threshold to trigger an aggregate analysis of anticipated events will be provided in a separate Anticipated Events Safety Monitoring Plan.

10.9. Appendix 9: Child-Pugh Criteria

Clinical and Lab Criteria	Points*		
	1	2	3
Encephalopathy	None	Mild to moderate (grade 1 or 2)	Severe (grade 3 or 4)
Ascites	None	Mild to moderate (diuretic responsive)	Severe (diuretic refractory)
Bilirubin (mg/dL)	< 2	2-3	>3
Albumin (g/dL)	> 3.5	2.8-3.5	<2.8
Prothrombin time			
Seconds prolonged	<4	4-6	>6
International normalized ratio	<1.7	1.7-2.3	>2.3
**Child-Turcotte-Pugh Class obtained by adding score for each parameter (total points)**<br>Class A = 5 to 6 points (least severe liver disease)<br>Class B = 7 to 9 points (moderately severe liver disease)<br>Class C = 10 to 15 points (most severe liver disease)			

10.10. Appendix 10: Open-label Extension Phase

The Open-label Extension (OLE) Phase may be initiated when at least Amendment 2 is approved at the site and upon notification from the sponsor to unblind subjects and initiate the start of the OLE. The purpose of the OLE Phase is to allow subjects to receive open-label study treatment. Hereafter the term "study treatment" refers to either FDC, niraparib and/or AAP drug formulations as relevant to the subject entering OLE.

Subjects who are participating in the Treatment Phase of the study and who are still on study treatment will be offered the option to receive open-label study treatment if they meet the eligibility criteria below. Eligible subjects who were randomized to placebo+AAP may be offered the option to receive open-label niraparib+AAP (investigators can choose to maintain subjects on AAP alone). Eligible subjects who were randomized to niraparib+AAP may continue open-label study treatment.

Subjects with a study drug interruption at the time of entry to OLE due to an adverse event should continue to have their study treatment held until resolution of the adverse event and the investigator should discuss with the sponsor the appropriate dose of niraparib and/or AAP to be administered in the OLE Phase. If the subject has study drug withheld due to an adverse event, all 4 eligibility criteria listed below would need to be met for the subject to start study treatment in the OLE Phase.

Subjects who discontinued either niraparib or AAP due to an adverse event may continue to receive the same study treatment that they are currently taking if they meet eligibility criteria 1 to 3 listed below.

Subjects who have already ended study treatment and are in the Follow-up Phase of the main study may not resume study treatment and may be followed for select study endpoints such as survival.

Subjects will be followed per the schedule of procedures provided in the table below in this appendix until a reason for discontinuation of study treatment has been met (see protocol Section 7.1), the sponsor initiates the Long-term Extension (LTE) Phase, or until further notification by the sponsor of a different means for continued supply of study treatment, whichever occurs first.

Subjects in OLE may be moved to LTE if sponsor approval was obtained (Appendix 11: Long-term Extension Phase).

Eligibility Criteria for Subjects who cross-over to OLE treatment after Unblinding

Subjects eligible to receive either open-label study treatment should meet the criteria below within 28 days prior to starting OLE treatment. Re-evaluation of out-of-range criteria may be permitted after discussion with the sponsor.

1. Still participating in the Treatment Phase of the study
2. Willing and able to provide informed consent to participate in the OLE Phase

3. Not have niraparib or FDC withheld for ≥3 consecutive months due to an adverse event in the double-blind Treatment Phase immediately prior to OLE Phase consent (not applicable for those continuing AAP only in OLE)
4. Subjects crossing-over to open-label niraparib+AAP from placebo+AAP and those entering OLE after having study treatment held for an adverse event should have adequate organ function and performance status as outlined in the original protocol (Section 5.1 Criteria 6 and 11). All other subjects entering OLE may continue with organ function testing and management with adverse events according to where they fall in the Schedule of Activities (Table 2) from the Treatment Phase. Additional details are provided in the schedule of procedures in the table below in this appendix.

Testing through local and central laboratories are permitted for OLE eligibility. After eligibility, only local laboratory testing should be performed.

Study Treatment Administration

Open-label study treatment

Study treatment will be taken orally on an outpatient basis and a treatment cycle is defined as 28 days. Sufficient study drug for each treatment cycle will be distributed as specified in Section 6.1.2 of the protocol. Dose modifications for toxicity are provided in Section 6.5 of the protocol.

Subjects on a lower dose of study treatment in the Treatment Phase of the study should continue the same dose during the OLE Phase unless approved in advance with the sponsor’s medical monitor (see protocol Section 6.5).

Androgen Deprivation Therapy (ADT) Administration

ADT should be continued as per local practice.

Prohibitions and Restrictions

Refer to protocol Sections 6.8.2 and 6.8.3.

Study Procedures for the Open-label Extension

Subjects who meet the eligibility criteria as outlined above will be permitted to start open-label study treatment. Cycle 1 Day 1 of the OLE should follow the last cycle in the double-blind Treatment Phase for those crossing over to open-label niraparib+AAP from placebo+AAP. Subjects who enter OLE continuing the same study treatment they received in the Treatment Phase (ie, niraparib+AAP, niraparib only, or AAP only) will continue with cycles numbered sequentially from the last cycle received while in the main study (ie, if the last cycle in the Treatment Phase was Cycle 15, the first OLE cycle will be Cycle 16). Subjects should follow the schedule of procedures provided in the table below in this appendix.

Blood samples for serum chemistry and hematology should be collected from a local laboratory as specified in the table below in this appendix. The investigator should document review of the laboratory results and record clinically relevant changes in the AE section of the eCRF (eg, laboratory abnormalities leading to dose interruptions, dose changes, or permanent study drug withdrawal). Unscheduled laboratory assessments may be performed as required.

Time and Events Schedule (Open-Label Extension)[a]

Procedures	Notes	Subjects crossing over to open-label niraparib+AAP after receiving placebo+AAP in the Treatment Phase	Subjects continuing the same study treatment they received in Treatment Phase (niraparib+AAP, niraparib only, or AAP only)	Subjects off Study Treatment
Each treatment cycle is 28 days Visit window during the OLE Phase is ±1 week.				
**Screening**				
Informed consent for the OLE Phase		X		
Eligibility		X		
**Study Drug Dispensing**				
niraparib+AAP (FDC), AAP, or niraparib alone		Continuous; sufficient study drug until next visit will be dispensed		
Study drug compliance		Bottle(s) including any unused medication will be returned at every visit		
**Clinical Laboratory**				
Safety Labs[b,g]	See Footnote b for required laboratory tests.	See Footnote c for frequency of required laboratory tests	Continue monitoring schedule following SoA (Table 2) for Hematology and Potassium, Chemistry, and LFTs	
**Safety**				
Physical examination		At each visit. Clinically relevant abnormalities should be reported as AEs.		
Blood pressure and Heart rate		See Footnote h for frequency of required blood pressure and heart rate	Continue monitoring schedule following SoA (Table 2) for Blood pressure and Heart rate	
SAE/AE		X[d]		
Concomitant therapy		X[d]		
Survival	May be obtained by telephone or chart review	X		X[e]
**Efficacy**				
Tumor assessments,[f] PSA	As per local practice			

AA abiraterone acetate; AAP abiraterone acetate plus prednisone or prednisolone; AE adverse event; FDC fixed dose combination; LFT liver function test; OLE Open label Extension; PSA prostate specific antigen; SAE serious adverse event; SoA Schedule of Activities.

a. After Final Analysis, the OLE Time and Event Schedule should be used as a recommended guideline to monitor patient safety. Data will not be collected on any assessments by the sponsor other than SAEs.
b. Hematology, Clinical Chemistry, and LFTs as defined in Appendix 7: Clinical Laboratory Tests.
c. Hematology panel and potassium should be performed weekly for Cycle 1 of treatment, Day 1 and 15 for Cycles 2 and 3, Day 1 of each cycle starting Cycle 4 until Cycle 25, then every 4 cycles. Chemistry panel should be performed Day 1 of each cycle until Cycle 25, then every 4 cycles. LFTs should be performed on Day 1 and 15 for the first 3 cycles of treatment, Day 1 of each cycle starting Cycle 4 until Cycle 25, then every 4 cycles.
d. Continuous during this period and until 30 days after the last dose if discontinued during this period.
e. Every 4 months until notified by the sponsor to stop collection. Other secondary and exploratory endpoints may be collected during follow up as part of survival.
f. Investigator disease assessments only. No central review of imaging during OLE.
g. Local testing only, no central laboratory testing during OLE.
h. Blood pressure and heart rate should be obtained weekly for Cycle 1 and Cycle 2, Day 1 and 15 for Cycle 3, Day 1 of each cycle starting Cycle 4 until Cycle 25, then every 4 cycles.

10.11. Appendix 11: Long-term Extension Phase

The LTE Phase will be initiated when at least Amendment 2 is approved at the site and upon notification from the sponsor to start LTE. The purpose of the LTE Phase is to provide study treatment to subjects, while minimizing the burden of data collection.

Subjects who are still on study treatment will be offered the option to continue in the LTE Phase if they meet the eligibility criteria below.

Subjects will continue the study treatment that they were receiving at the time of transition to LTE. Subjects who had discontinued study treatment and are in the Follow-up Phase will be discontinued from the study upon the start of the LTE.

Subjects will be followed per the schedule of procedures provided in the table below in this appendix until a reason for discontinuation of study treatment has been met (see protocol Section 7.1), or until further notification by the sponsor of a different means for continued supply of study treatment, whichever occurs first.

LTE Eligibility Criteria:

1. Still receiving study treatment
2. Willing and able to provide informed consent to participate in the LTE Phase

Study Treatment Administration

Open-label study treatment

Study treatment will be taken orally on an outpatient basis and a treatment cycle is defined as 28 days. Sufficient study drug for each treatment cycle will be distributed as specified in Section 6.1.2 of the protocol. Dose modifications for toxicity are provided in Section 6.5 of the protocol.

Subjects will continue the study treatment that they were receiving at the time of transition to LTE.

Androgen Deprivation Therapy (ADT) Administration

ADT should be continued as per local practice.

Prohibitions and Restrictions

Refer to protocol Sections 6.8.2 and 6.8.3.

Study Procedures for the Long-term Extension

All subjects continuing in the LTE will follow the schedule of procedures provided in the table below in this appendix.

Investigators should monitor and assess the subjects for response, safety, and disease progression according to routine practice and local label requirements. Timing of assessments is at the

discretion of the investigator. Data collection will be limited to SAEs, which will be reported per the SAE process up to 30 days after the last dose of study drug as specified in Section 10.4. No other safety nor any efficacy data are to be collected during the LTE; no analyses are planned for the LTE.

Based on local regulations, additional safety data may be collected.

Discontinuation Criteria for the Long-term Extension

If a subject meets the discontinuation criteria as defined in Section 7.1 of the protocol, study treatment must be discontinued. Participation in the LTE Phase may also end if the sponsor provides notification of a different means for continued supply of study treatment.

The assessments and timing are specified in the table below in this appendix:

Time and Events Schedule (Long-term Extension After Unblinding)

Procedures	Comments	LTE Phase (until reaching criteria in Section 7.1, or termination of the study)
**Informed Consent**	All subjects must sign informed consent prior to entering the LTE Phase	X
**Eligibility**		X
**Study Drug Dispensing**		
Niraparib+AAP (FDC) (or AAP alone)		Continuous; sufficient study drug until next visit will be dispensed[a]
Study drug compliance		As per local standard of care
**Clinical Laboratory (Local Laboratory)**		
Hematology and blood chemistry		Per local practice and local label for niraparib and AAP
**Safety**		
SAEs		Collection of SAEs; see Section 10.4 of protocol

AAP abiraterone acetate plus prednisone; FDC fixed dose combination; LTE Long term Extension; SAE serious adverse event.

a Maximum allowed is 4 months supply.

10.12. Appendix 12: Protocol Amendment History

The Protocol Amendment Summary of Changes Table for the current amendment is located directly before the Table of Contents (TOC).

Amendment 3 (09 February 2023)

Overall Rationale for the Amendment: To revise the sample size based on emerging data from results of clinical trials with PARP inhibitors in prostate cancer.

The changes made to the clinical protocol 67652000PCR3002 as part of Protocol Amendment 3 are listed below, including the rationale of each change and a list of all applicable sections. Changes made in previous protocol amendments are listed in Section 10.12 Appendix 12: Protocol Amendment History.

Section Number and Name	Description of Change	Brief Rationale
1.1. Synopsis (Number of Participants, Efficacy);<br>1.2. Schema;<br>4.1. Overall Design;<br>9.2. Sample Size Determination	The number of participants was updated from '788' to '692'.	To revise the sample size based on emerging data from other recent clinical trials with PARP inhibitors in prostate cancer.
1.1. Synopsis (Overall Design);<br>4.1. Overall Design;<br>8. Study Assessments and Procedures	Text added: "Patients with radiographic progression can remain on therapy if still receiving clinical benefit."	To clarify reasons for treatment discontinuation.
1.3. Schedule of Activities (SoA) (Table 1)	Text added: "if test is available from the sponsor"	To clarify circumstances when samples should be collected.
1.3. Schedule of Activities (SoA) (Table 2);<br>8. Study Assessments and Procedures;<br>8.8. Qualitative Exit Interview	Qualitative Exit Interview was added as a sub-study.	To update protocol with the addition of a Qualitative Exit Interview sub-study.
1.3. Schedule of Activities (SoA) (Table 2);<br>8.5. Genetics;<br>8.6.1.2. Plasma for DNA	Text added: "where local regulations permit"	To clarify circumstances when samples should be collected.
1.3. Schedule of Activities (SoA) (Table 2);<br>5. Study Population;<br>10.2. Appendix 2: Regulatory, Ethical, and Study Oversight Considerations (Informed Consent Process)	Prescreening eligibility criterion (#1) was modified as written informed consent is required.	To clarify written informed consent required.

Section Number and Name	Description of Change	Brief Rationale
1.3. Schedule of Activities (SoA) (Table 2); 7.1. Discontinuation of Study Medication	The term 'radiographic progression' was changed to 'investigator-assessed radiographic progression'.	To provide further clarification.
1.3. Schedule of Activities (SoA) (Table 2); 10.2. Appendix 2: Regulatory, Ethical, and Study Oversight Considerations (Informed Consent Process)	Text was added to clarify that reconsent may be obtained remotely/virtually as per sponsor procedures and in accordance with local regulations.	To clarify circumstances when other forms of reconsenting beyond in person may be permitted.
2.2.3. Summary of Available Clinical Data for Niraparib and AA Plus Prednisone in Prostate Cancer; 2.3. Benefit-Risk Assessment; 4.3. Justification for Dose	Added language regarding Study 64091742PCR3001 (MAGNITUDE) and the Phase 3 PROpel study.	Updated with emerging data from PARP inhibitor trials in prostate cancer. Also provides rationale for stopping enrollment of participants with CDK12-only alterations.
2.3. Benefit-Risk Assessment;	Text was added to indicate that participants with CDK12-only alterations are no longer being enrolled in Study 67652000PCR3002.	Updates to gene alterations permitted for eligibility allowed in prior version of the protocol and specific language is added to reflect this change for CDK12-only alterations.
5. Study Population	Figure 2 Determination of Molecular Eligibility was updated.	To clarify samples collected and testing performed.
6.5. Dose Modification	Text and table content revised. Text added to define study medication terms used in table. Dose modification text modified to allow resuming both niraparib and AA concurrently after resolution of toxicity when both had been held. Option to use AA 750 mg dose removed (also in Table 6).	To clarify dose modification guidelines.
6.5.1. Posterior Reversible Encephalopathy Syndrome (PRES)	Text added: "Patients can remain on study and continue to receive AAP."	To clarify that patients can continue to receive AAP if diagnosis of PRES is confirmed.
6.8.1. Permitted Supportive Care and Interventions	Text added: "Prednisone dose increase by 5mg/day is permitted to manage refractory mineralocorticoid related toxicities and should be documented in the study medication eCRF."	To align with the language in other documents.
8.1.1. Evaluations for Primary Efficacy Assessment	Text added: "Blinded Independent Central Review of imaging scans may be ceased upon Sponsor decision communicated to investigators by letter."	To clarify the mechanism to discontinue imaging scan submission.
8.1.2.1. Patient Reported Outcomes	Text added: "In rare circumstances, such as in case of technical issues that cannot be resolved during the visit and as a last resort where other options for electronic completion have been explored and deemed not feasible, a paper questionnaire should be completed by the participant, and submitted to	To indicate that ePRO should be completed electronically and to clarify exceptions for paper questionnaires.

Section Number and Name	Description of Change	Brief Rationale
	the vendor by site staff. Every effort should be made to have the ePRO completed by patient electronically.”	
8.6.1.2. Plasma for DNA	Text added: “Plasma samples may also be used to develop tests/assays related to niraparib and AA and prostate cancer.”	To provide information regarding how collected samples may be used.
9.2. Sample Size Determination	Text added: “Long term survival follow-up will continue until approximately [redacted] events have been observed. [redacted]	To update timing of efficacy analysis based on emerging data.
9.5. Interim Analysis for Overall Survival	The number of planned formal interim analyses was updated.	To revise the number of analyses based on emerging data.
10.1. Appendix 1	The list of abbreviations was updated.	The list is updated to include new terms and abbreviations used in revised text.
10.7. Appendix 7: Clinical Laboratory Tests	Text added: “Starting from C1D1, laboratory assessments may be performed up to 3 days prior to study visit. If screening laboratory safety assessments are done within 3 days of C1D1, no need to repeat on C1D1.”	To clarify timeline permitted for testing.
10.10. Appendix 10: Open-label Extension Phase	Footnotes added to clarify no central laboratory or central imaging assessments performed during Open-Label Extension.	To clarify how disease assessments will be performed during Open-label Extension phase.
10.12. Appendix 12: Protocol Amendment History	The protocol amendment 2 summary table was moved to Appendix 12.	To align with the template requirement.
Protocol Amendment Summary of Changes Amendment 2	In protocol amendment 2, the rationale for adding MACE in Section 8.3.7. was to address known AESIs related to abiraterone acetate and not niraparib. Therefore, MACE is not a new risk associated with study treatment.	To clarify rationale for addition of MACE in protocol amendment 2.
Throughout the protocol	Minor grammatical, formatting, or spelling changes were made.	Minor errors were noted

Amendment 2 (25 October 2021)

Overall Rationale for the Amendment: To modify eligibility criteria based on investigator feedback to facilitate enrollment, to allow additional forms of testing for HRR alterations, and to clarify toxicity management guidelines to comply with the changes requested by the health authorities.

Section Number and Name	Description of Change	Brief Rationale
Synopsis (Biomarker Evaluations); 1.3. Schedule of Activities (SOA): Table 1; 4.2. Scientific Rationale for Study Design; 5. Study Population (Figure 2 and #4); 8.6.1.2. Plasma for DNA	Additional text and footnote related to biomarker evaluations using plasma sample were added.	To clarify plasma testing for biomarker samples.
Synopsis (Pharmacogenomic [DNA] Evaluations); 8.5. Genetics	The text 'or saliva' was added.	To include saliva sample collection for pharmacogenomic evaluations.
1.3. Schedule of Activities (SOA): Table 2	The screening period was revised from ≤28 days to ≤35 days.	To allow more time for completion of screening evaluation
1.3. Schedule of Activities (SOA): Table 2	The computed tomography (CT) or magnetic resonance imaging (MRI) and Technetium-99m (^{99m}Tc) Bone Scan were scheduled to occur every 4 months in the Follow-up Phase (until radiographic progression).	To align with the text in the body of the protocol.
4.2. Scientific Rationale for Study Design	The title of Table 4 was revised.	To clarify that eligible homologous recombination repair (HRR) gene alterations will be tested during the Prescreening Phase.
5. Study Population (#3)	The text 'histologically confirmed' was deleted and 'diagnosis of 'was added.	To keep all other diagnostic methods open for diagnosis, in addition to histology, at prescreening.
5. Study Population (#4)	The text related to blood or saliva sample for HRR gene alterations status determination was deleted.	To avoid repetition.
5.1. Inclusion Criteria (#2)	The inclusion criterion was revised to add the word 'pathological diagnosis'.	To specify that participants with metastatic prostate adenocarcinoma are included in the study.
5.1. Inclusion Criteria (#4)	The inclusion criterion was revised to add text related to documentation of metastatic disease.	To clarify further the documentation of metastatic disease and include patients without bone lesions.
5.1. Inclusion Criteria (#8)	The inclusion criterion for participants who have received prior docetaxel treatment was revised to include participants who received last dose of docetaxel ≤3 months prior to randomization.	To allow adequate recovery from prior docetaxel therapy.
5.1. Inclusion Criteria (#9)	The text was amended to extend prior treatment with abiraterone acetate plus prednisone (AAP) (from 30 to 45 days).	To increase run in for AAP to allow inclusion of a greater number of participants from referrals.
	Prior treatment with ketoconazole for prostate cancer was allowed for a maximum of two weeks in the metastatic castration-sensitive prostate cancer (mCSPC).	To allow inclusion of a greater number of participants.

Section Number and Name	Description of Change	Brief Rationale
5.1. Inclusion Criteria (#10)	The text was amended for allowed prior treatments for localized prostate cancer.	To better define allowed prior treatments for localized prostate cancer and time frames.
5.2. Exclusion Criteria (#3)	The exclusion criterion of prior AAP treatment timeline was modified.	To allow inclusion of a greater number of participants.
5.2. Exclusion Criteria (#18)	Patients with moderate hepatic impairment Class B were also excluded along with patients with severe hepatic impairment Class C per the Child-Pugh classification system.	To exclude patients with moderate or severe hepatic impairment by Child-Pugh criteria.
5.4. Screen Failures; 8. Study Assessments and Procedures; 10.7. Appendix 7: Clinical Laboratory Tests	Coagulation panel was added to the list of clinical laboratory tests to be performed at screening.	To ensure exclusion of patients with liver dysfunction per Child-Pugh exclusion criteria.
6.1. Study Medication(s) Administered	The definition of study medication was revised.	To include Fixed Dose Combination (FDC) (Niraparib + AA) as a study medication
6.5. Dose Modification	The text related to non-hematologic toxicities due to niraparib or AA was added and table for only niraparib dose modifications due to non-hematologic adverse events was deleted.	To align with other niraparib protocols and to add general non-hematologic guidelines for both niraparib and AA for sites to reference (previous table only had guidelines for niraparib).
	The text “If hypokalemia persists despite optimal potassium supplementation and adequate oral intake, the dose of prednisone may be increased by 5 mg/day and documented in the study medication electronic case report form.” was added.	To indicate that prednisone can be used for all toxicity grades.
6.5. Dose Modification (Table 8)	The hypertension guidelines were deleted.	No longer needed as dose modification for hypertension is now covered by the general non-hematologic toxicity management instructions.
6.5. Dose Modification Hematological Toxicities	The text was modified to delete “hemoglobin”.	To clarify inconsistencies in toxicity management.
6.8.1. Permitted Supportive Care and Interventions	The text ‘Increase of prednisone to 10 mg/daily if needed to manage mineralocorticoid toxicity related to AA’ was added.	To allow physician discretion to manage mineralocorticoid toxicity related to AA as needed.
6.8.1. Permitted Supportive Care and Interventions<br>6.8.2. Prohibited Concomitant Therapies	The text “Bisphosphonates or denosumab at doses for osteoporosis prophylaxis” was deleted.<br>The text “Bisphosphonates or denosumab for management of bone metastasis unless such therapy was started >28 days prior to randomization and participants have been on a stable dose.” was deleted.	As the risk-benefit for bisphosphonates supports allowance as concomitant therapy to prevent potential bone complications, particularly due to associated toxicity from long term ADT, this restriction has been removed.
6.8.2. Prohibited Concomitant Therapies	Ketoconazole was removed as an example from the list of prohibited concomitant therapies.	Already covered under CYP17 inhibitors.

Section Number and Name	Description of Change	Brief Rationale
8. Study Assessments and Procedures	The number of cycles was corrected from '24 to 41'.	To correct the mistake and align with the average number of cycles versus patient population.
	The text 'or initiation of subsequent therapy' was deleted from the Follow-up Phase.	To continue to monitor patients, regardless of subsequent treatment, until meets primary end point of radiographic progression-free survival (rPFS).
8.2.2. Vital Signs	The text 'blood pressure and heart rate monitoring can occur at clinic visit or may also be reported to the site by a home nurse or other qualified medical professionals approved by the investigator and authorized to examine patients.' was added.	To provide further instructions on home monitoring.
8.3.7. Adverse Events of Special Interest	The adverse event 'Cataract' was deleted from the list of adverse events of clinical interest and 'Major Adverse Cardiovascular Events (MACE)' were added.	To update the list of adverse events of clinical interest based on the safety findings from other niraparib studies.
10.5. Appendix 5: Guidance on Study Conduct During the COVID-19 Pandemic	The text related to COVID vaccine was added.	To provide guidance related to COVID vaccine.
10.7. Appendix 7: Clinical Laboratory Tests	The footnote was edited to add "to ensure patient still meets eligibility criteria."	To ensure safety labs are drawn within 14 days of first dose.
10.9. Appendix 10: Open-label Extension Phase<br>10.10. Appendix 11: Long-term Extension Phase	These appendices are added and numbers were updated.	To add details of the open-label and long-term extension phases in Appendix 10 and Appendix 11, respectively.
10.12. Appendix 12: Protocol Amendment History	The protocol amendment 1 summary table was moved to Appendix 12.	To align with the template requirement.
Throughout the protocol	Minor grammatical, formatting, editorial, or spelling changes were made.	Minor errors are noted.

Amendment 1 (21 December 2020)

Overall Rationale for the Amendment: To update safety monitoring and guidance based on updates to niraparib core safety information.

Section number and Name	Description of Change	Brief Rationale
1.1. Synopsis (Overall Design, Treatment Groups and Duration); 4.1. Overall Design	The word 'basis' was deleted, and 'ratio' was added.	To specify the proportion throughout protocol.
1.1. Synopsis (Treatment Groups and Duration); 6.1.2. Study Medication	The words 'Experimental' and 'Control' were added.	To define the study drug combinations.
4.2. Scientific Rationale for Study Design	The word 'comparator' was deleted, and 'control' was added.	
1.1. Synopsis (Biomarker Evaluations); 1.3. Schedule of Activities (Table 1); 4.2. Scientific Rationale for Study Design; 5.1. Inclusion Criteria (#5)	The text regarding local tests for biomarker evaluations was modified.	To clarify collection of archival and fresh tissue samples only if local laboratory results are provided at prescreening.
5. Study Population	Figure 2 Determination of Molecular Eligibility was updated.<br>Prescreening eligibility criterion (#4) was modified.	
1.1. Synopsis (Biomarker Evaluations); 1.3. Schedule of Activities (Table 1); 4.2. Scientific Rationale for Study Design; 5. Study Population	Definition for local test was added.	To clarify the local test.
1.1. Synopsis (Pharmacogenomic [DNA] Evaluations); 8.5. Genetics	The text 'or somatic' was deleted.	To clarify that this pertains only to germline mutations.
1.1. Synopsis (Pharmacogenomic [DNA] Evaluations); 8.5. Genetics	The word 'optional' related to research samples was deleted.	To specify that research sample is not optional
1.2. Schema (Figure 1)	The specific mention of assays to determine gene eligibility was deleted from the schematic overview of the study.	To accommodate participants with genes selected using any genetic tests rather than any specific test.
1.3. Schedule of Activities (Table 1)	The text 'If not eligible by tissue, can be enrolled if germline (blood or saliva) result is positive' was deleted.	To clarify that patients are eligible for enrollment by germline results if tissue results are negative or inconclusive.
1.3. Schedule of Activities (Table 2)	The treatment cycles 4 to 12 and 13 to 24 were merged.	To combine the cycles with similar schedule of activities.
	Cycle 25 was moved to the next phase of treatment (till end-of-treatment [EoT]).	

Section number and Name	Description of Change	Brief Rationale
1.3. Schedule of Activities (Table 2); 8. Study Assessments and Procedures	The ±7 days window for cycles 13 to 25 was shortened to ±3 days.	To reduce visit window to align with earlier cycles.
1.3. Schedule of Activities (Table 2);	The randomization visit at Cycle 1 Day 1 was deleted.	To specify that randomization will occur before Cycle 1 Day 1.
1.3. Schedule of Activities (Table 2); 8.1.2.1. Patient Reported Outcomes	The text specifying completion of patient reported outcomes (PRO) remotely at follow-up visit was deleted.	To clarify that all the visits have remote option.
1.3. Schedule of Activities (Table 2)	The PRO measurement system 'Patient-reported Outcomes Common Terminology Criteria for Adverse Events (PRO-CTCAE)' and text 'PRO-CTCAE for US sites only 'were added. The text 'during the follow-up phase' was deleted.	To evaluate symptomatic toxicity in participants in the United States (US) as a pilot.
3. Objectives and Endpoints	The PRO measurement system 'PRO-CTCAE' was added.	
8.1.2.1.4. PRO-CTCAE	The text related to PRO-CTCAE was added	
1.3. Schedule of Activities (Table 2); 8.2.2. Vital Signs	Added additional timepoints for vital signs (blood pressure [BP] and heart rate) measurements; additionally, methods for BP collection were clarified.	To align with niraparib Investigator's brochure (IB) and product information.
1.3. Schedule of Activities (Table 2)	Added weekly hematology assessments for the first month (Cycle 1).	To align with niraparib product information.
	The text 'Potassium on Day 15 of Cycle 1, 2, and 3' was deleted from chemistry assessments and 'potassium' was added along with hematology.	To clarify that potassium assessment will be performed at the same timepoints as hematology.
	The text 'prior to randomization', 'while patient is on androgen deprivation therapy (ADT)', and 'these prostate-specific antigen (PSA) samples can be obtained as SOC or as part of the Covance screening tests' was added in the notes column.	If patients start ADT prior to randomization, PSA is going to be captured as part of SOC.
	Coagulation panel and the text related to its sample collection timepoint was added.	To ensure exclusion of patients with coagulation abnormalities at screening.
	The text in the notes for plasma for ribonucleic acid (RNA) and deoxyribonucleic acid (DNA) 'End-of-treatment (EoT) samples should be collected when feasible' was added.	To clarify the collection of plasma for RNA and DNA sample.
	The treatment cycles 1 to 3 were merged for biomarker analysis.	To specify that biomarker analysis could be conducted from Cycle 1 to Cycle 3.
	Modifications were made to specify participants willingness to provide archival or fresh tissue sample and non-requirement of sample collection from participants with results from sponsor's commercial tumor tissue test. The text 'However, it is not a protocol violation if no tissue is available or cannot be collected' was deleted from the notes.	To clarify the expectations on tumor collection from participants who do not undergo sponsor's biomarker testing.
	The text 'point without constituting a protocol deviation' was deleted.	To delete specific mention of protocol deviation.

Section number and Name	Description of Change	Brief Rationale
2. Introduction; 2.3. Benefit-Risk Assessment; 11. References	The IB for niraparib/ abiraterone acetate (AA) fixed dose combination (FDC) was referenced in the text and citation was added in the reference list.	To include niraparib/AA FDC IB.
6.1.2. Study Medication	'Pharmacy Manual' was deleted and IB for niraparib/ AA FDC was referenced in the text and citation was added in the reference list.	
2.2.3 Summary of Available Clinical Data for Niraparib and AA, Plus Prednisone in Prostate Cancer	The text 'is synergistic' was changed to 'may be beneficial'.	To align with the language in other documents.
4.2. Scientific Rationale for Study Design	The specific mention of letter as mode of communication for any biomarker-related enrollment changes to investigator was deleted.	To keep open any mode of communication for reporting any biomarker-related enrollment changes to the investigator.
	Additional information regarding biomarker collection was added.	To update the biomarker collection information.
5. Study Population	The text regarding local tests for biomarker evaluations was deleted.	To avoid repetition.
5.1. Inclusion Criteria (#3)	Inclusion criterion #3 was deleted	
5.1. Inclusion Criteria (#8)	Word 'and' was replaced with 'or'.	To specify that either assessment of imaging or PSA is sufficient, both are not required.
5.1. Inclusion Criteria (#11)	The unit for platelet count was corrected, the serum albumin value at screening was deleted and the text 'as determined by the medical monitor' was deleted.	To align with the health authority requirement.
5.1. Inclusion Criteria (#14 and #15)	Amended text to indicate that study participants must agree to use an adequate contraceptive method, the details of which have been included under Section 5.3. Lifestyle Considerations.<br><br>Clarified restrictions related to sperm donation.	To align with the definitions and methods of contraception described in the Clinical Trials Facilitation and Coordination Group (CTFG) guidelines.
5.3. Lifestyle Considerations	Bullet #3 has been added to further describe highly effective contraceptive methods.	
5.2. Exclusion Criteria (#15)	Criterion #15 was deleted	To avoid repetition.
5.2. Exclusion Criteria (#17 and #18)	New criteria #17 and #18 were added to exclude patients with liver disease and severe hepatic impairment.	To exclude patients with moderate or severe hepatic impairment.
5.4. Screen Failures	The text 'Individuals who do not meet the criteria for participation in this study (screen failure) may be rescreened.' was deleted.	To avoid repetition.
6.1. Study Medication (s) Administered; 6.1.2. Study Medication	The text related to use of single agent product if the FDC formulation is not available was deleted.	To delete the redundant text as now FDC formulation is available.
6.1.2. Study Medication (Table 5)	Text regarding administration of food with prednisone/prednisolone was added.	To clarify food requirement for prednisone/prednisolone.
	Text regarding acceptability of prednisolone in case participants are unable to take prednisone was added.	To clarify substitute acceptability for prednisone/prednisolone.

Section number and Name	Description of Change	Brief Rationale
6.5. Dose Modification	Text 'no dose reduction will be allowed for liver function test (LFT) related toxicity' was deleted.	To correct the error text in the original protocol.
6.5. Dose Modification	Added instruction that niraparib must be discontinued for non-hematologic treatment-related Grade ≥3 toxicities lasting more than 28 days while the participant is administered niraparib 100 mg once daily.	To clarify that the lowest dose of niraparib is 100 mg; further dose reduction is not allowed per protocol.
6.5. Dose Modification (Table 8)	The information regarding general dose reduction guidelines was revised	To update general dose reduction guidelines.
	The dose modification information regarding non-hematologic adverse reactions was added.	To add instructions regarding niraparib withhold/resume/discontinuation in the event of non-hematologic ≥Grade 3 treatment-related adverse reaction and clarify that the lowest dose of niraparib is 100 mg; further dose reduction is not allowed per protocol.
	Hematologic Toxicities Section: added/clarified dosing modification instructions for participants who experience:<br>– Inadequate recovery of platelet counts, neutrophil counts, and/or hemoglobin levels.<br>– Acute myeloid leukemia or myelodysplastic syndrome.	To clarify that niraparib will be discontinued in participants meeting criteria for dose reduction who are already at the lowest dose of niraparib. To clarify instructions for participants who develop bone marrow abnormalities.
	The additional detailed text regarding diagnosis of myelodysplastic syndrome (MDS) was updated.	The management of MDS is updated to be consistent with the latest niraparib Company Core Data Sheet (CCDS).
	An additional action required to be taken on reoccurrence of Grade 3 hypertension was added.	To update the hypertension guidelines.
6.5. Dose Modification (Table 8); 8.3.7. Adverse Events of Special Interest	The text regarding the adverse event of decreased platelet, hemoglobin, and/or neutrophil count was modified.	To clarify that platelet, hemoglobin, and/or neutrophil count can be decreased independently of one another.
6.5. Dose Modification (Table 8)	The word 'prophylactic' was deleted.	To align with the new guidance.
	The word 'should' was deleted and 'must' was added.	
6.5.1. Posterior Reversible Encephalopathy Syndrome (PRES)	Added detailed information regarding posterior reversible encephalopathy syndrome (PRES) and instructions for discontinuation of niraparib if PRES is diagnosed.	To align with recent core labeling changes for niraparib.
6.7. Treatment of Overdose	The information regarding actions to be taken in case of AA overdose was updated.	To align with the United States Prescribing Information (USPI) for ZYTIGA.
	The information regarding actions to be taken in case of prednisone overdose was added.	To update the prednisone treatment overdose information
	The text 'and in the prednisone product information' was added.	To include product information for prednisone.
6.8.2. Prohibited Concomitant Therapies	The text 'other hormonal agents for cancer therapy, antineoplastic agents, radiopharmaceuticals' was deleted.	To avoid repetition.

Section number and Name	Description of Change	Brief Rationale
7.1. Discontinuation of Study Intervention	A cross-reference to Section 6.5 (Dose Modification) was added.	To provide cross-reference to information on adverse events relative to treatment discontinuation.
7.2.1. Withdrawal From the use of Research Samples; 10.2. Appendix 2	The word 'future' was added and 'optional' related to research samples was deleted.	To specify that research sample is not optional, however participant may withdraw consent for use of samples for future research.
	The repetitive text was deleted.	To avoid redundancy.
8. Study Assessments and Procedures	Cycle 25 was changed to Cycle 26.	To align with the schedule of activities (SOA).
8. Study Assessments and Procedures	The text regarding standard of care procedures performed during screening phase was added.	To update standard of care procedures during screening phase
8.1.1.1. Criteria for Primary Endpoint	The word 'baseline' was deleted and 'reference' was added.	To specify the purpose of the scan at Week 8.
8.1.2.1. Patient Reported Outcomes	The text 'side effect bother' was deleted and 'bothered by side effects' was added.	To make grammatical correction.
8.3.4. Regulatory Reporting Requirements for Serious Adverse Events and Anticipated Events	The text 'For the purposes of this study the following serious adverse events (SAEs) will be considered anticipated events' was deleted.<br>The table for anticipated SAEs was deleted.	To delete the outdated list of anticipated SAEs.
8.6.1. Evaluations, 8.6.1.3. Tumor Tissue Analysis	Information regarding exploratory biomarkers was elaborated.	To clarify the information regarding exploratory biomarkers.
8.6.1.1. Plasma for RNA	Text 'will allow evaluation of' was deleted and 'may be used to evaluate' was added.	To mention that the analysis of plasma is optional.
10.1. Appendix 1	The list of abbreviations was updated.	The list is updated to include new terms and abbreviations used in revised text.
10.2. Appendix 2	Word 'will' was deleted and 'may' was added.	To suggest that informed consent for research component is optional.
	Text 'Refusal to participate in the optional research will not result in ineligibility for the study' was deleted.	To suggest that sample collection is mandatory.
10.7. Appendix 7	The text 'fasting' regarding glucose was deleted.	To clarify that any (not particularly fasting) glucose sample can be collected.
	The text 'screening only' regarding albumin was deleted.	To specify that albumin will be collected at every visit with the rest of the clinical chemistry laboratory assessments and not just at screening.
	Added screening international normalized ratio (INR).	To ensure exclusion of patients with coagulation abnormalities in the context of hepatic impairment exclusion criterion #18.
10.8. Appendix 8	Replaced the old table with new table of list of anticipated SAEs.	To align with the Food and Drug Administration (FDA) requirement.
10.9. Appendix 9	New Appendix 9 for Child-Pugh classification system was added and referenced in the exclusion criterion #18.	To update the information regarding the criteria for severe hepatic impairment.

Section number and Name	Description of Change	Brief Rationale
10.10. Appendix 10: Protocol Amendment History	Updated to indicate the 'Summary of Changes Table' for the current amendment is available.	To align with protocol amendment requirements.
5.1. Inclusion Criterion (Criterion #13); 8. Study Assessments and Procedures; 8.3. Adverse Events, Serious Adverse Events, and Other Safety Reporting; 8.3.1. Time Period and Frequency for Collecting Adverse Event and Serious Adverse Event Information; 8.3.3 Follow-up of Adverse Events and Serious Adverse Events; 10.2. Appendix 2; 10.4. Appendix 4; 10.5. Appendix 5	Text has been revised, deleted, or added to align with the Janssen protocol template.<br>Added text to allow remote consenting and monitoring.	To align with the updates made to the Janssen protocol template.
Title Page; Throughout the protocol	Updated the confidentiality statement on the title page and the footer of the protocol.	To update the confidentiality statement and footer per latest template.
Throughout the protocol	'Mutation(s)' has been replaced with 'alteration(s)'at relevant places.<br>Word 'niraparib' was added alongside FDC formulation.	For consistency throughout the protocol.
	Consistent notation has been applied when referring to treatment regimen: niraparib **and** abiraterone acetate (AA) **plus** prednisone (AAP).	For consistency.
	Minor grammatical, formatting, editorial, or spelling changes were made.	Minor errors are noted.

11. REFERENCES

1. Annala M, Struss WJ, Warner EW, et al. Treatment outcomes and tumor loss of heterozygosity in germline DNA repair–deficient prostate cancer. Eur Urol. 2017;72(1):34-42.
2. Annala M, Vandekerkhove G, Khalaf D, et al. Circulating tumor DNA genomics correlate with resistance to abiraterone and enzalutamide in prostate cancer. Cancer Discov. 2018;8(4):444-457.
3. Antonarakis E, Isaacsson-Velho P, Agarwal N, et al. CDK12-altered prostate cancer: Clinical features and therapeutic outcomes to standard systematic therapies, PARP inhibitors, and PD-1 inhibitors. Poster presented at: Annual Congress of the European Society for Medical Oncology, 27 Sep-01 Oct 2019, Barcelona Spain.
4. Bray F, Ferlay J, Soerjomataram I, Siegel RL, Torre LA and Jemal A. Global cancer statistics 2018: GLOBOCAN estimates of incidence and mortality worldwide for 36 cancers in 185 countries. CA Cancer J Clin. 2018;68:394-424.
5. Castro E, Romero-Laorden N, del Pozo A, et al. PROREPAIR-B: A prospective cohort study of the impact of germline DNA repair mutations on the outcomes of patients with metastatic castration-resistant prostate cancer. J Clin Oncol. 2019;37(6):490-503.
6. Cella D, Paul D, Yount S, et al. What are the most important symptom targets when treating advanced cancer? A survey of providers in the National Comprehensive Cancer Network (NCCN). Cancer Invest. 2003;21(4):526-535.
7. Cella D, Nichol MB, Eton D, Nelson JB, Mulani P. Estimating clinically meaningful changes for the Functional Assessment of Cancer Therapy--Prostate: results from a clinical trial of patients with metastatic hormone-refractory prostate cancer. Value Health. 2009;12(1):124-129.
8. Cleeland CS, Ryan KM. Pain assessment: global use of the Brief Pain Inventory. Ann Acad Med Singapore. 1994;23(2):129-38.
9. Crocetti E. Epidemiology of prostate cancer in Europe. Joint Research Centre (online publication) 2015. Accessed on 7 February 2020. Available at: https://publications.jrc.ec.europa.eu/repository/handle/JRC101382.
10. Daut RL, Cleeland CS, Flanery RC. Development of the Wisconsin Brief Pain Questionnaire to assess pain in cancer and other diseases. Pain. 1983;17(2):197-210.
11. de Bono J, Logothetis C, Molina A, et al. Abiraterone and increased survival in metastatic prostate cancer. N Engl J Med. 2011;364(21):1995-2005.
12. de Bono J, Mateo J, Fizazi K, et al. Olaparib for metastatic castration-resistant prostate cancer. N Engl J Med 2020; DOI: 10.1056/NEJMoa1911440.
13. Desai et al. COVID-19 vaccine guidance for patients with cancer participating in oncology clinical trials. Nature Reviews. 2021;18:313 https://doi.org/10.1038/s41571-021-00487-z.
14. Dhawan M, Ryan CJ, Ashworth A. DNA repair deficiency is common in advanced prostate cancer: new therapeutic opportunities. Oncologist. 2016;21(8):940-945.
15. Do K, Chen AP. Molecular pathways: targeting PARP in cancer treatment. Clinical Cancer Research. 2013;19(5):977-984.
16. Esper P, Mo F, Chodak G, et al. Measuring quality of life in men with prostate cancer using the functional assessment of cancer therapy-prostate instrument. Urology. 1997;50(6):920-928.
17. EuroQol. EQ-5D. Available at: https://euroqol.org/eq-5d-instruments/eq-5d-5l-about/. Accessed 30 March 2020.
18. Farmer H, McCabe N, Lord CJ, et al. Targeting the DNA repair defect in BRCA mutant cells as a therapeutic strategy. Nature. 2005;434(7035):917-921.
19. FDA: Drug Development and Drug Interactions: Table of Substrates, Inhibitors, and Inducers. Available at: http://www.fda.gov/Drugs/DevelopmentApprovalProcess/DevelopmentResources/DrugInteractionsLabeling/ucm093664.htm. Accessed 25 March 2020.
20. Fizazi K, Tran N, Fein L, et al. Abiraterone plus prednisone in metastatic, castration-sensitive prostate cancer. N Eng J Medicine. 2017;377(4):352-360.

21. Fizazi K, Tran NP, Fein L. Abiraterone acetate plus prednisone in patients with newly diagnosed high-risk metastatic castration-sensitive prostate cancer (LATITUDE): final overall survival analysis of a randomised, double-blind, phase 3 trial. The Lancet Oncology. 2019;20(5):686-700.

22. Garassino, MC, et al. The ESMO call to action on COVID-19 vaccinations and patients with cancer: Vaccinate. Monitor. Educate. Ann. Oncol. 2021. https://doi.org/10.1016/j.annonc.2021.01.068.

23. Herdman M, Gudex C, Lloyd A, et al. Development and preliminary testing of the new five-level version of EQ-5D(EQ-5D-5L). Qual Life Res 2011;20(10):1727-1736.

24. Investigator's Brochure: Niraparib/Abiraterone Acetate Fixed-dose Combination, edition 4. Janssen Research & Development (04 January 2023).

25. Investigator's Brochure: Niraparib, edition 14. GlaxoSmithKline Research & Development Limited. (21 June 2022).

26. Investigator's Brochure: ZYTIGA® (abiraterone acetate), edition 17. Janssen Research & Development (17 June 2022).

27. Janssen MF, Pickard AS, Golicki D, et al. Measurement properties of the EQ-5D-5L compared to the EQ-5D-3L across eight patient groups: a multi-country study. Qual Life Res 2013;22(7):1717-1727.

28. Janssen Research & Development, LLC. A study of niraparib in combination with abiraterone acetate and prednisone versus abiraterone acetate and prednisone for treatment of participants with metastatic prostate cancer (MAGNITUDE). Accessed on 31 January 2023. Available at: https://clinicaltrials.gov/ct2/show/study/NCT03748641

29. Jones P, Altamura S, Boueres J, et al. Discovery of 2-{4-[(3S)-Piperidin-3-yl]phenyl}-2H-indazole-7-carboxamide (MK-4827): A novel oral poly(ADP-ribose)polymerase (PARP) inhibitor efficacious in BRCA-1 and -2 Mutant Tumors. J Med Chem. 2009;52:7171-7185.

30. Kaelin WG, Jr. The concept of synthetic lethality in the context of anticancer therapy. Nat Rev Cancer. 2005;5(9):689-698.

31. Mallinckrodt C, Lane P. Recommendations for the primary analysis of continuous endpoints in longitudinal clinical trials. Drug Info J. 2008;42:303-319.

32. Mateo J, Boyson G, Barbieri CE, et al. DNA repair in prostate cancer: biology and clinical implications. Eur Urol. 2017;71(3):417-425.

33. Maurer W, Bretz F. Multiple testing in group sequential trials using graphical approaches. J Stat Biopharm Res. 2013;5(4):311-320.

34. Mohler JL, Antonarakis ES, Armstrong AJ et al. NCCN Guidelines, version 2. 2017: Prostate Cancer Updates. Available at http://www.richclinics.com/upload/201702/28/201702281117291392.pdf. Accessed 14 July 2017.

35. Mottet N, De Santis M, Briers E, et al. Updated guidelines for metastatic hormone-sensitive prostate cancer: abiraterone acetate combined with castration is another standard. Eur Urology 2018;73:316-321.

36. Na R, Zheng SL, Han M, et al. Germline mutations in ATM and BRCA1/2 distinguish risk for lethal and indolent prostate cancer and are associated with early age at death. Eur Urol. 2017;17:740-747.

37. National Comprehensive Cancer Network (NCCN). Clinical Practice Guidelines in Oncology, v 4.2019. NCCN August 2019. Accessed on 05 February 2020. Available at: https://www.nccn.org/professionals/physician_gls/default.aspx#prostate.

38. National Comprehensive Cancer Network. Preliminary recommendations of the NCCN COVID-19 Vaccination Advisory Committee* Version 1.0 1/22/2021. NCCN. 2021. Available at: https://www.nccn.org/covid-19/pdf/COVID-19_Vaccination_Guidance_V1.0.pdf.

39. Nombela P, Lozano R, Aytes A, Mateo J, Olmos D, Castro E. BRCA2 and other DDR genes in prostate cancer. Cancers. 2019;11(3):352.

40. O'Donnell A, Judson I, Dowsett M, et al. Hormonal impact of the 17alpha-hydroxylase/C(17,20)-lyase inhibitor abiraterone acetate (CB7630) in patients with prostate cancer. Br J Cancer. 2004;90(12):2317-2325.

41. Pickard AS, Neary MP, Cella D. Estimation of minimally important differences in EQ-5D utility and VAS scores in cancer. Health Qual Life Outcomes. 2007 Dec 21;5:70.

42. Rathkopf DE, Smith MR, de Bono JS, et al. Updated interim efficacy analysis and long-term safety of abiraterone acetate in metastatic castration-resistant prostate cancer patients without prior chemotherapy (COU-AA-302). Eur Urol. 2014;66(5):815-825.

43. Rodriguez F, Harrison RW, Wojdyla D, Mahaffey KW. Lost to follow-up and withdrawal of consent in contemporary global cardiovascular randomized clinical trials. Crit Pathways in Cardiol. 2015;14:150-153.

44. Rubin MA, Demichelis F. The genomics of prostate cancer: emerging understanding with technologic advances. Mod Pathol. 2018;31:S1-S11.

45. Ryan CJ, Smith MR, de Bono JS, et al. Abiraterone in metastatic prostate cancer without previous chemotherapy. N Engl J Med. 2013;368:138-148.

46. Saad F, Chi KM, Shore N, et al. Phase 1b (BEDIVERE) study of niraparib plus androgen receptor-targeted therapy (ART) in patients (pts) with metastatic castration-resistant prostate cancer (mCRPC). Poster presented at: the 2020 American Society of Clinical Oncology (ASCO) Genitourinary Cancers Symposium. February 13-15, 2020; San Francisco, CA.

47. Sandhu SK, Schelman WR, Wilding G, et al. The poly (ADP-ribose) polymerase inhibitor niraparib (MK4827) in BRCA mutation carriers and patients with sporadic cancer: a phase 1 dose-escalation trial. Lancet Oncol. 2013;14(9):882-889.

48. Shahneen Sandhu, Gerhardt Attard, David Olmos, et al. Gene-by-gene analysis in the MAGNITUDE study of niraparib (NIRA) with abiraterone acetate and prednisone (AAP) in patients (pts) with metastatic castration-resistant prostate cancer (mCRPC) and homologous recombination repair (HRR) gene alterations. J Clin Oncol. 2022; 40(16):5020-5020.

49. Scher HI, Morris MJ, Stadler WM, et al. Trial design and objectives for castration-resistant prostate cancer: updated recommendations from the prostate cancer clinical trials working group 3. J Clin Oncol. 2016;34(12):1402-1418.

50. Schiewer MJ, Goodwin JF, Han S, et al. Dual roles of PARP-1 promote cancer growth and progression. Cancer Discov. 2012;2(12):1134-1149.

51. Siegel RL, Miller KD, Jemal A. Cancer statistics, 2019. CA Cancer J Clin. 2019;69:7–34.

52. Smith MR, Saad F, Coleman R, et al. Denosumab and bone-metastasis-free survival in men with castration-resistant prostate cancer: results of a global phase 3, randomised, placebo-controlled trial. Lancet. 2012;379(9810):39-46.

53. Smith MR, Sandhu S, Kelly WK, et al. Pre-specified interim analysis of GALAHAD: A phase 2 study of niraparib in patients (pts) with metastatic castration-resistant prostate cancer (mCRPC) and biallelic DNA repair gene defects (DRD). Poster presented at: ESMO 2019 Congress; September 27 - October 1 2019; Barcelona, Spain.

54. Surveillance, Epidemiology, and End Results (SEER) Program. Cancer stat facts: prostate cancer (2018). Accessed on 7 February 2020. Available at: https://seer.cancer.gov/statfacts/html/prost.html.

55. Sweeney CJ, Chen Y-H, Carducci M, et al. Chemohormonal therapy in metastatic hormone-sensitive prostate cancer. N Eng J Med. 2015;373(8):737-746.

56. WHO technical report series 840 (1994): Requirements for the blood collection, processing, and quality control of blood, blood components, and plasma derivatives. Available at: http://www.who.int/bloodproducts /publications/WHO TRS 840 A2.pdf. Accessed 25 March 2020.

57. Wyatt AW, Annala M, Aggarwal R, et al. Concordance of circulating tumor DNA and matched metastatic tissue biopsy in prostate cancer. J Natl Cancer Inst. 2017;109(12):djx118.

58. ZEJULA® [package insert]. Waltham MA: TESARO, Inc; 2017.

59. ZYTIGA® [package insert]. Horsham, PA: Janssen Biotech, Inc; 2017.

INVESTIGATOR AGREEMENT

I have read this protocol and agree that it contains all necessary details for carrying out this study. I will conduct the study as outlined herein and will complete the study within the time designated.

I will provide copies of the protocol and all pertinent information to all individuals responsible to me who assist in the conduct of this study. I will discuss this material with them to ensure that they are fully informed regarding the study treatment, the conduct of the study, and the obligations of confidentiality.

Coordinating Investigator (where required):

Name (typed or printed): ____________________

Institution and Address: ____________________

____

____

____

Signature: ____________________ Date: ____________________

(Day Month Year)

Principal (Site) Investigator:

Name (typed or printed): ____________________

Institution and Address: ____________________

____

____

____

Telephone Number: ____________________

Signature: ____________________ Date: ____________________

(Day Month Year)

Sponsor's Responsible Medical Officer:

Name (typed or printed): PPD

Institution: Janssen Research & Development

Signature: [electronic signature appended at the end of the protocol] Date: ____________________

(Day Month Year)

Note: If the address or telephone number of the investigator changes during the study, written notification will be provided by the investigator to the sponsor, and a protocol amendment will not be required.

Signature

User	Date	Reason
PPD	29-Aug-2023 13:45:07 (GMT)	Document Approval
