# Supplementary material for: Niraparib and abiraterone acetate plus prednisone for HRR-deficient metastatic castration-sensitive prostate cancer: a randomized phase 3 trial
Source: Nat Med. 2025 Oct 7;31(12):4109–18. doi: 10.1038/s41591-025-03961-8 (PMC12705445; doi:10.1038/s41591-025-03961-8)
Supplement: Supplementary file 2 — Reporting Summary [file 41591_2025_3961_MOESM2_ESM.pdf]

nature portfolio

Corresponding author(s): Gerhardt Attard

Last updated by author(s): May 1, 2025

Reporting Summary

Nature Portfolio wishes to improve the reproducibility of the work that we publish. This form provides structure for consistency and transparency in reporting. For further information on Nature Portfolio policies, see our Editorial Policies and the Editorial Policy Checklist.

Statistics

For all statistical analyses, confirm that the following items are present in the figure legend, table legend, main text, or Methods section.

n/a	Confirmed	
☐	☒	The exact sample size (*n*) for each experimental group/condition, given as a discrete number and unit of measurement
☒	☐	A statement on whether measurements were taken from distinct samples or whether the same sample was measured repeatedly
☐	☒	The statistical test(s) used AND whether they are one- or two-sided<br>*Only common tests should be described solely by name; describe more complex techniques in the Methods section.*
☐	☒	A description of all covariates tested
☐	☒	A description of any assumptions or corrections, such as tests of normality and adjustment for multiple comparisons
☐	☒	A full description of the statistical parameters including central tendency (e.g. means) or other basic estimates (e.g. regression coefficient) AND variation (e.g. standard deviation) or associated estimates of uncertainty (e.g. confidence intervals)
☐	☒	For null hypothesis testing, the test statistic (e.g. F, t, r) with confidence intervals, effect sizes, degrees of freedom and P value noted<br>*Give P values as exact values whenever suitable.*
☒	☐	For Bayesian analysis, information on the choice of priors and Markov chain Monte Carlo settings
☐	☒	For hierarchical and complex designs, identification of the appropriate level for tests and full reporting of outcomes
☒	☐	Estimates of effect sizes (e.g. Cohen's d, Pearson's r), indicating how they were calculated

Our web collection on statistics for biologists contains articles on many of the points above.

Software and code

Policy information about availability of computer code

Data collection	No software was used.
Data analysis	No software was used.

For manuscripts utilizing custom algorithms or software that are central to the research but not yet described in published literature, software must be made available to editors and reviewers. We strongly encourage code deposition in a community repository (e.g. GitHub). See the Nature Portfolio guidelines for submitting code & software for further information.

Data

Policy information about availability of data

All manuscripts must include a data availability statement. This statement should provide the following information, where applicable:

- Accession codes, unique identifiers, or web links for publicly available datasets
- A description of any restrictions on data availability
- For clinical datasets or third party data, please ensure that the statement adheres to our policy

Janssen Pharmaceutical Companies of Johnson & Johnson's data sharing policy is available at https://www.janssen.com/clinical-trials/transparency. As noted on this site, requests for study data access can be submitted through the Yale Open Data Access (YODA) Project site at http://yoda.yale.edu. After completion of the trial, the data will be posted in YODA.

Research involving human participants, their data, or biological material

Policy information about studies with human participants or human data. See also policy information about sex, gender (identity/presentation), and sexual orientation and race, ethnicity and racism.

Reporting on sex and gender	Only male participants with prostate cancer were recruited for this study. Gender was not collected.
Reporting on race, ethnicity, or other socially relevant groupings	Race and age were collected as reported by the participants as part of the baseline characteristics. Race and age can be used as a covariates in statistical analysis when relevant (eg, pharmacokinetic analyses).
Population characteristics	Male participants >18 years of age (or the local legal age of consent) with deleterious germline or somatic HRR gene-altered metastatic prostate cancer. Among the 696 randomized patients, the median age was 68 years (range: 40 to 88) in the niraparib +AAP arm and 67 years (range: 40 to 92) in the placebo + AAP arm. Most subjects were white (72.3%), had high volume disease (77.6%) and no prior docetaxel (84.2%).
Recruitment	Patients were recruited at the various sites. The Sponsor has provided the study sites with optional patient recruitment material templates, to be used in accordance with local regulations and policies.
Ethics oversight	The protocol was approved by the review board at each participating institution and health authorities in every participating country. The trial was conducted in accordance with the International Conference on Harmonisation guidelines for Good Clinical Practice and the principles of the Declaration of Helsinki. All patients provided written informed consent.

Note that full information on the approval of the study protocol must also be provided in the manuscript.

Field-specific reporting

Please select the one below that is the best fit for your research. If you are not sure, read the appropriate sections before making your selection.

☒ Life sciences ☐ Behavioural & social sciences ☐ Ecological, evolutionary & environmental sciences

For a reference copy of the document with all sections, see nature.com/documents/nr-reporting-summary-flat.pdf

Life sciences study design

All studies must disclose on these points even when the disclosure is negative.

Sample size	Approximately 692 participants were to be randomized in a 1:1 ratio to receive niraparib + AAP or AAP, and it was estimated that approximately half of all enrolled participants will have BRCA mutations. It was estimated that approximately 261 rPFS events will be required in the ITT population to provide 91% power for detecting a HR of 0.64 (median rPFS of 33 months for the AAP treatment arm versus 51.6 months for the niraparib + AAP treatment arm) at a 2-sided significance level of 0.025. The study randomized 696 participants and the analysis of the primary endpoint was performed after 264 events.
Data exclusions	No data were excluded.
Replication	Not applicable as this is a clinical trial
Randomization	Participants who meet all the inclusion criteria and none of the exclusion criteria were stratified and then randomized in a 1:1 ratio to receive niraparib + AAP or AAP using permuted block randomization. Participants were stratified by HRR gene status (BRCA2 altered vs CDK12 altered vs all other pathogenic alterations), prior docetaxel use (yes vs no), and volume of disease (high vs low).
Blinding	This was a double-blind study. Blinded treatments were used to reduce potential bias during data collection and evaluation of clinical endpoints.

Reporting for specific materials, systems and methods

We require information from authors about some types of materials, experimental systems and methods used in many studies. Here, indicate whether each material, system or method listed is relevant to your study. If you are not sure if a list item applies to your research, read the appropriate section before selecting a response.

Materials & experimental systems

n/a	Involved in the study	
☒	☐	Antibodies
☒	☐	Eukaryotic cell lines
☒	☐	Palaeontology and archaeology
☒	☐	Animals and other organisms
☐	☒	Clinical data
☒	☐	Dual use research of concern
☒	☐	Plants

Methods

n/a	Involved in the study	
☒	☐	ChIP-seq
☒	☐	Flow cytometry
☒	☐	MRI-based neuroimaging

Clinical data

Policy information about clinical studies

All manuscripts should comply with the ICMJE guidelines for publication of clinical research and a completed CONSORT checklist must be included with all submissions.

Clinical trial registration	NCT04497844
Study protocol	The latest version of the protocol is included in the submission as supplementary information
Data collection	Patients in AMPLITUDE were enrolled in sites from 29 countries/territories. Participants were enrolled between December 2020 and July 2023. The data cutoff was January 7, 2025.
Outcomes	This analysis reports on radiographic PFS (by investigator and BICR) as well as overall survival, time to symptomatic progression, time to subsequent therapy, and safety. These endpoints were pre-defined in the protocol.

Plants

Seed stocks	N/A
Novel plant genotypes	N/A
Authentication	N/A
